# Expression of *3-hydroxy-3-methylglutaryl-CoA reductase*, *p-hydroxybenzoate-m-geranyltransferase *and genes of phenylpropanoid pathway exhibits positive correlation with shikonins content in arnebia [*Arnebia euchroma *(Royle) Johnston]

**DOI:** 10.1186/1471-2199-11-88

**Published:** 2010-11-21

**Authors:** Ravi S Singh, Rishi K Gara, Pardeep K Bhardwaj, Anish Kaachra, Sonia Malik, Ravi Kumar, Madhu Sharma, Paramvir S Ahuja, Sanjay Kumar

**Affiliations:** 1 Biotechnology Division, Institute of Himalayan Bioresource Technology (Council of Scientific and Industrial Research), Palampur (Himachal Pradesh)-176061, India; 2 Endocrinology Division, Central Drug Research Institute, Lucknow (Uttar Pradesh)-226001, India

## Abstract

**Background:**

Geranyl pyrophosphate (GPP) and *p*-hydroxybenzoate (PHB) are the basic precursors involved in shikonins biosynthesis. GPP is derived from mevalonate (MVA) and/or 2-*C*-methyl-D-erythritol 4-phosphate (MEP) pathway(s), depending upon the metabolite and the plant system under consideration. PHB, however, is synthesized by only phenylpropanoid (PP) pathway. GPP and PHB are central moieties to yield shikonins through the synthesis of *m*-geranyl-*p*-hydroxybenzoate (GHB). Enzyme *p*-hydroxybenzoate-*m*-geranyltransferase (PGT) catalyses the coupling of GPP and PHB to yield GHB.

The present research was carried out in shikonins yielding plant arnebia [*Arnebia euchroma *(Royle) Johnston], wherein no molecular work has been reported so far. The objective of the work was to identify the preferred GPP synthesizing pathway for shikonins biosynthesis, and to determine the regulatory genes involved in the biosynthesis of GPP, PHB and GHB.

**Results:**

A cell suspension culture-based, low and high shikonins production systems were developed to facilitate pathway identification and finding the regulatory gene. Studies with mevinolin and fosmidomycin, inhibitors of MVA and MEP pathway, respectively suggested MVA as a preferred route of GPP supply for shikonins biosynthesis in arnebia. Accordingly, genes of MVA pathway (eight genes), PP pathway (three genes), and GHB biosynthesis were cloned. Expression studies showed down-regulation of all the genes in response to mevinolin treatment, whereas gene expression was not influenced by fosmidomycin. Expression of all the twelve genes vis-à-vis shikonins content in low and high shikonins production system, over a period of twelve days at frequent intervals, identified critical genes of shikonins biosynthesis in arnebia.

**Conclusion:**

A positive correlation between shikonins content and expression of *3-hydroxy-3-methylglutaryl-CoA reductase *(*AeHMGR*) and *AePGT *suggested critical role played by these genes in shikonins biosynthesis. Higher expression of genes of PP pathway was a general feature for higher shikonins biosynthesis.

## Background

Shikonins are red naphthoquinone pigments, which possess anti-microbial, anti-inflammatory and anti-tumour activities [1]. These are active ingredient in several pharmaceutical and cosmetics preparations, and used as dye for fabrics and food items [1,2]. Commercially, shikonins are extracted from roots of *Lithospermum erythrorhizon *with 0.14-1.09% yield [2]. Arnebia [*Arnebia euchroma *(Royle) Johnston (family, Boraginaceae)] is another plant species that yields 1.58-1.94% shikonins from roots [3]. The plant is naturally distributed in drier regions of Asia and Northern Africa [4]. In India, it is well distributed in cold desert areas of Lahaul and Spiti district of Himachal Pradesh (latitude 32° 44' 57"-32° 59' 57" N; longitude 76° 46' 29" -78° 41' 34" E) at an altitude of >3800 m above mean sea level [5].

**Figure 1:**
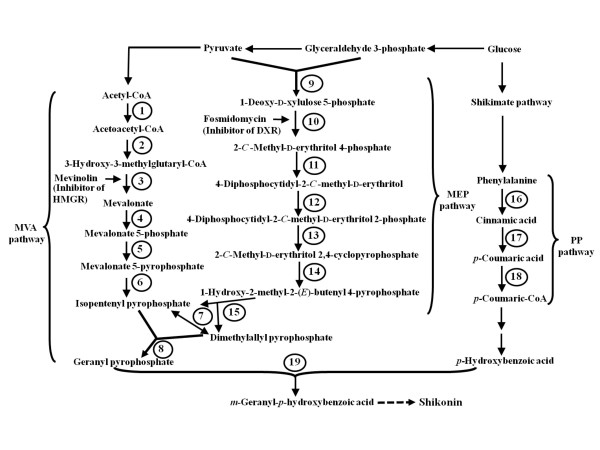
**Shikonin biosynthesis pathway as adopted and modified from Inouye et al. **[38]. Encircled numbers represent enzyme catalyzing the corresponding reaction step as follows: 1 ACTH: acetoacetyl-CoA thiolase; 2 HMGS: 3-hydroxy-3-methylglutaryl-CoA synthase; 3 HMGR: 3-hydroxy-3-methylglutaryl-CoA reductase; 4 MVK: mevalonate kinase; 5 PMVK: phosphomevalonate kinase; 6 MVDD: mevalonate diphosphate decarboxylase; 7 IPPI: isopentenyl pyrophosphate isomerase; 8 GDPS: geranyl diphosphate synthase; 9 1-deoxy-D-xylulose 5-phosphate synthase; 10 1-deoxy-D-xylulose 5-phosphate reductoisomerase; 11 2-*C*-methylerythritol 4-phosphate cytidyl transferase; 12 4-(cytidine-5'-diphospho)-2-*C*-methylerythritol kinase; 13 2-*C*-methylerythritol-2,4-cyclophosphate synthase; 14 1-hydroxy-2-methyl-2-(*E*)-butenyl 4-diphosphate synthase; 15 1-hydroxy-2-methyl-2-(*E*)-butenyl 4-diphosphate reductase; 16 PAL: phenylalanine ammonia lyase; 17 C4H: cinnamic acid 4-hydroxylase; 18 4-CL: 4-coumaroyl-CoA ligase; 19 PGT: *p*-hydroxybenzoate -*m*-geranyltransferase.

Shikonins are composed of *p*-hydroxybenzoate (PHB) and an isoprenoid moiety derived from geranyl pyrophosphate (GPP). PHB is synthesized through phenylpropanoid (PP) pathway, whereas GPP can be synthesized through cytosolic mevalonate (MVA) [6] and plastid 2-*C*-methyl-D-erythritol 4-phosphate (MEP) [7] pathway. In MVA pathway (Figure 1), three molecules of acetyl-CoA couple to yield 3-hydroxy-3-methylglutaryl-CoA (HMG-CoA), which is reduced by the enzyme HMG-CoA reductase (HMGR) to yield MVA. In the next two steps, mevalonate kinase (MVK) and mevalonate 5-phosphate kinase (PMVK) catalyses conversion of MVA to form mevalonate 5-diphosphate (MVD), which in turn is decarboxylated to yield isopentenyl pyrophosphate (IPP). IPP is converted into geranyl pyrophosphate (GPP) using the enzyme geranyl diphosphate synthase (GDPS).

MEP pathway involves condensation of pyruvate and glyceraldehyde 3-phosphate to yield 1-deoxy-D-xylulose 5-phosphate (DXP) using the enzyme DXP synthase (DXS). DXP yields 2-*C*-methyl-D-erythritol 4-phosphate (MEP) by a reaction catalysed by DXP reductoisomerase (DXR), and MEP is then transformed into IPP [8,9] followed by its conversion into GPP, as in the MVA pathway.

Supply of GPP is critical in realizing the yield of isoprenoids [10], therefore, study on regulation of gene expression in GPP biosynthesis is of immense significance. Depending upon the metabolite and species under consideration, the preference for the route to GPP biosynthesis might differ. For example, natural rubber relies on MVA pathway, whereas stevioside is derived through MEP pathway [11,12].

GPP and PHB are coupled through a reaction catalysed by *p*-hydroxybenzoate-*m*-geranyltransferase (PGT) to yield *m*-geranyl-*p*-hydroxybenzoate (GHB; Figure 1), which later leads to the biosynthesis of shikonins [13,14]. Earlier work on shikonins biosynthesis was carried out in *L. erythrorhizon *using one gene of the MVA pathway (*HMGR*), three genes of PP pathway (*PAL*, *C4H*, and *4CL*), and *PGT*. *HMGR *and *PGT *were shown to be regulatory genes in *L. erythrorhizon *[14,15]. Information on other genes in relation to shikonins biosynthesis, however, has not been reported for *L. erythrorhizon*. Arnebia is an important source for shikonins with no molecular data on any of the genes involved in shikonins biosynthesis. Also, the relative importance of MVA or MEP pathway in relation to shikonins is not yet reported. Therefore, the present research was carried out in arnebia to (a) identify the major GPP synthesizing pathways (MVA versus MEP) contributing to shikonins biosynthesis, (b) clone various genes of the pathway, and (c) understand expression regulation of the genes of the identified pathway. The knowledge so generated will help to understand molecular basis of shikonins biosynthesis in arnebia and would lay basis of metabolic engineering for this important moiety.

## Results

### Inhibitor studies suggested MVA as a preferred pathway for shikonins biosynthesis

To facilitate inhibitor studies, protocols for cell suspension culture were developed wherein shikonins content could be modulated. Shikonins content was recorded in traces (3.58 mgl^-1^) in suspension cultures maintained in growth medium [low shikonins production system (LSPS)]. The content, increased to 67.3 mgl^-1 ^at day 1 of transfer of cultures from growth medium into production medium M9 [high shikonins production system (HSPS)]. Shikonins content increased to 478.8 mgl^-1 ^at day 10 of the transfer to production medium M9 and declined thereafter, possibly due to senescence. The shikonins content remained in traces in LSPS during the entire period of experimentation.

At day zero (i.e. the day of transfer of culture from growth to production medium), when shikonins content was negligible, mevinolin (inhibitor of MVA pathway) and fosmidomycin (inhibitor of MEP pathway) were added separately at three different concentrations (50, 100 and 200 μM) and the samples were harvested at day 4 and day 8 of the treatment.

**Figure 2:**
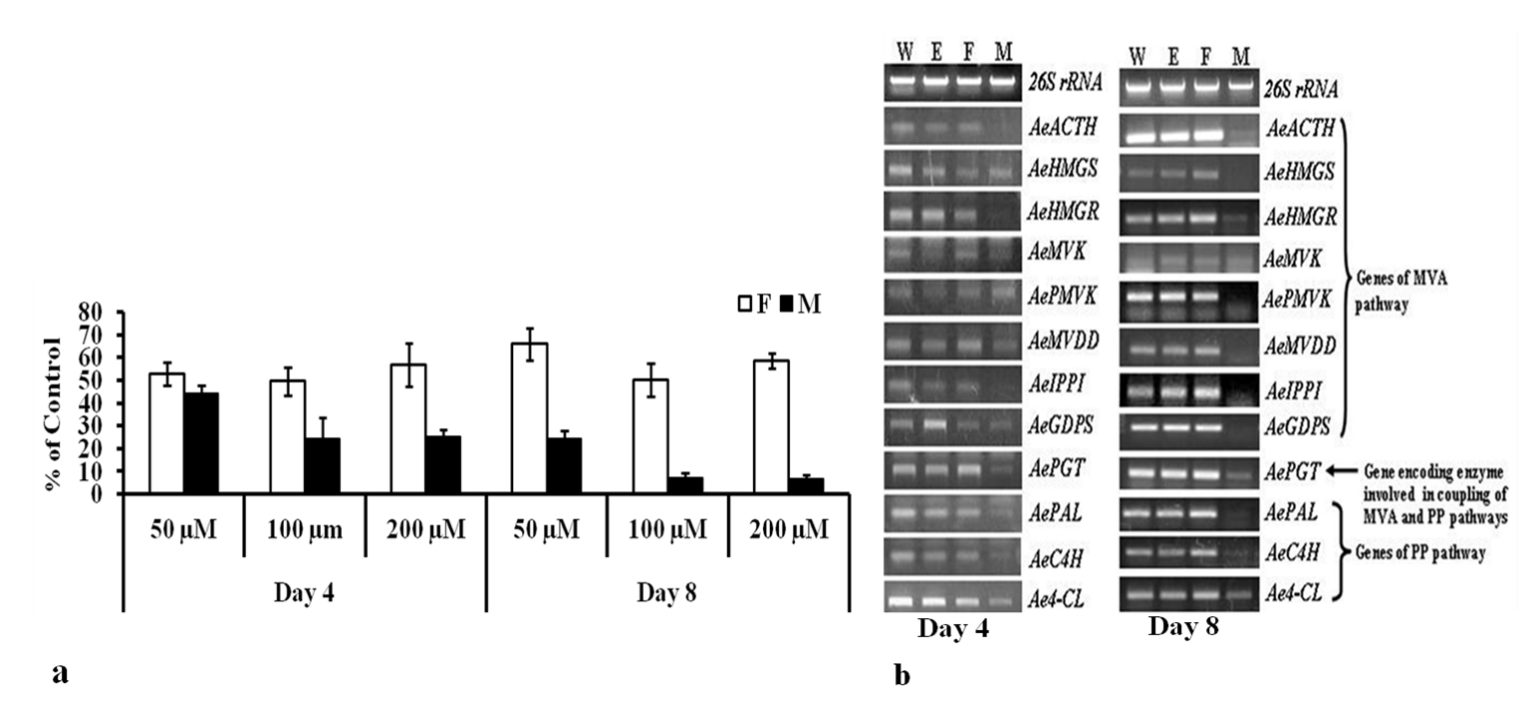
**Effect of inhibitors of mevalonate (MVA) and 2-*C*-methyl-D-erythritol 4-phosphate (MEP) pathway on (a) shikonins biosynthesis, and (b) gene expression at day 4 and day 8 of the treatment**. Mevinolin is an inhibitor of MVA pathway while fosmidomycin inhibits MEP pathway. Different concentrations of mevinolin (M; 50, 100, 200 μM) and fosmidomycin (F; 50,100, 200 μM) were added to the cell suspension culture medium (M9) at day zero (i.e. the day of transfer of culture from growth to production medium). Two controls were also set up, control W had water and control E had ethanol. Concentration of ethanol in E was the same as used for dissolving mevinolin; this was 0.2%, 0.4% and 0.8% in the respective control for 50, 100 and 200 μM of mevinolin. Shikonins content was estimated in cell suspension cultures at day 4 and 8 of the treatment. All the values for shikonins content are shown as mean of three separate experiments with error bar representing standard deviation. Effect of inhibitors [mevinolin (100 μM) and fosmidomycin (100 μM); this was the optimal concentration as evident from experiments in panel a] on gene expression as studied through semi-quantitative RT-PCR at day 4 and day 8 of the treatment. Name of genes is shown on right side of the panel in abbreviated form with their expanded form mentioned in legend of Figure 1. A bar diagram indicating intensities (integrated density value; IDV) of the amplicons of Figure 2b, as measured using Alpha DigiDoc 1000 software, is shown in Additional file 7: Supplementary Figure S5. *26s rRNA *was used as an internal control as shown previously [50].

Lower concentration (50 μM) of mevinolin was found to be less effective as compared to the higher concentrations (100 and 200 μM) to inhibit shikonins production (Figure 2a). A similar level of inhibition observed in the presence of 100 and 200 μM of mevinolin suggested 100 μM to be optimal concentration for further experimentation. Of the day 4 and day 8 of the treatment, maximum inhibition was observed at day 8 of the inhibitor treatment wherein shikonins content was lower by 92.82% of the control (Figure 2a). However, shikonins content was not reduced so drastically in the presence of fosmidomycin and the value was 50.03% of the control at day 8 of the treatment. These results suggested the predominant role of MVA pathway in shikonins biosynthesis in arnebia.

### Twelve genes associated with MVA pathway, PP pathway and GHB biosynthesis were cloned

Since MVA was a predominant pathway for GPP supply for shikonins biosynthesis, all the genes of the pathway were cloned along with those associated with PP pathway and GHB biosynthesis. Degenerate primers for *AeACTH*, *AeHMGS*, *AeMVK*, *AePMVK*, *AeMVDD*, *AeGDPS*, *AeIPPI*, *AePAL*, *AeC4H *and *Ae4-CL *yielded 540 bp, 413 bp, 474 bp, 286 bp, 495 bp, 620 bp, 361 bp, 809 bp, 270 bp and 531 bp sized amplicons, respectively (Additional file 1: Supplementary Table S1). Though in polymerase chain reaction (PCR) several amplicons were obtained in some of the cases, but the amplicons mentioned above were the ones which exhibited strong homology with the reported genes in the database. Partial gene sequences allowed designing of primers for rapid amplification of cDNA ends (RACE) to clone full-length genes. As mentioned in Methods section, availability of expressed sequence tag (EST) for *AeHMGR *and *AePGT *in the EST databank, facilitated designing of primers of these two genes for RACE.

Size and accession number of all the partial and full-length cDNAs are mentioned in Additional file 2: Supplementary Table S2. Ten full-length cDNAs namely, *AeACTH *(1.636 Kb), *AeHMGR *(2.007 Kb), *AePMVK *(1.745 Kb), *AeMVDD *(1.576 Kb), *AeIPPI *(894 bp), *AeGDPS *(1.483 Kb), *AePGT *(1.203 Kb), *AePAL *(2.380 Kb), *AeC4H *(1.767 Kb) and *Ae4-CL *(2.121 Kb) could be cloned. In spite of several attempts, full-length cDNAs of *AeMVK *and *AeHMGS *could not be cloned. Nucleotide and deduced amino acid sequences of the cloned genes are provided in Additional file 3: Supplementary Figure S1, whereas sequence alignment with the respective reported sequences from other plants is provided in Additional file 4: Supplementary Figure S2. Characteristics domains in deduced amino acid sequences of the genes are mentioned in Additional file 5: Supplementary Figure S3. SOPMA (Self-Optimized Prediction Method with Alignment) analysis for prediction of secondary structures in terms of helices, β turns, extended strands and random coils is provided in Additional file 6: Supplementary Figure S4).

Deduced amino acid sequence of all the genes possessed the required motifs and secondary structures (Additional file 5: Supplementary Figure S3 and Additional file 6: Supplementary Figure S4), which are essential to render characteristic functionality, as described previously in other plant systems. These examples include *ACTH *from *Raphanus sativus *[16]; *HMGS *from *Brassica juncea *[17]; *HMGR *[18]; *MVK *[19], *MVDD *[20], *IPPI *[21], *4-CL *[22], and *PAL *[23] from *Arabidopsis thaliana*; *GDPS *from *Abies grandis *[24]; *C4H *from *Populus trichocarpa *× *Populus deltoides *[25] and *PGT *from *L. erythrorhizon *[14].

### Down-regulation of all the twelve genes in response to mevinolin treatment

Amplicons of *AeACTH*, *AeHMGS*, *AeHMGR, AePMVK, AeMVDD, AeGDPS, AeIPPI, AePGT, AePAL, AeC4H *and *Ae4-CL *exhibited down-regulation starting from day 4 of the mevinolin treatment. At day 8, amplicons were hardly visible in mevinolin treated cells. The genes expressed well in untreated control throughout the experimentation period suggesting extreme down-regulation of the genes in response to mevinolin treatment. Unlike mevinolin, fosmidomycin did not affect expression of these genes throughout the experimentation period [Figure 2b; Additional file 7: Supplementary S5 shows integrated density value (IDV) of the amplicons as in Figure 2b]. At day 8 of the treatment, correlation coefficient between shikonins content and IDV of the amplicons was found to be 0.87 (*AeACTH*), 0.77 (*AeHMGS*), 0.71 (*AeHMGR*), 0.62 (*AeMVK*), 0.89 (*AePMVK*), 0.82 (*AeMVDD*), 0.83 (*AeIPPI*), 0.83 (*AeGDPS*), 0.88 (*AePGT*), 0.83 (*AePAL*), 0.68 (*AeC4H*), and 0.73 (*Ae4-CL*) indicating a positive correlation between the shikonins content and gene expression (Table 1).

**Table 1:** Correlation coefficient between gene expression and shikonins content in arnebia.

Name of the gene	Gene expression in cell culture treated with mevinolin	Gene expression in HSPS
**MVA pathway**		

*AeACTH*	0.87	0.31

*AeHMGS*	0.77	0.48

*AeHMGR*	0.71	0.95

*AeMVK*	0.62	0.44

*AePMVK*	0.89	0.28

*AeMVDD*	0.82	0.20

*AeIPPI*	0.83	0.04

*AeGDPS*	0.83	0.39

**PP pathway**		

*AePAL*	0.83	0.57

*AeC4H*	0.68	0.59

*Ae4-CL*	0.73	0.50

**Gene for coupling of MVA and PP pathways**		

*AePGT*	0.88	0.91

**Figure 3:**
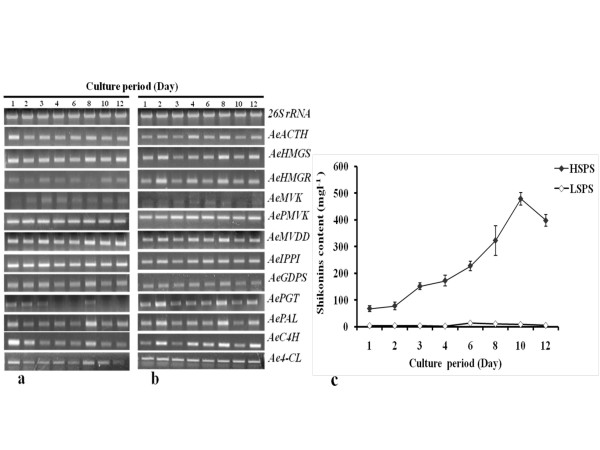
**Expression of various genes in (a) low shikonins producing system (LSPS), and (b) high shikonins producing system (HSPS) vis-à-vis shikonins content (c)**. Shikonins content was very low in HSPS at day 1 and increased thereafter till day 10, whereas LSPS did not show significant accumulation of shikonins (c). Details on LSPS and HSPS are mentioned in Methods section. All the values for shikonins content are shown as means of three separate experiments with error bar representing standard deviation. Name of genes is shown on right side of the panel in abbreviated form with their expanded form mentioned in legend of Figure 1. A bar diagram indicating intensities (integrated density value; IDV) of the amplicons of Figure 3a and 3b, as measured using Alpha DigiDoc 1000 software, is shown in Additional file 8: Supplementary Figure S6a, b. *26s rRNA *was used as an internal control as shown previously [50].

Correlation coefficient was calculated using integrated density value (IDV) of gene expression and the shikonins content in response to the inhibitor (Figure 2) and HSPS (Figure 3), separately. IDV is mentioned in Additional file 7: Supplementary Figure S5 and Additional file 8: Supplementary Figure S6. For Figure 3 linear portion of increase in shikonins content, i.e. day 2, day 4, day 6 and day 8, were considered and accordingly the IDV of gene expression

### Kinetics of gene expression vis-à-vis shikonins content in LSPS and HSPS identified critical genes

Shikonins content in HSPS was 67.3, 77.6, 151, 172, 227.6, 322.6, 478.8 and 397.4 mgl^-1 ^at day 1, 2, 3, 4, 6, 8, 10 and 12, respectively of sub-culturing of suspension culture into production medium. However in LSPS, shikonins content at above days was 3.58, 3.76, 3.24, 2.3, 13.2, 10.5, 8.2 and 4.8 mgl^-1^, respectively (Figure 3c). Analysis of all the twelve genes in HSPS and LSPS on the above days showed evident up-regulation of *AeHMGR *at day 2 onwards and the expression was 94-148% in HSPS as compared to LSPS.

*AePGT *showed up-regulation by 141-184.5% in HSPS as compared to LSPS. The genes of PP pathway namely, *AePAL*, *AeC4H *and *Ae4-CL *also exhibited higher up-regulation of gene expression in HSPS as compared to LSPS at day 2 onwards. Expression of *AeACTH, AeHMGS, AeMVK, AePMVK, AeMVDD, AeIPPI *and *AeGDPS *was very similar in LSPS and HSPS during the entire period of experimentation (Figure 3a, b and Additional file 8: Supplementary Figure S6 showing IDV of Figure 3a, b). The correlation coefficient between shikonins content and IDV of the amplicons in HSPS had a value of 0.95, 0.91, 0.57, 0.59, and, 0.50 for *AeHMGR*, *AePGT*, *AePAL*, *AeC4H *and *Ae4-CL*, respectively (Table 1).

## Discussion

The PP and isoprenoid pathways synthesize array of secondary metabolites, which perform diverse functions in plants ranging from growth and development to defense. Some of these compounds have economic values and also have implications to human health. Red coloured, bioactive shikonins are such molecules which are used in food, fabric and pharmaceutical industries [1,2].

Chemically, shikonins are composed of isoprenoid (e.g. GPP) and PHB moieties, which are also referred to as yield determining moieties [14]. Plants have single PP pathway for the synthesis of PHB, whereas GPP is synthesised through MVA and MEP pathways. The preference for MVA versus MEP pathway depends upon the metabolites and the plant species under consideration [11,12]. Coupling of the two moieties, GPP and PHB, is mediated through the enzyme PGT encoded by *PGT*.

### Biosynthesis of shikonins predominantly uses MVA pathway for GPP supply

Relative contribution of MVA versus MEP pathway towards shikonins biosynthesis was assessed by using mevinolin and fosmidomycin, which are inhibitors of HMGR (of MVA pathway) and DXR (of MEP pathway), respectively [26,27]. For such a study, a cell suspension culture-based system was developed wherein shikonins content increased from negligible quantity by 82.8 fold from day zero to day 10 of transferring the suspension cultures from LSPS to HSPS (Figure 3c). Inclusion of mevinolin produced a severe inhibition (92.82%) in shikonins accumulation (Figure 2a), whereas fosmidomycin produced a comparatively milder (49.97%) inhibition. Data thus implies a prominent role of MVA pathway in shikonins biosynthesis, though a role of MEP pathway can not be completely ruled out. The results are slightly different from *L. erythrorhizon *in which mevinolin inhibited the shikonins biosynthesis by 98% [28] suggesting that GPP for shikonins biosynthesis was derived from the MVA pathway. Involvement of both MVA and MEP pathway in terpenoid biosynthesis has been reported for dolichols biosynthesis in *Coluria geoides *[29], which might also be the case in arnebia.

### Mevinolin down-regulated expression of all the twelve genes involved in GPP and GHB biosynthesis

Mevinolin is a known inhibitor of the enzyme HMGR. Down-regulation of expression of *HMGR *mRNA was reported in *L. erythrorhizon *in response to mevinolin treatment [15]. Information on the effect of mevinolin on expression of other genes of shikonins biosynthesis pathway is not available in *L. erythrorhizon*. Our data showed that mevinolin down-regulated expression of all the eight genes involved in MVA pathway, all the three gene of PP pathway and *AePGT *(Figure 2b). Application of fosmidomycin had no effect on expression of these genes. Data suggested a substrate/product mediated feed-back and feed-forward regulation of the genes under study. For *HMGR*, its post-transcriptional and post-translational feedback regulation was suggested in the experiments using arabidopsis treated with lovastatin (analogous to mevinolin) [30]. Previously, Dixon et al. [31] reported that endogenous cinnamate (product of PAL) caused inhibition of both transcription and enzymatic activity of PAL. Rani et al. [32] reported catechin-mediated down-regulation of several genes of PP pathway. In animal system, MVA pathway involved in the biosynthesis of cholesterol, has long been known to be regulated by end-product feedback inhibition and this regulation has been attributed to direct regulation of the expression of several cholesterol biosynthetic genes by the sterol sensing sterol regulatory element (SRE) binding protein-2 (SREBP-2) [33]. In yeast and higher plants, MVK was subjected to feed-back inhibition by GPP and FPP [34,35], suggesting feed-back inhibition by prenyl phosphates as a general regulatory mechanism to modulate the activity of MVK.

Existing literature thus supports the view of substrate mediated feedback inhibition, possibly that has been observed for *HMGR, MVK *and *PAL*. Overall down-regulation of other genes in response to mevinolin and fosmidomycin needs further investigations on substrate/product mediated feed-back and feed-forward regulation of the genes.

### Not all genes exhibited up-regulation in HSPS

LSPS and HSPS provided a convenient system to study the possible regulatory genes in shikonins biosynthesis. Analysis of all the twelve genes showed that *AeHMGR *(of MVA pathway), and *AePGT *(involved in coupling of GPP and PHB) exhibited evident up-regulation in HSPS as compared to LSPS from day 2 onwards. Generally an up-regulation of all the genes of PP pathway was noticed in HSPS as compared to the LSPS. Earlier reports suggested HMGR enzyme to play a key role in the control of isoprenoid biosynthesis in plants [36,37]. Lange et al. [15] proposed the importance of HMGR as an early enzyme in isoprenoid biosynthesis to control the metabolic flux into the MVA pathway, in contrast to the stringently regulated later enzymes controlling the biosynthesis of specific end products. Our data also suggested that *AeHMGR *could be an important regulatory gene in shikonins biosynthesis, since a strong positive correlation (r, 0.95) was obtained between its expression and the shikonins content (Table 1).

A requirement of higher PP pathway appeared to be general feature for shikonins biosynthesis in arnebia as evidenced by higher expression of the three genes (Figure 3). PP supplies PHB which is a substrate involved in shikonins biosynthesis [38,39]. While in *L. erythrorhizon *genes of PP pathway did not exhibit any specific trend in relation to shikonins biosynthesis [40], data on arnebia appears to be in line with the previous reports on tea [32,41] wherein all the three genes of PP pathway were reported to be regulatory.

*AePGT *encodes for PGT enzyme involved in GHB biosynthesis. This gene also showed up-regulation (three times) in HSPS and was positively correlated (r, 0.91) with shikonins content. Heide and Tabata [42] reported high (thirty five times) activity of PGT enzyme in shikonins producing culture extracts of *L. erythrorhizon *as compared to the non-shikonins producing system suggesting PGT to be important in regulating of biosynthesis of shikonins.

## Conclusion

Arnebia prominently uses MVA pathway for the synthesis of GPP to be utilized in shikonins biosynthesis. Analyses of all the twelve genes suggested the importance of *AeHMGR*, all the genes of PP pathway and *AePGT *in realizing the shikonins yield in arnebia. This is the first report wherein twelve genes of shikonins biosynthesis pathway have been analysed to identify the regulatory genes and hence have implications in synthetic biology for shikonins production.

## Methods

### Plant material

Plants of arnebia [*Arnebia euchroma *(Royle) Johnston] were procured from Kibber (a niche site of arnebia; latitude 32° 20' 11" N; longitude 78° 00' 52"E; 4200 m above mean sea level) in Lahaul and Spiti district of Himachal Pradesh, India and maintained in plastic pots (20 cm height x 20 cm top width x 12 cm bottom width) containing soil, sand, and farm yard manure mixture in a ratio of 2:1:1 at the Institute at Palampur (latitude 32° 06' 32" N; longitude 76° 33' 43" E; 1300 m above mean sea level).

### Establishment of arnebia cell suspension cultures and inhibitor experiment

Shoot cultures were raised using rhizome buds as explants after surface sterilization with 0.04% mercuric chloride. The medium used was agar gelled Murashige and Skoog medium (MS) [43] supplemented with kinetin (Kn: 5.0 μM). The leaves from *in vitro *shoots were cut into 0.3-0.5 cm^2 ^segments and inoculated with their adaxial surface in contact with agar gelled MS + 6-benzyl aminopurine (BAP: 10.0 μM) + indole-3-butyric acid (IBA: 5.0 μM) in Petri plates (90 mm). The pH of the medium was adjusted to 5.8 prior to autoclaving at 121°C for 20 minutes. The callus was maintained by regular sub-culturing at 30 day interval on the same medium and incubated in culture room at 25 ± 2°C under dark conditions. Sub-culture resulted into the formation of friable callus. Friable callus was used for raising cell suspension cultures. Callus (2-3 g fresh weight) was inoculated in 250 ml Erlenmeyer flask containing 30 ml of growth medium (MS with 10 μM BAP and 5 μM of IBA). The cultures were kept on shaker set at 100 rpm at 25°C under dark conditions and sub-cultured at 8 day interval. Cell biomass was maintained either on "growth medium" (Table 2) to get low-shikonins producing system (LSPS) or to "production medium" (M9 medium; Table 2; [44]) to get high-shikonins producing system (HSPS).

**Table 2:** Composition of growth medium and production medium used for arnebia cell suspension culture.

S. No.	Constituents	Concentration (mgl^-1^)	
		
		Growth medium	Production medium (M9)
1.	CaCl_2_.2H_2_O	440.0	--

2.	Ca(NO_3_)_2_.7H_2_O	--	694.0

3.	NH_4_NO_3_	1650.0	--

4.	MgSO_4_.7H_2_O	370.0	750.0

5.	KH_2_PO_4_	170.0	--

6.	KNO_3_	1900	80.0

7.	Na_2_EDTA.2H_2_O	37.2	19.0

8.	Na_2_SO_4_	--	1480.0

9.	FeSO_4_.7H_2_O	27.8	27.8

10.	Na_2_MoO_4_.2H_2_O	0.25	--

11.	H_3_BO_3_	6.2	4.5

12.	CoCl_2_.6H_2_O	0.025	--

13.	CuSO_4_.5H_2_O	0.025	0.3

14.	ZnSO_4_. 7H_2_O	8.6	3.0

15.	KCl	--	65.0

16	MnSO_4_.H_2_O	16.9	--

17.	KI	0.83	--

18.	Mesoinositol	100	--

19.	Glycine	2.0	--

20.	Nicotinic acid	0.5	--

21.	Pyridoxine HCl	0.5	--

22.	Thiamine HCl	0.11	--

23	Sucrose	30000	30000

24.	6-Benzyl amino-purine (BAP)	10.0 μM	--

25	Indole-3-butyric acid (IBA)	5.0 μM	--

Growth medium is referred to as low shikonins production system (LSPS), whereas production medium constitutes high shikonins production system (HSPS)

In order to find out the possible role of MVA versus MEP pathway in shikonins production, different concentrations (50, 100 and 200 μM) of mevinolin (inhibitor of MVA pathway; dissolved in 100% ethanol and later added to the medium) [26] and fosmidomycin (inhibitor of MEP pathway; dissolved in water) [27] was added separately at the time of inoculation of cell biomass to M9 medium. Accordingly, two controls were used. One had equivalent concentration of ethanol as used for dissolving mevinolin (0.2%, 0.4% and 0.8% in respective control of 50, 100 and 200 μM mevinolin), and for other control equal volume of water, as used for dissolving fosmidomycin, was added to the medium. Cell suspension cultures were kept on shaker set at 100 rpm at 25°C under dark conditions. Samples were harvested at day 4 and day 8 of the treatment for shikonins analysis and gene expression studies.

### Estimation of total shikonins

The production of shikonins in cell suspension cultures was measured as described by Yazaki et al. [45]. For the purpose, 1 ml medium was aspirated from cell suspension culture and 500 μl of isoamyl alcohol was added followed by gentle shaking for 5 minutes, and allowed to settle till two layers were formed. A portion (200 μl) from the upper oily layer, was pipetted out to which 800 μl of freshly prepared KOH (2.5%) was added followed by gentle shaking for 5 minutes. After a while two layers got separated and the blue colored lower layer (500 μl) was used to record absorbance at 620 nm on a spectrophotometer (SPECORD 200, Analytik Jena, AG, Germany). A calibration curve was prepared using shikonin (Life Technologies, India) as standard.

### Cloning of cDNAs involved in the biosynthesis of GPP, PHB and GHB

Degenerate primers were designed (Additional file 1: Supplementary Table S1) to clone *AeACTH, AeHMGS, AeMVK, AePMVK, AeMVDD, AeGDPS, AeIPPI, AePAL*, *AeC4H*, and *Ae4-CL*, based upon the conserved regions of corresponding genes reported for different plants. Total RNA was isolated from root tissue of arnebia using Colinmin™ and the iRIS™ system as described earlier [46-48]. RNA was treated with DNase I (amplification grade; Invitrogen, USA) and used for the synthesis of first strand complementary DNA (cDNA) using 0.5 μg oligo-d (T)_12-18 _primer (Invitrogen, USA) and 200 U Superscript II reverse transcriptase (Invitrogen, USA) in a total volume of 20 μl. Gene was amplified by PCR using 1 μl of cDNA template (prepared using RNA isolated from root tissue), 0.2 μM each of forward and reverse primers, 0.2 mM of dNTPs, 1 U of Taq DNA polymerase, and 1× PCR buffer in a final volume of 25 μl. Additional file 1: Supplementary Table S1 lists PCR conditions used to amplify the partial cDNAs. Amplified products were ligated into a pGEM^®^-T Easy vector (Promega, USA) and transformed into DH5α *E. coli *cells using the standard protocol. The transformed cells were spread on LB-ampicillin (100 μg/ml) plate along with isopropyl β-D-1-thiogalactopyranoside (IPTG) and 5-bromo-4-chloro-3-indolyl-β-D-galactopyranoside (X-Gal). All the white (transformed) colonies were re-streaked on a fresh ampicillin plate and colony PCR was performed to check the cloning of PCR product. PCR-positive colonies were used for isolation of plasmids using GenElute Plasmid Miniprep Kit (Sigma, USA). Sequencing of both the strands was performed using Big Dye terminator v3.1 cycle sequencing mix (Applied Biosystems, USA) on an automated DNA sequencer (ABI PRISM™ 310 and 3130 *xl*, Genetic Analyzer, Applied Biosystems, USA) and analyzed by BLAST algorithm at NCBI database.

Sequence of these partial cDNA was used for cloning of full length cDNAs using rapid amplification of cDNA ends (RACE). Primers for RACE of *AeHMGR *and *AePGT *were designed using their EST available at NCBI database vide accession number GR881971 and GR882046, respectively. Additional file 9: Supplementary Table S3 lists various primers and PCR conditions used for RACE. The 5'- and 3'- RACE-ready cDNAs were prepared and RACE was performed using SMART™ RACE cDNA Amplification Kit: Clontech, USA). Amplified products were cloned and analysed essentially as described above. Manufacturer's instructions were followed as and when necessary and also various protocols, as detailed in Sambrook et al. [49], were followed.

### Bioinformatics analysis

Primers were designed and analyzed using Primer 3 Input (Primer3_www.cgi v.0.2; http://frodo.wi.mit.edu/). Various sequences were aligned using ClustalW2 http://www.ebi.ac.uk/. Homology search was conducted using BLASTN and BLASTX algorithm http://www.ncbi.nlm.nih.gov/. Deduced amino acid sequence was used to analyse protein families and domains using tools available at PROSITE database at ExPASy Proteomics Server http://ca.expasy.org/, SMART http://smart.embl-heidelberg.de/ and NCBI conserved domain search http://www.ncbi.nlm.nih.gov/structure/cdd/. SOPMA http://npsa-pbil.ibcp.fr was used for secondary structure prediction of the deduced protein.

### Semi-quantitative gene expression analysis

Complementary DNA was synthesized essentially as described earlier. PCR was carried out using gene specific primers and expression was evaluated at exponential phase of amplification as described earlier [50]. Additional file 10: Supplementary Table S4 lists all the PCR parameters for reverse transcription-PCR (RT-PCR). Expression of *26S rRNA *was used as internal control to equalize cDNA quantity in various reactions [50]. Gel was viewed on a UV trans-illuminator and captured on gel documentation system (Alpha DigiDoc™, Alpha Innotech, USA). Integrated density value (IDV) of amplicons was calculated by AD-1000 software (Alpha DigiDoc™, Alpha Innotech, USA). The data was used to calculate the relative change in gene expression.

### Gene expression in response to mevinolin and fosmidomycin

To study the gene expression in response to mevinolin and fosmidomycin (100 μM; the most effective concentration), RNA was isolated from arnebia cell suspension culture harvested at day 4 and day 8 day of the treatment, along with their respective controls and the expression was studied as described elsewhere.

### Kinetics of gene expression and shikonins content in LSPS and HSPS

Cells were harvested at day 1, 2, 3, 4, 6, 8, 10 and 12 of transfer into production medium (HSPS). For control, the cells at similar day were harvested from growth medium (LSPS). RNA was isolated and expression analysis was performed by RT-PCR as described earlier. Shikonins content was also estimated on respective days as described elsewhere in the manuscript.

## Authors' contributions

RSS carried out the gene cloning, expression analysis, data collection, data analysis and manuscript writing. RKG and PKB participated in gene cloning and expression analysis. AK participated in gene expression analysis. SM and RK were involved in experiments on cell suspension culture and shikonins estimations under the guidance of MS who also contributed in manuscript writing. PSA guided the research on cell suspension culture. SK conceived the study, designed the experiments, analysed the data, drafted and improved the manuscript. All authors read and approved the final manuscript.

## Supplementary Material

Additional file 1 **Primer sequences and PCR conditions used in the present work for amplifying the desired gene from arnebia**. Degenerate primer sequences and PCR conditions used for amplifying the genes; *AeACTH*, *AeHMGS*, *AeMVK*, *AePMVK*, *AeMVDD*, *AeGDPS*, *AeIPPI*, *AePAL*, *AeC4H*, and *Ae4-CL *from arnebia. Click here for file

Additional file 2 **Size of cDNAs (partial, full-length), BLAST analysis and Domain search in deduced amino acid sequences**. Size of cDNAs (partial, full-length), BLAST analysis and Domain search in deduced amino acid sequences of cDNAs of *AeACTH*, *AeHMGS*, *AeHMGR, AeMVK*, *AePMVK*, *AeMVDD*, *AeGDPS*, *AeIPPI*, *AePGT, AePAL*, *AeC4H*, and *Ae4-CL *from arnebia. Click here for file

Additional file 3 **Nucleotide and deduced amino acid sequence of the genes cloned from arnebia**. Nucleotide and deduced amino acid sequence of the (a) *AeACTH*, (b) *AeHMGS*, (c) *AeHMGR*, (d) *AeMVK*, (e) *AePMVK*, (f) *AeMVDD*, (g) *AeGDPS*, (h) *AeIPPI*, (i) *AePGT*, (j) *AePAL*, (k) *AeC4H *and (l) *Ae4-CL*. The amino acid sequence is represented by single-letter code under each codon. Start and stop codons are indicated by * and **, respectively. Nucleotides in capital letters represent untranslated regions. 'Poly A' signal is shown in bold and underlined at position for *AeACTH *(AAATAAAA)*, AeHMGS *(AAATAACT), *AeHMGR *(TGATAAA)*, AePMVK *(CATTAAAA)*, AeMVDD *(AATAAA)*, AeIPPI *(GAATAAAA)*, AeGDPS *(GAATAAAA)*, AePGT *(AAATAAAT)*, AeC4H *(AAATAATC) and *Ae4-CL *(ATATAAAA). ('Poly A' signal was searched using HCpolya: Hamming Clustering poly-A prediction in Eukaryotic Genes, http://zeus2.itb.cnr.it/~webgene/wwwHC_polya.html). Click here for file

Additional file 4 **Sequence alignment (ClustalW2; http://www.ebi.ac.uk/) of the deduced amino acid sequences of arnebia cDNAs**. Sequence alignment (ClustalW2; http://www.ebi.ac.uk/) of the deduced amino acid sequences of arnebia cDNAs with the respective reported sequences from other plants: (a) AeACTH (GenBank:AAU95618.1*Nicotiana tabacum*, GenBank:ABC74567.1*Picrorhiza kurrooa*, GenBank:AAM00280.1*Arabidopsis thaliana*, GenBank:AAL18924.1*Hevea brasiliensis*); (b) AeHMGS (GenBank:ABX55778.1*Solanum lycopersicum*, GenBank:ACD87446.1*Camptotheca acuminate*, GenBank:EEF51079.1*Ricinus communis*, GenBank:AAS46245.1*Hevea brasiliensis*, GenBank:AAG32923.1*Brassica juncea*); (c) AeHMGR (GenBank:AAL54878.1*Nicotiana tabacum*, GenBank:BAA93631.1*Solanum tuberosum*, GenBank:ABC74565.1*Picrorhiza kurrooa*, GenBank:ABV25901.1*Antirrhinum majus*, GenBank:AAD38873.1*Oryza sativa*, GenBank:AAA33040.1*Camptotheca acuminata*); (d) AeMVK (GenBank:AAN72115.1*Arabidopsis thaliana*, GenBank:ABD32397.1*Medicago truncatula*, GenBank:ABV02026.1*Nicotiana langsdorffii *x *Nicotiana sanderae*, GenBank:ACG46416.1*Zea mays*, GenBank:AAL31086.1*Oryza sativa*); (e) AePMVK (GenBank:BAD44652.1*Arabidopsis thaliana*, GenBank:AAL18926.1*Hevea brasiliensis*, GenBank:CAO63313.1*Vitis vinifera*, GenBank:ABF95008.1*Oryza sativa*, GenBank:NP_001149345.1*Zea mays*); (f) AeMVDD (GenBank:BAF98285.1*Hevea brasiliensis*, GenBank:XP_002521172.1*Ricinus communis*, GenBank:NP_566995.1*Arabidopsis thaliana*, GenBank:ABV02028.1*Nicotiana langsdorffii *x *Nicotiana sanderae*, GenBank:ABW87316.1*Solanum lycopersicum*); (g) AeIPPI (GenBank:BAB40974.1*Nicotiana tabacum*, GenBank:ABX55779.1*Solanum lycopersicum*, GenBank:AAQ84167.1*Pueraria montana *var. lobata, GenBank:AAB94132.1*Camptotheca acuminate*, GenBank:AAF29973.1*Adonis palaestina*); (h) AeGDPS (GenBank:AAS82860.1*Antirrhinum majus*, GenBank:AAF08793.1*Mentha x piperita*, GenBank:AAW66658.1*Picrorhiza kurrooa*, GenBank:ACQ90682.1*Humulus lupulus*, GenBank:AAN01133.1*Abies grandis*, GenBank:ACA21458.1*Picea abies*); (i) AePGT (GenBank:NP_849431.1*Arabidopsis thaliana*, GenBank:ACC91260.1*Capsella rubella*, GenBank:BAD05721.1*Oryza sativa*, GenBank:BAB84122.1*Lithospermum erythrorhizon*); (j) AePAL (GenBank:BAA24929.1*Lithospermum erythrorhizon*, GenBank:ABG75911.1*Nicotiana attenuate*, GenBank:ACF17667.1*Capsicum annuum*, GenBank:BAA95629.1*Catharanthus roseus*, GenBank:AAU08174.1*Camellia sinensis*); (k) AeC4H (GenBank:BAB71716.1*Lithospermum erythrorhizon*, GenBank:ABC69046.1*Solanum tuberosum*, GenBank:ACF19421.1*Capsicum annuum*, GenBank:AAT68775.2*Camellia sinensis*, GenBank:BAF81522.1*Brassica rapa*, GenBank:AAB58355.1*Arabidopsis thaliana*) and (l) Ae4-CL (GenBank:BAA08365.1*Lithospermum erythrorhizon*, GenBank:ACF17632.1*Capsicum annuum*, GenBank:AAD40664.1*Solanum tuberosum*, GenBank:BAA07828.1*Nicotiana tabacum*, GenBank:ACL31667.1*Paulownia fortune*, GenBank:CAP08784.1*Arabidopsis thaliana*, GenBank:ABA40922.1*Camellia sinensis*). Gaps are represented as dashes; asterisks, colons and dots indicate identical amino acid residues, conserved substitutions, and semi-conserved substitutions, respectively. Click here for file

Additional file 5 **Domain and protein families in the deduced amino acid sequences of arnebia cDNAs**. Domain and protein families in the deduced amino acid sequences of *AeACTH*, *AeHMGS*, *AeHMGR*, *AeMVK, AePMVK*, *AeMVDD, AeGDPS, AeIPPI*, *AePGT, AePAL, AeC4H*, and *Ae4-CL*. Click here for file

Additional file 6 **Prediction of secondary structure of deduced amino acid sequences**. Prediction of secondary structure of deduced amino acid sequences of *AeACTH*, *AeHMGS*, *AeHMGR*, *AeMVK, AePMVK*, *AeMVDD, AeGDPS, AeIPPI*, *AePGT, AePAL, AeC4H *and *Ae4-CL *by SOPMA. Helices, sheets, turns and coils are indicated by the longest, the second longest, the second shortest and the shortest vertical lines, respectively. Click here for file

Additional file 7 **Bar diagram indicating intensities (integrated density value; IDV) of the amplicons of Figure **2b** at day 4 and day 8 (panel b) as measured using Alpha DigiDoc 1000 software**. Bar diagram indicating intensities (integrated density value; IDV) of the amplicons of Figure 2b at day 4 and day 8 (panel b) as measured using Alpha DigiDoc 1000 software. Error bar shows standard deviation of three separate values. Since the IDV of separate gels were very different, these were normalized based upon the amplicons for *26 S rRNA*. Click here for file

Additional file 8 **Bar diagram indicating intensities (integrated density value; IDV) of the amplicons of Figure **3a** and **3b** as measured using Alpha DigiDoc 1000 software**. Bar diagram indicating intensities (integrated density value; IDV) of the amplicons of Figure 3a and 3b as measured using Alpha DigiDoc 1000 software. Error bar shows standard deviation of three separate values. Since the IDV of separate gels were very different, these were normalized based upon the amplicons for *26 S rRNA*. Click here for file

Additional file 9 **Primer sequences and PCR conditions used in RACE reactions**. Primer sequences and PCR conditions used in RACE reactions for cloning of full-length cDNAs of *AeACTH*, *AeHMGS, AeHMGR*, *AePMVK*, *AeMVDD, AeGDPS, AeIPPI*, *AePGT, AePAL, AeC4H *and *Ae4-CL*. Click here for file

Additional file 10 **Primer sequences and PCR conditions used in semi-quantitative RT-PCR-based expression analysis**. Primer sequences and PCR conditions used in semi-quantitative RT-PCR-based expression analysis of *AeACTH*, *AeHMGS*, *AeHMGR, AeMVK*, *AePMVK*, *AeMVDD*, *AeGDPS*, *AeIPPI*, *AePGT, AePAL*, *AeC4H*, and *Ae4-CL *in arnebia. Click here for file
