# Supplementary material for: Expression of 3-hydroxy-3-methylglutaryl-CoA reductase, p-hydroxybenzoate-m-geranyltransferase and genes of phenylpropanoid pathway exhibits positive correlation with shikonins content in arnebia [Arnebia euchroma (Royle) Johnston]
Source: BMC Mol Biol. 2010 Nov 21;11:88. doi: 10.1186/1471-2199-11-88 (PMC3002352; doi:10.1186/1471-2199-11-88)
Supplement: Additional file 1 — Primer sequences and PCR conditions used in the present work for amplifying the desired gene from arnebia. Degenerate primer sequences and PCR conditions used for amplifying the genes; AeACTH, AeHMGS, AeMVK, AePMVK, AeMVDD, AeGDPS, AeIPPI, AePAL, AeC4H, and Ae4-CL from arnebia. [file 1471-2199-11-88-S1.PDF]

**Additional file 1:** Supplementary Table S1. Primer sequences[a] and PCR conditions used in the present work for amplifying the desired gene from arnebia.

| Name of the gene | Primer sequence (forward primer, F and reverse primer, R) | PCR conditions |
|---|---|---|
| *AeACTH* | F : 5′-AGRGCAAAYGTBGAKCCR-3′<br>R: 5′-GCAGCWGCACCATCACTTAT-3′ | 35 cycles: 94ºC, 30 sec; 56ºC, 40 sec; 72 ºC, 1 min; Final extension at 72ºC, 7 min |
| *AeHMGS* | F: 5′-GTGWTTCACTCTSCATACWAC-3′<br>R: 5′-TAARATCGACWAATTTMTTCT-3′ | 35 cycles: 94ºC, 30 sec; 52ºC, 40 sec; 72 ºC, 1 min; Final extension at 72ºC, 7 min |
| *AeMVK* | F: 5′-CTRCTCTCCTWGCTTYTTC-3′<br>R: 5′-GTATRCACTGACGGTKTTG-3′ | 35 cycles: 94ºC, 30 sec; 52ºC, 40 sec; 72 ºC, 1 min; Final extension at 72ºC, 7 min |
| *AePMVK* | F: 5′-ATG GCT GTW GTT GCT TCT GC-3′<br>R: 5′- CCATTAMAGGAAGTCATTKC-3′ | 35 cycles: 94ºC, 30 sec; 52ºC, 40 sec; 72 ºC, 1 min; Final extension at 72ºC, 7 min |
| *AeMVDD* | F: 5′-AARTTAAWGAATTTRAAAGAAG-3′<br>R: 5′-GAYTAGTATCWAGGCRAACAG-3′ | 35 cycles: 94ºC, 30 sec; 56ºC, 40 sec; 72 ºC, 1 min; Final extension at 72ºC, 7 min |
| *AeIPPI* | F: 5′-TAYAATTRTCACTTGATSGA-3′<br>R: 5′-ACAWACTTTATRTCAGCYAC-3′ | 35 cycles: 94ºC, 30 sec; 52ºC, 40 sec; 72 ºC, 1 min; Final extension at 72ºC, 7 min |
| *AeGDPS* | R: 5′-CARATCCTTYCCYGCMGTCTTVC-3′<br>F: 5′-GGVGGBAARMGMGTKCGTCCT-3′ | 35 cycles: 94ºC, 30 sec; 56ºC, 40 sec; 72 ºC, 1 min; Final extension at 72ºC, 7 min |
| *AePAL* | F: 5′- CNGARCARCAYAAYCARGT-3′<br>R: 5′- CADATNGGNAGHGGNGC-3′ | 35 cycles: 94ºC, 30 sec; 56ºC, 40 sec; 72 ºC, 1 min; Final extension at 72ºC, 7 min |
| *AeC4H* | F: 5′- CTTMGDATGGGDCARAGGAACYT-3′<br>R: 5′- ATCCKCCTCATTTTMCKCCAGTG-3′ | 35 cycles: 94ºC, 30 sec; 52ºC, 40 sec; 72 ºC, 1 min; Final extension at 72ºC, 7 min |
| *Ae4-CL* | F: 5′- AACTCCCTGATATWTACATCCCWA-3′<br>R: 5′- TGAGAAATGRASACAACCHTC-3′ | 35 cycles: 94ºC, 30 sec; 60ºC, 40 sec; 72 ºC, 1 min; Final extension at 72ºC, 7 min |

[a]R=A/G; M=A/C; W=A/T; Y=C/T; S=C/G; D=A/G/T; H=A/C/T; B=G/C/T; N=A/T/G/C
