# Supplementary material for: Expression of 3-hydroxy-3-methylglutaryl-CoA reductase, p-hydroxybenzoate-m-geranyltransferase and genes of phenylpropanoid pathway exhibits positive correlation with shikonins content in arnebia [Arnebia euchroma (Royle) Johnston]
Source: BMC Mol Biol. 2010 Nov 21;11:88. doi: 10.1186/1471-2199-11-88 (PMC3002352; doi:10.1186/1471-2199-11-88)
Supplement: Additional file 2 — Size of cDNAs (partial, full-length), BLAST analysis and Domain search in deduced amino acid sequences. Size of cDNAs (partial, full-length), BLAST analysis and Domain search in deduced amino acid sequences of cDNAs of AeACTH, AeHMGS, AeHMGR, AeMVK, AePMVK, AeMVDD, AeGDPS, AeIPPI, AePGT, AePAL, AeC4H, and Ae4-CL from arnebia. [file 1471-2199-11-88-S2.PDF]

Additional file 2: Supplementary Table S2. Size of cDNAs (partial, full-length), BLAST analysis and Domain search in deduced amino acid sequences.

S.N.	Name of the gene	Size (partial, full-length cDNAs); Accession no.	BLAST similarity	Domain in deduced amino acid sequence
1.	*AeACTH*	540, 1636 bp; GenBank:DQ395086	*Picrorhiza kurrooa* (81%, GenBank:ABC74567.1)	aminoacyl-transfer RNA synthetases class-I and thiolases active site (389-402 aa)
2.	*AeHMGS*	413 bp (extended to 1244 bp), not cloned; GenBank:DQ395087	*Solanum lycopersicum* (81%, GenBank:ABX55778.1)	hydroxymethylglutaryl-coenzyme A synthase active site (27-42 aa)
3.	*AeHMGR*	700 bp, 2007 bp; GenBank:DQ400696	*Camptotheca acuminate* (69%, GenBank:AAA33040.1)	hydroxymethylglutaryl-coenzyme A reductase signatures (336-553 aa)
4.	*AeMVK*	474 bp, not cloned; GenBank:DQ631831	*Arabidopsis thaliana* (98%, GenBank:AAD45421.1)	putative ATP-binding domain (9-20 aa)
5.	*AePMVK*	286 bp, 1745 bp; GenBank:EU315068	*Hevea brasiliensis* (73%, GenBank:AAL18926.1)	pmev_Kin_ERG8 domain (5-480 aa)
6.	*AeMVDD*	495 bp, 1576 bp; GenBank:DQ631830	*Solanum lycopersicum* (80%, GenBank:ABW87316.1)	GHMP kinases-N domain and GHMP kinases-C (115-173 aa)
7.	*AeIPPI*	361 bp, 894 bp; GenBank:DQ453138	*Camptotheca acuminate* (91% , GenBank:AAB94132.1)	NUDIX domain (50-195 aa)
8.	*AeGDPS*	620 bp, 1483 bp; GenBank:DQ395088	*Picrorhiza kurrooa* (63%, GenBank:AAW66658.1)	polyprenyl synthetases signatures (122-268 aa)
9.	*AePGT*	630 bp, 1203 bp; GenBank:DQ397513	*Lithospermum erythrorhizon* (93%,DDBJ:BAB84122.1)	UbiA prenyltransferase family signature (82-104 aa)
10.	*AePAL*	809 bp, 2380 bp; GenBank:DQ399123	*Lithospermum erythrorhizon* (95%, DDBJ:BAA24928.1)	phenylalanine and histidine ammonia-lyases signature (192-208 aa)
11.	*AeC4H*	270 bp, 1767 bp ; GenBank:DQ417206	*Lithospermum erythrorhizon* (97%, DDBJ:BAB71716.1)	Cytochrome P450 cysteine heme-iron ligand signature (440-449 aa)
12.	*Ae4-CL*	531 bp, 2121 bp;	*Lithospermum erythrorhizon*	putative AMP-binding domain signature

		GenBank:DQ400697	(86%, DDBJ:BAA08365.1)	(189-200 aa)
