# Supplementary material for: Expression of 3-hydroxy-3-methylglutaryl-CoA reductase, p-hydroxybenzoate-m-geranyltransferase and genes of phenylpropanoid pathway exhibits positive correlation with shikonins content in arnebia [Arnebia euchroma (Royle) Johnston]
Source: BMC Mol Biol. 2010 Nov 21;11:88. doi: 10.1186/1471-2199-11-88 (PMC3002352; doi:10.1186/1471-2199-11-88)
Supplement: Additional file 3 — Nucleotide and deduced amino acid sequence of the genes cloned from arnebia. Nucleotide and deduced amino acid sequence of the (a) AeACTH, (b) AeHMGS, (c) AeHMGR, (d) AeMVK, (e) AePMVK, (f) AeMVDD, (g) AeGDPS, (h) AeIPPI, (i) AePGT, (j) AePAL, (k) AeC4H and (l) Ae4-CL. The amino acid sequence is represented by single-letter code under each codon. Start and stop codons are indicated by * and **, respectively. Nucleotides in capital letters represent untranslated regions. 'Poly A' signal is shown in bold and underlined at position for AeACTH (AAATAAAA), AeHMGS (AAATAACT), AeHMGR (TGATAAA), AePMVK (CATTAAAA), AeMVDD (AATAAA), AeIPPI (GAATAAAA), AeGDPS (GAATAAAA), AePGT (AAATAAAT), AeC4H (AAATAATC) and Ae4-CL (ATATAAAA). ('Poly A' signal was searched using HCpolya: Hamming Clustering poly-A prediction in Eukaryotic Genes, http://zeus2.itb.cnr.it/~webgene/wwwHC_polya.html). [file 1471-2199-11-88-S3.PDF]

Additional file 3: Supplementary Figure S1.

(a) ***Acetoacetyl-CoA-thiolase (AeACTH)***

```
                                              1 GGCTATTCTCTGT 13
  14 TTCGTCCATCAATTAAGATAAACTCAAGATTGCTGAGAGTGATCA 58
  59 atggttgaagatagcattaagcctagagatgtgtgcattgttgga 103
     *M  V  E  D  S  I  K  P  R  D  V  C  I  V  G
 104 gttgcaagaaccccgatgggcggttttctcggttcattgtcctct 148
     V  A  R  T  P  M  G  G  F  L  G  S  L  S  S
 149 ttatctgctacagagcttggttccatagctatcaaaagtgctctg 193
     L  S  A  T  E  L  G  S  I  A  I  K  S  A  L
 194 aagagagcaaatgttgatccatctcttgtacaagaagttatcttt 238
     K  R  A  N  V  D  P  S  L  V  Q  E  V  I  F
 239 ggcaatgtgctaagcgcaaatttgggacaggctcccgctagacaa 283
     G  N  V  L  S  A  N  L  G  Q  A  P  A  R  Q
 284 gctgcattgggtgcagggttgcctaatacagttagttgtaccact 328
     A  A  L  G  A  G  L  P  N  T  V  S  C  T  T
 329 gttaacaaagtttgtgcttcagggatgaaagcaaccatgctggca 373
     V  N  K  V  C  A  S  G  M  K  A  T  M  L  A
 374 gcacagactatccaattgggcatcaacgatgttgttgtagccggt 418
     A  Q  T  I  Q  L  G  I  N  D  V  V  V  A  G
 419 ggaatggggagcatgtcaaattgccctaagtaccttgcagaagca 463
     G  M  G  S  M  S  N  C  P  K  Y  L  A  E  A
 464 aggaggggttctcgtcttgggcatgattcccttgtcgatggaatg 508
     R  R  G  S  R  L  G  H  D  S  L  V  D  G  M
 509 ctaaaagatgggttgtgggatgtgtataatgatgttggcatgggc 553
     L  K  D  G  L  W  D  V  Y  N  D  V  G  M  G
 554 gtttgcgctgaaacttgtgctgaaaatcacaaaatctcaagagag 598
     V  C  A  E  T  C  A  E  N  H  K  I  S  R  E
 599 gatcaggataattttgcgagacgaccaggattcatatgcaattca 643
     D  Q  D  N  F  A  R  R  P  G  F  I  C  N  S
 644 aagcttgaacgtggaagggcagcacaaaatagtggtgcattttcc 688
     K  L  E  R  G  R  A  A  Q  N  S  G  A  F  S
 689 tgggaaatagttccggttgagctttccgggagcagaggaaaacca 733
     W  E  I  V  P  V  E  L  S  G  S  R  G  K  P
 734 actgttatcattgataaagatgagggtttaagcaaatttgatgca 778
     T  V  I  I  D  K  D  E  G  L  S  K  F  D  A
 779 tctaggctaaggaaacttaggccaacattcaagcctgatggtggc 823
     S  R  L  R  K  L  R  P  T  F  K  P  D  G  G
 824 tctgttacagctggcaatgcttcaagtataagtgatggggctgct 868
     S  V  T  A  G  N  A  S  S  I  S  D  G  A  A
 869 gcactggttctggtgagcggagagaaggcaatctttttcggactc 913
     A  L  V  L  V  S  G  E  K  A  I  F  F  G  L
 914 catgtgattgcaaagatcagagggtttgccgatgcggctcaggca 958
     H  V  I  A  K  I  R  G  F  A  D  A  A  Q  A
 959 cccgagctgtttacaactgctccagctcttgcaatacctaaagca 1003
     P  E  L  F  T  T  A  P  A  L  A  I  P  K  A
1004 attaaaaattctggtttagttgcatctcaaattgattactatgaa 1048
     I  K  N  S  G  L  V  A  S  Q  I  D  Y  Y  E
1049 ataaatgaagctttctcggttgttgctcttgtgaaccaaagatta 1093
     I  N  E  A  F  S  V  V  A  L  V  N  Q  R  L
1094 ctgaatctcaaatctgaacaactcaacttgcatggtggagcggta 1138
     L  N  L  K  S  E  Q  L  N  L  H  G  G  A  V
1139 tctttaggacatccacttgggtgcagtgatgctcgaattttggtg 1183
     S  L  G  H  P  L  G  C  S  D  A  R  I  L  V
1184 acattattaggggttttgaggcataaaaatgggaagtttggggtt 1228
     T  L  L  G  V  L  R  H  K  N  G  K  F  G  V
1229 gctggagtttgcaatgggggaggtggagcatcagctgttgttttg 1273
```

A G V C N G G G A S A V V L
1274 gagttcatgtcaacagaaaggatgggagtacgctccaaatta**taa** 1318
E F M S T E R M G V R S K L **
1319 GTATGGAGTAATGCACTGTTGTAAAACATGATATCTTAAGAAACC 1363
1364 CAAAAACTATGTAAAGGTGCTACCAAACCCTAGTGGCCACGGGAT 1408
1409 ATTACTCGCCGTCCCGGGTTGGAATTCTTTTTTTTTTTTTTTAAA 1453
1454 TGTGAAGGTGCTACCATAATTACTTGGAAACTAT**AAATAAAA**GGA 1498
1499 GAGATGAAAGATGGGGGAGTTAAAAACTCTCTTCTTTCTCACTTC 1543
1544 TAGTGCAATTCACTCTACACAGAACAAAGCTTATTCGTAGTGTCA 1588
1589 AATCTACTATAAAAAAACAAAAAAAAAAAAAAAAAAAAAAAAAAA 1633
1634 AAA 1636

(b) ***3-hydroxy-3-methylglutaryl-CoA synthase (AeHMGS)***

1 ATTTGTACTGACAGCGCGGTTTATG 25
26 CTGATGGACCTGCTAGGCCAACTGGAGGAGCTGCAGCTGTAGCT 69
70 atgctcatagggcctgatgctccaatcgcattcgaaagcaaattg 114
M L I G P D A P I A F E S K L
115 aggggaagtcatatggcacatgtatatgatttctataagcctaat 159
R G S H M A H V Y D F Y K P N
160 ctagcgagcgagtacccggttgttgatggaaagctctcacagacc 204
L A S E Y P V V D G K L S Q T
205 tgttatttgatggctctagattcttgctacaagacattgtgcaac 249
C Y L M A L D S C Y K T L C N
250 aaatataaaaaattagaaggcaagcaattttcaatttctgatgct 294
K Y K K L E G K Q F S I S D A
295 gattattttgtgtttcactctccatacaacaagcttgtgcataag 339
D Y F V F H S P Y N K L V H K
340 agctttgctcgattgctgttcaatgactttgtgaacaatgccagc 384
S F A R L L F N D F V N N A S
385 tccattgatgaggctgccaaagaaaagtttgcaccttttgcatct 429
S I D E A A K E K F A P F A S
430 ttgactggtgatgaaagttacgcaagccgtgatcttgaaaaggta 474
L T G D E S Y A S R D L E K V
475 gcacagcaagttgcaaaaccattttatgacacaaaggtgcaacca 519
A Q Q V A K P F Y D T K V Q P
520 gctaccctgatcccgaagcaagtcggcaacatgtatacagcatca 564
A T L I P K Q V G N M Y T A S
565 ctttatgcagcttttgcatcactcctccacaacaaaaatagctca 609
L Y A A F A S L L H N K N S S
610 ctggatggaaaacgggtgatgatgttttcatatggcagtgattca 654
L D G K R V M M F S Y G S D S
655 acagccacgatgttttcactccgtcttcgtgagggtcaacagcct 699
T A T M F S L R L R E G Q Q P
700 ttcagcttgtcaaacatagcgactgtcatgaatgtgcagagaagt 744
F S L S N I A T V M N V Q R S
745 ttaagtcaaggcatgagttgcctccagaagaaatttgtcgatctt 789
L S Q G M S C L Q K K F V D L
790 atgcagctgatggagcacagatatggaggtaaagactttgtaaca 834
M Q L M E H R Y G G K D F V T
835 agcaaagactgcagcctcctagctccaggcacatactatctcact 879
S K D C S L L A P G T Y Y L T
880 gaagtcgactccaagtatcgtcggttttactccaagaaggaatcc 924
E V D S K Y R R F Y S K K E S
925 gagaatggaaaattagccaacggtcac**taa**AGCACGACAGAGCAT 969
E N G K L A N G H **
970 GCTATGATGTGCGAAGAATGTCTTCGGCTTCTATCTAAATTCAATA 1015

```
1016 GAATTTGGCAGTATATAGGAAAATAACTGCCTTATTCTACTGAGTT 1061
1062 GCTTCATTTGATATGTAGGACAATGTGTCTTCTTTTGCAGTAAAGT 1107
1108 ACTGATCAGATTATCAACAAAGACCTTTTCATTTTTCTCTTGTATT 1153
1154 GTTAATTGTAAGGAAGATCAAAAGTGGTGAATTTATAATGAAATTG 1199
1200 TGATGTTTGTCAGCTCCAAAAAAAAAAAAAAAAAAAAAAAAAAAAA  1244
```

(c) *3-hydroxy-3-methylglutaryl-CoA synthase (AeHMGR)*

```
   1 GTTCCATCTCTCAAAGTGAAACAATCATCACTTACAATTATTCAATC  47
  48 AATCTTACATATCCCTTCAAAGAAATCAAATATTGTAACTATAGTG   93
  94 atggagtactcaaaatctatgaacaaagataccactagtctcaag 138
     *M  E  Y  S  K  S  M  N  K  D  T  T  S  L  K
 139 aaacaaaatgttgaattagacttttcaagaaaggcttcagaagct 183
     K  Q  N  V  E  L  D  F  S  R  K  A  S  E  A
 184 ttttcattgcctcttcgttttgcgaatgccatcttctttcctttg 228
     F  S  L  P  L  R  F  A  N  A  I  F  F  P  L
 229 ttcttttcagttgtgtattacttgcttatgagatggagggagaag 273
     F  F  S  V  V  Y  Y  L  L  M  R  W  R  E  K
 274 atcagaaactcaacccctcttcatgttgtcagtttccatgagatt 318
     I  R  N  S  T  P  L  H  V  V  S  F  H  E  I
 319 cttgcaatagttgctcttgttgcttcttcaatttatcttcttgga 363
     L  A  I  V  A  L  V  A  S  S  I  Y  L  L  G
 364 tttttaagcattggcttagttcaattatcaggtgaagatgaagaa 408
     F  L  S  I  G  L  V  Q  L  S  G  E  D  E  E
 409 gaggaagaggaagaggttcctctttatgatacaaaggacgtgaac 453
     E  E  E  E  E  V  P  L  Y  D  T  K  D  V  N
 454 aaagatatccaagtcgatcgggtcactctgatggaggaaaaatgt 498
     K  D  I  Q  V  D  R  V  T  L  M  E  E  K  C
 499 catgttgcttgcatttctactgcatctcccaaaccaatggcaaaa 543
     H  V  A  C  I  S  T  A  S  P  K  P  M  A  K
 544 tattgccctgaagaggatgaagagattgtgcaatctgttgtacaa 588
     Y  C  P  E  E  D  E  E  I  V  Q  S  V  V  Q
 589 ggaaatacaccttcttactcgcttgaatcgaagcttggtgattgc 633
     G  N  T  P  S  Y  S  L  E  S  K  L  G  D  C
 634 tttcgggctgcagcaatcagacgtgaggcactacagaggctaact 678
     F  R  A  A  A  I  R  R  E  A  L  Q  R  L  T
 679 ggaaagtcattggaaggacttccacttgagcactttgattacgac 723
     G  K  S  L  E  G  L  P  L  E  H  F  D  Y  D
 724 tctatttgggacagtgttgtgagatgcctatcgggtatgttcag 768
     S  I  L  G  Q  C  C  E  M  P  I  G  Y  V  Q
 769 ctacctgtaggcattgctggaccacttttgctggatgacaaagag 813
     L  P  V  G  I  A  G  P  L  L  L  D  D  K  E
 814 tactcggttcctatggctactaccgaagggtgcttggtagctagt 858
     Y  S  V  P  M  A  T  T  E  G  C  L  V  A  S
 859 accaacagaggatgcaaggcaatatatgcatcgggaggtgctact 903
     T  N  R  G  C  K  A  I  Y  A  S  G  G  A  T
 904 agtgttgtgttgagagatggcatgaccagggcccctgtcgtgagg 948
     S  V  V  L  R  D  G  M  T  R  A  P  V  V  R
 949 tttggcaccgcaaagagagctgctgaactcaagtttttcttggag 993
     F  G  T  A  K  R  A  A  E  L  K  F  F  L  E
 994 gaccctctcaatttcgagacactttctgctgttttcaatcaatct 1038
     D  P  L  N  F  E  T  L  S  A  V  F  N  Q  S
1039 agtagatttggcagacttcagagcattaagtgcgccatcgctggc 1083
     S  R  F  G  R  L  Q  S  I  K  C  A  I  A  G
1084 aagaatctgtacatgaggttctcttgcagcacaggagatgcaatg 1128
     K  N  L  Y  M  R  F  S  C  S  T  G  D  A  M
1129 gggatgaacatggtgtcgaaaggaacacaacatgtaattgagttc 1173
     G  M  N  M  V  S  K  G  T  Q  H  V  I  E  F
1174 ctagagaaggaatttccagacatggatgtcattggcatttctggc 1218
```

L E K E F P D M D V I G I S G
1219 aactattgctctgacaagaaaccggctgcagtgaattggatcgaa 1263
N Y C S D K K P A A V N W I E
1264 ggacgtggaaaatcagtcgtgtgcgaggctatcatcgaagacgtt 1308
G R G K S V V C E A I I E D V
1309 gtgaagaaagtgctcaagacgatgtgctcctgtggagctttacat 1353
V K K V L K T M C S C G A L H
1354 gctcaagaactactgtcgcagtggcggggctctggtggatcaat 1398
A Q E L L S Q W R G L W W I N
1399 gcacatgctagtaacattgtatctgccattttcattgcgacaggt 1443
A H A S N I V S A I F I A T G
1444 caagatccagcccaaaatattgagagttcacattgtatcaccatg 1488
Q D P A Q N I E S S H C I T M
1489 atggaagctgtcaatgaaggaaaagatctccatgtctcggtaacc 1533
M E A V N E G K D L H V S V T
1534 atgccttcgatagaggtagggaccgtcggtggggaactcagctc 1578
M P S I E V G T V G G G T Q L
1579 gcttctcaggcagcatgtttgaatttgctcggagtgaagggggca 1623
A S Q A A C L N L L G V K G A
1624 aatagggagtgcccgggagcaaatgcgaggcaattagcggccata 1668
N R E C P G A N A R Q L A A I
1669 gttgctggatcagttcttgcaggagagctttccctcatgtctgca 1713
V A G S V L A G E L S L M S A
1714 attgctgcaggacaacttgtcaagagccacatgaagtacaacaga 1758
I A A G Q L V K S H M K Y N R
1759 tcaaacaaagatgtttcaaaa**tga**TGTTATGATGATAGAATGCAG 1803
S N K D V S K **
1804 TGCACACGATAATACATAGAAACTGTGGTGAACATAGAAAAGTTAT 1849
1850 TGTAACCCAAATCTTCTTTATTCTTCAAATCTTCTTTATTGTAACC 1895
1896 CAAATCTTCTTTGTTGTCCACCTCAT**TGATAAA**TTAATTTGTTGTC 1941
1942 AATGTAACCATCTGTTTACTTCAAAGTTTCTAGTTATCCAAAAAAA 1987
1988 AAAAAAAAAAAAAAAAAAAA 2007

(d) *Mevalonate Kinase* (*AeMVK*)

1 gctacagtagtcattaactctgagcttccatacgggtctggcctc 45
A T V V I N S E L P Y G S G L
46 ggttcatcagcagctttatgtgtagctctcacagctgctctcctt 90
G S S A A L C V A L T A A L L
91 gcttcttctatttcagagaaaacccgtggtaacggttggtcatct 135
A S S I S E K T R G N G W S S
136 ctcgatgaaaccaatcttgagttgctaaataaatgggctttcgaa 180
L D E T N L E L L N K W A F E
181 ggcgaaaagatcatccatgggaaaccttctgggatagacaacacc 225
E K I I H G K P S G I D N T V
226 gtcagtgcatacggtggcaacatgatcaagttctgctcaggcgag 270
G S A Y G G N M I K F C S G E
270 ataactcggttacaatccaacatgcctctgagaatgctaattacc 315
S N M P L I T R L Q R M L I T
316 aacactagagttgggcgaaacacaaaagctctggtctctggtgtg 360
N T R V G R N T K A L V S G V
361 tcacagagagcggtaagacatcctgatgcggtgaagtcagtgttc 405
S Q R A V R H P D A V K S V F
406 aacgccgtggattctataagcaaagagctcgctgcgatcattcag 450
N A V D S I S K E L A A I I Q
451 tctaaagacgagacctcagttaca 474
S K D E T S V T

(e) *Phosphomevalonate Kinase (AePMVK)*

```
   1 atggctgtagttgcttctgctcctggtaaggttttgatgactgga 45
     *M  A  V  V  A  S  A  P  G  K  V  L  M  T  G
  46 gggtatcttgttttggagaggccaaatgctggaattgttttgagt 90
     G  Y  L  V  L  E  R  P  N  A  G  I  V  L  S
  91 acgaatgctcgattttattcggttgttaagccaatttatgatgaa 135
     T  N  A  R  F  Y  S  V  V  K  P  I  Y  D  E
 136 gttaaaccagacagttgggcttgggcatgggcagatgtgaaatta 180
     V  K  P  D  S  W  A  W  A  W  A  D  V  K  L
 181 acttctccgcaaatgtcaagagaaatgacatacaaattgtctctt 225
     T  S  P  Q  M  S  R  E  M  T  Y  K  L  S  L
 226 aaatatttgacgcttcaaagtgtttctctgagtgattcaagaaac 270
     K  Y  L  T  L  Q  S  V  S  L  S  D  S  R  N
 271 ccatttgtagaatatgcggtgcagtatgttgtggcagcagcatat 315
     P  F  V  E  Y  A  V  Q  Y  V  V  A  A  A  Y
 316 tcaaggcttgactcatctgggaaggatgcacttacgaaactactt 360
     S  R  L  D  S  S  G  K  D  A  L  T  K  L  L
 361 ctacgaggtctagatattacaatattaggttgcaatgagttctac 405
     L  R  G  L  D  I  T  I  L  G  C  N  E  F  Y
 406 tcatatcgaaatcagattgaagctcgtggactgcctctgacacct 451
     S  Y  R  N  Q  I  E  A  R  G  L  P  L  T  P
 451 gaatcattgtcttccctaccaccctttacttcaatcactttcaac 495
     E  S  L  S  S  L  P  P  F  T  S  I  T  F  N
 496 aaagaagagtctggtgggcaaaatagcaaacccgaagttgcaaag 540
     K  E  E  S  G  G  Q  N  S  K  P  E  V  A  K
 541 acagggttgggatcttcagcagctatgactactgcagttgttgct 585
     T  G  L  G  S  S  A  A  M  T  T  A  V  V  A
 586 tctttgcttcattatctgggagttgttaatctttcatcagtaaaa 630
     S  L  L  H  Y  L  G  V  V  N  L  S  S  V  K
 631 gataatagtgaggatcttgatacagtccacatgattgctcagact 675
     D  N  S  E  D  L  D  T  V  H  M  I  A  Q  T
 676 gctcactgcattgcccagggagaagttggaagtgggtttgatgtt 720
     A  H  C  I  A  Q  G  E  V  G  S  G  F  D  V
 721 agctccgctgtttatggcagtcaacgttatgttcggttttcgcca 765
     S  S  A  V  Y  G  S  Q  R  Y  V  R  F  S  P
 766 ggtgtgatttcttctgctcaggatgcagtaaaagcagcaccactt 810
     G  V  I  S  S  A  Q  D  A  V  K  A  A  P  L
 811 gaagaagtcatcaatgacgtcttaaaggctgagtgggaccatgaa 855
     E  E  V  I  N  D  V  L  K  A  E  W  D  H  E
 856 aaggacatgtctcatgctccactgatgacctctattagagagcct 900
     K  D  M  S  H  A  P  L  M  T  S  I  R  E  P
 901 ggaactgggggtcatctacaccatcaatggtaggtgctgtgaag 945
     G  T  G  G  S  S  T  P  S  M  V  G  A  V  K
 946 aagtggcagaaagcggaccctcaaacttcagttgagacttggaga 990
     K  W  Q  K  A  D  P  Q  T  S  V  E  T  W  R
 991 aagttgtcagaagggaatgctgcactcgagatgcaacttaacacc 1035
     K  L  S  E  G  N  A  A  L  E  M  Q  L  N  T
1036 ttaagcaacttggctaggatgagctttgatgtttacaaagatgtt 1080
     L  S  N  L  A  R  M  S  F  D  V  Y  K  D  V
1081 atcaacaactgtagtacactaccttcagagaagtggttagaggta 1125
     I  N  N  C  S  T  L  P  S  E  K  W  L  E  V
1126 gcaactgaacctagccgaacagacattgttaaagcattacttggg 1170
     A  T  E  P  S  R  T  D  I  V  K  A  L  L  G
1171 gcaaaagatgtcatgcttgagatcagatatcaaatgcgcaagatg 1215
     A  K  D  V  M  L  E  I  R  Y  Q  M  R  K  M
1216 ggtgaggctgcaggaataccgatagagccagaatcacaaactctg 1260
     G  E  A  A  G  I  P  I  E  P  E  S  Q  T  L
1261 cttttagactctactatgaatatggaaggagttcttttggctggc 1305
     L  L  D  S  T  M  N  M  E  G  V  L  L  A  G
1306 gtgcctggtgcaggcggatttgatgccgtatttgcagtcactttg 1350
```

V P G A G G F D A V F A V T L

1351 ggtgatgcaagtgacaaggttataaaatcatggagtagacaaaat 1395

G D A S D K V I K S W S R Q N

1396 gttcttgcccttctagtaagagaagatccaaatggggttcttta 1440

V L A L L V R E D P N G V L L

1441 gagaacaatgattcgcgagcaaaggaagttacatctggtgtttct 1485

E N N D S R A K E V T S G V S

1486 gccattcagattcaa**tag** AATTTTTCCAAGTACAACAAGTTT 1527

A I Q I Q **

1528 GGATAGTATAGTATAGTGAGGTCTTGTAAGATTTTATGGTA 1568

1569 TGCAGTAAATCTTATCTACCTGATTTTGATCAGTGCGTTTT 1609

1610 GCCATG**CATTAAAA**TGGCCATTATGTATCCATTGCAGTCAG 1650

1651 GTGCTGCAATCAATTAAATGTAACTTTCGGATGGTAGTGTA 1691

1692 ACACTTATATTTCATTGCGAGTTCGAAGAAAAAAAAAAAAA 1732

1733 AAAAAAAAAAAAA 1745

(f) *Mevalonate diphosphate decarboxylase (AeMVDD*)

1 AAACATTTCTCCACTTCTTTAGATCTCTGAT 31

32 CTACTTCCAATCATCACACAATTTGCTAATTTTTTCCACAAAATT 76

78 TGGTATTTTTTTCATAAATTTTGGTAATTTGTAGAGGAAGAAAAG 121

122 **atg**ggagaacaaggagagaattggattttgatggtgactgcccaa 166

***M** G E Q G E N W I L M V T A Q

167 actcctacaaacatagctgtgataaagtattggggaaaagggat 211

T P T N I A V I K Y W G K R D

212 gagtctttgattttgcctatcaatagtagcattagtgttactctt 256

E S L I L P I N S S I S V T L

257 gatccttctcacctttgcaccaccaccactgtctctgttagccct 301

D P S H L C T T T T V S V S P

302 tcttttaaacaagattgtatgtggcttaatggaaaggaaatctct 346

S F K Q D C M W L N G K E I S

347 ctttctggaggtaggtttcaaagatgtttaagggagatccgatct 391

L S G G R F Q R C L R E I R S

392 cgtgcatgtgatgttgaagatgagaagaagggattcaagatagct 436

R A C D V E D E K K G F K I A

437 aagaaagattgggaaaagcttcatgttcacatcgcttcgtataat 481

K K D W E K L H V H I A S Y N

482 aatttcccaactgctgctggtttggcttcctcagctgctggtttt 526

N F P T A A G L A S S A A G F

527 gcctgtctagtttttttctcttgccaagttaatgaatttgaaagaa 571

A C L V F S L A K L M N L K E

572 gaccacggacagttgtcggctatcgctaggcagggttcaggaagt 616

D H G Q L S A I A R Q G S G S

617 gcttgccgcagcttgtttggaggatttgttaaatgggatatgggt 661

A C R S L F G G F V K W D M G

662 aaagagtcagatggcagtgacagtattgctattccacttgtggac 706

K E S D G S D S I A I P L V D

707 gagaagcactgggatgagcttgtcatcgtgattgcagtggtaagt 751

E K H W D E L V I V I A V V S

752 gcacatcagaaggagacaagtagcacatcaggaatgcgcgacact 796

A H Q K E T S S T S G M R D T

797 gtagagacaagtccactaattcaacatagagcaaaggaagtagtg 841

V E T S P L I Q H R A K E V V

842 cccaaacgcatcgtccaaatggaggaggccataagcaatcgtgat 886

P K R I V Q M E E A I S N R D

887 ttttcgacatttgcccatttgtcttgttcagatagtaatcagttt 931

F S T F A H L S C S D S N Q F

932 cacgctgtttgcctagatactagtccacccatcttttacatgaat 976

H A V C L D T S P P I F Y M N

977 gacacatctcacaggattatcagcctggtcgagaaatggaatcgc 1021
D T S H R I I S L V E K W N R
1022 tccgagggaacacctcaggttgcttatacttttgatgctgggcca 1066
S E G T P Q V A Y T F D A G P
1067 aacgctgctatgattgcacgtaacagaaaggttgctacccttctg 1111
N A A M I A R N R K V A T L L
1112 cttcagaagttgctctattgcttcccgccccaggcagatgctgat 1156
L Q K L L Y C F P P Q A D A D
1157 ttggacagctacgttatcggggataagtcgctacttaaagaagcg 1201
L D S Y V I G D K S L L K E A
1202 ggagttggcaccatgaatgacgtggacgctctggctccacctcca 1246
G V G T M N D V D A L A P P P
1247 gagcttaccactagtgttccagcacaaagaaccaagggagatgta 1291
E L T T S V P A Q R T K G D V
1292 agttacttcatatgcacaagaccagggaaaggtccagttttgcta 1336
S Y F I C T R P G K G P V L L
1337 actgatgaaaatcaagctctcctcgactccaaaactggtctacca 1381
T D E N Q A L L D S K T G L P
1382 aaa**tag**AAGACCAACAGTTTTTGGTGCTTTGTATTGACTTTGTTCT 1427
K ******
1428 GTTGAGA ATATGCATCTTGTGCTTTTCTGTTCTTCTCTTCATTGTA 1473
1474 ATTTAAA TTTTTTCCATTTCCCAGATTGAGAATTGAGACATTGTAT 1519
1520 TAGCAATATATT**AATAAA**AGTTATTTTCAACCTGCAAAAAAAAAAA 1565
1566 AAAAAAAAAAA 1576

(g) *Isopentenyl pyrophosphate isomerase* (*AeIPPI*)

1 GACCATTTCTTGTTCTTCTAACAACAAGATCTCTCTGCTTCATAC 45
46 CAAAATCAAATCAACCCCATTTACCAGATTATTCTCTGCAACCGTC 91
92 atggcagctgatgctggcatggatgctgtccaacgccgtctcatg 136
***M** A A D A G M D A V Q R R L M
137 tttgaagatgaatgcattttggtggatgaaaacgacaatgttgtt 181
F E D E C I L V D E N D N V V
182 ggccatgataccaagtacaattgtcacttgatggaaaaaattgaa 226
G H D T K Y N C H L M E K I E
227 tctgaaaatctgctgcacagggcttttagtgtgtttttgtttaac 271
S E N L L H R A F S V F L F N
272 tcaaagcatgagttgcttctccagcaacggtctgccacaaaggtt 316
S K H E L L L Q Q R S A T K V
317 accttccctctggtatggacaaacacatgttgcagccacccactt 361
T F P L V W T N T C C S H P L
362 tacagggaatccgagcttattaaggaaaattttcttggtgtgagg 406
Y R E S E L I K E N F L G V R
407 aatgctgcacagaggaagctcttggatgagctgggtattcctacg 451
N A A Q R K L L D E L G I P T
452 gaagatgtcccggttgaccaattcactccgttgggtcgcatattg 496
E D V P V D Q F T P L G R I L
497 tacaaagcaccatctgacggcaagtggggagaacatgaacttgat 541
Y K A P S D G K W G E H E L D
542 tatcttctcttcatcgtacgtgatgtaaaggtgaatccgaaccct 582
Y L L F I V R D V K V N P N P
587 gacgaggtggctgatataaagtatgtgaatccggctcagctgaaa 627
D E V A D I K Y V N P A Q L K
628 gaacttttgacaaaagctgatgctggtgaggatggcctgaagctc 672
E L L T K A D A G E D G L K L
673 tccccttggttccgtctcatcgtggacaatttttttgttcaagtgg 717
S P W F R L I V D N F L F K W
718 tgggatcacgttgagaaggggaactaccatcaagttgctgacatg 762
W D H V E K G N Y H Q V A D M
763 aaaacaatccataagttgactt**tga**AGAAGCCATCTCGCTCGTT 805

```
    K  T  I  H  K  L  T   **
806 GTATCGTCTTTTTTTCTTATAGATAATATTGCAAGGAATAAAAG  849
850 TTGGTTACATTGTTAGAAAAAAAAAAAAAAAAAAAAAAAAAAAAA 894
```

(h) *Geranyl diphosphate synthase* (*AeGDPS*)

```
                                            1 ATCAAATC   8
   9 AACAAAATGAAACAAGAACTTTATTGAAGTATATTTTGCATGATA  53
  54 GATTAGAGAACCAAGAACTTGTTTGCTCTGCATCAAACAGTTTAG  98
  99 TGCAGTAGAGAGGGATTCAAGAAACTTGCAATCTTTCTAGCTAAT  143
 144 atgcataatctacgagcacatgtaaagaaatggatccaattaagt 188
     *M  H  N  L  R  A  H  V  K  K  W  I  Q  L  S
 189 tcaatatttcttgttcaagcaaatcaatttacatgtcttcaaga 233
     S  I  F  S  C  S  S  K  S  I  Y  M  S  S  R
 324 gtcaatgtttttgaaatagaagaagaacccagatccagatttgat 278
     V  N  V  F  E  I  E  E  E  P  R  S  R  F  D
 279 ttcaagtcttatatgataaacaagattacttccatcaatgaagca 323
     F  K  S  Y  M  I  N  K  I  T  S  I  N  E  A
 324 ttggattctgctgttccattgatagagccaatcaagcttcatgaa 368
     L  D  S  A  V  P  L  I  E  P  I  K  L  H  E
 369 gcaatgagatataccttctttctgggggcaaaagggtccgtcct 413
     A  M  R  Y  T  L  L  S  G  G  K  R  V  R  P
 414 attgtttgtattgctgcttgtgagcttgttggaggccatgaatca 458
     I  V  C  I  A  A  C  E  L  V  G  G  H  E  S
 459 acagtgatgccaacagcctgtgctcaggaaatgatacattccatg 503
     T  V  M  P  T  A  C  A  Q  E  M  I  H  S  M
 504 tctgtgatgttggatgatcttccttgtatggacaacgatgatttt 548
     S  V  M  L  D  D  L  P  C  M  D  N  D  D  F
 549 cgccgaggaaaactatcgaatcatagagtttatggagagaagatt 593
     R  R  G  K  L  S  N  H  R  V  Y  G  E  K  I
 594 actcttttagctgttagatcacttcaggctttagcagttgaccat 638
     T  L  L  A  V  R  S  L  Q  A  L  A  V  D  H
 639 gttgttacagctactagaggggttccacctgaaagattagttaga 683
     V  V  T  A  T  R  G  V  P  P  E  R  L  V  R
 684 gctctaactgaaatggcgatactaacagggtcgaaaggtgctgct 728
     A  L  T  E  M  A  I  L  T  G  S  K  G  A  A
 729 gctggacaaattgctgatttgtgcagctctggggaggattttaat 773
     A  G  Q  I  A  D  L  C  S  S  G  E  D  F  N
 774 gtttcgattgagcaattggaacatatacatatgcagaaaacgggc 818
     V  S  I  E  Q  L  E  H  I  H  M  Q  K  T  G
 819 actctatgtgaaggttctgttgtttccggggcaattataggggt 863
     T  L  C  E  G  S  V  V  S  G  A  I  I  G  G
 864 gcgtctgaagaggaaattgagaaacttaggaagttctcgaagtgc 908
     A  S  E  E  E  I  E  K  L  R  K  F  S  K  C
 909 ataggactaatgtttcagattgttgatgatgttcttgatgttact 953
     I  G  L  M  F  Q  I  V  D  D  V  L  D  V  T
 954 aaatcgtcaatggagcttgggaagcccgcggggaaggatgtggta 998
     K  S  S  M  E  L  G  K  P  A  G  K  D  V  V
 999 gccgcgaaggcaacttatccgagattgattgggattgagaagtcg 1043
     A  A  K  A  T  Y  P  R  L  I  G  I  E  K  S
1044 agggaattggctttgaagctgaattctgaggctaaggaacagctt 1088
     R  E  L  A  L  K  L  N  S  E  A  K  E  Q  L
1089 tctggttttgatcaagaaaaggcagctcctctgattgcattggcc 1133
     S  G  F  D  Q  E  K  A  A  P  L  I  A  L  A
1134 gattacatagttgctaggcagaattagGCTTATTCGCTCCCCTTC 1178
      D  Y  I  V  A  R  Q  N  **
1179 CATTTGATTAGGAAAGAATTGTTCATAGTTCACTGATAGGACTATG 1224
1225 TTTTGGGGATTAAACACCGAAACCTTATAGATTGAGATTGAGCTTA 1270
```

1271 GTTGATATACTTTGCTTGTTCGATTATTCAAAAATGATAGAAAGCA 1316
1317 ATGAACCAATGATGGCAATTCCATTTGGCCTGTGTTGATTATTGGA 1362
1363 TTCACCTTTGCATTGGACCTTCAAGAACAGTCACGAAAAATGCTAG 1408
1409 AATCTCTTTTGCGAAAGAAATTGGAATGATATAGGAATGTTCAAAT 1454
1455 TCTAAAAAAAAAAAAAAAAAAAAAAAAAA 1483

(i) *p-Hydroxybenzoic acid geranyltransferase (AePGT)*

1 ATCACCAAATAGAGCTATCTCTTCATTTCTTC 32
33 TCTTAATATTCATCCTCAGCTTCTTTAAAGTTTAAACATCCCAAA 77
78 CTAAAGGATATTAATACTACTTCCTGCAAGAGTTTTCATTGCATT 122
123 TTCACATTCTTCGAACCACAGCTTGGATATTTTCTAAAAAGTACA 167
168 **atg**acgtccaagcaagcacagcagaagaaaggcaagcaaccatct 212
***M** T S K Q A Q Q K K G K Q P S
213 tggattgagctgtatttgcccaaagaggttcgaccatatgcacac 257
W I E L Y L P K E V R P Y A H
258 cttgcaaggttagacaagcctataggcagctggctactagcttgg 302
L A R L D K P I G S W L L A W
303 ccggctttctggtccgttgcattggttgcagattttggaagtcta 347
P A F W S V A L V A D F G S L
348 ccaaaaatgttagccatatttggatggtgggcagtttggattcga 392
P K M L A I F G W W A V W I R
393 ggtgctggatgtaccatcaacgattactttgatcgcgatttcgat 437
G A G C T I N D Y F D R D F D
438 aagaaagtggaacgtacaaaatctagaccacttgctagtggcgct 482
K K V E R T K S R P L A S G A
483 gtctcaccttcccaaggattgtggtggcttgcatttcagctgttc 527
V S P S Q G L W W L A F Q L F
528 attggcttgggtgttctttaccaattcaacatcttgactcttgca 572
I G L G V L Y Q F N I L T L A
573 ttggctatcttgcatgttccccttgttttgcttatcctctcatg 617
L A I L H V P L V F A Y P L M
618 aaaagaattacctattggcctcaagctttcttggagtaatgatc 662
K R I T Y W P Q A F L G V M I
663 agttggggagctctcttaggctcctctgctcttaaaggaagtgtt 707
S W G A L L G S S A L K G S V
708 gttcccagtatcgcctacccgctttacatttcgagcttttctgg 752
V P S I A Y P L Y I S S F F W
753 actcttgtttatgatactatctatgcacatcaagacaaggtagac 797
T L V Y D T I Y A H Q D K V D
798 gatgcaaaagcagggattaaatccactgctctaagatttggagat 842
D A K A G I K S T A L R F G D
843 gcaaccaagatatggattagttggttcggagtaggatgcattgct 887
A T K I W I S W F G V G C I A
888 gctctagttattgggggctcattgtgaacattgggtttccttat 932
A L V I G G L I V N I G F P Y
933 tatgtatttgtggcaatcgcaactggtcaattggcttggcaaatt 977
Y V F V A I A T G Q L A W Q I
978 gtcacagttgatttatcatctcctatggattgtggtcggaaattc 1022
V T V D L S S P M D C G R K F
1023 gtttctaaccaatggtttggtgctataatcttcaccggaatctta 1067
V S N Q W F G A I I F T G I L
1068 cttggaagattgtttact**tag**ATGTTATGCATTATCAAAATTTG 1111
L G R L F T ******
1112 TTTTGTTTTGATAATAACTTTTACATTCTTTTATTATTGGCCA 1154
1155 **AAATAAAT**ATTTGT TCGAACTTCGAAAAAAAAAAAAAAAAAA 1196
1197 AAAAAAA 1203

(j) *Phenylalanine ammonia lyase (AePAL)*

1 AGGAACTTGCTTTGCTAATACTTCCACACGTACATTCCTCTCTACATT 48
49 CAATTGTCTCTCTTTGTGTTTGTCACATTTCTTCAACTACCCTATAGTT 97
98 GGTTTGCATTTATTTCTTGGATCAAGAAAACATTACTACATACAAACCA 146
147 **atg**gaaaccatagttgaaaaaggaaatggaaaaagtatggagttt 191
***M** E T I V E K G N G K S M E F
192 tgcatgcaagatcccttgaactgggaaatggcagctgagtcaatg 236
C M Q D P L N W E M A A E S M
237 aagggtagccacctagatgaagtgaaacgcatggtggctgagttc 281
K G S H L D E V K R M V A E F
282 aggaaaccggtggtgcagcttgccggcaagacgttgactatcggt 326
R K P V V Q L A G K T L T I G
327 caggtggcgtcgatcgctgcacatgacgacggagtcaaggtggag 371
Q V A S I A A H D D G V K V E
372 ctggcggaagcagccagggaaggtgttaaggcaagtagtgattgg 416
L A E A A R E G V K A S S D W
417 gttatggatagtatgaataagggaacggatagttatggtgtaacc 461
V M D S M N K G T D S Y G V T
462 accggatttggtgctacttctcataggaggactaaacaaggggggt 506
T G F G A T S H R R T K Q G G
507 gcccttcaaaaggaacttattagattcttggatgctggaatattt 551
A L Q K E L I R F L D A G I F
552 ggcaatggaacagaaactagccacacactaccacactcagcaaca 596
G N G T E T S H T L P H S A T
597 agagcagccatgcttgttaggatcaatactctgcttcagggttat 641
R A A M L V R I N T L L Q G Y
642 tcaggcatcaggtttgagatcctggaagccatcaccaagttcctg 686
S G I R F E I L E A I T K F L
687 aacagcaacattactccattcctacccctccgtggcactatcagc 731
N S N I T P F L P L R G T I S
732 tcctctggtgacctcgtgcccctctcctacattgcgggattacta 776
S S G D L V P L S Y I A G L L
777 actggccgccccaactccagggttggacctaaaggagagaagctt 821
T G R P N S R V G P K G E K L
822 aacgcggaagaagccttccgccttgcggggatcagtaacgggttc 866
N A E E A F R L A G I S N G F
867 ttcgagttgcagcctaaggaaggacttgcacttgttaatggaaca 911
F E L Q P K E G L A L V N G T
912 gctgttggttctggaatggcttcaatggttctttatgaagccacc 956
A V G S G M A S M V L Y E A T
957 atttggctgtcctgtctgaagtgatctcagcgattttcgctgag 1001
I L A V L S E V I S A I F A E
1002 gtgatgaatggaaagcctgaattcactgatcatttgacacacaaa 1046
V M N G K P E F T D H L T H K
1047 ttgaaacatcatcctggtcagattgaggctgctgctataatggaa 1091
L K H H P G Q I E A A A I M E
1092 cacattttggatggaagtggatatgttaaggctgctcagaagtta 1136
H I L D G S G Y V K A A Q K L
1137 catgagatggatcctctgcagaagcctaagcaagatcgttatgcc 1181
H E M D P L Q K P K Q D R Y A
1182 ctccgtacatcgcctcaatggctcggtcctcttattgaagtgatt 1226
L R T S P Q W L G P L I E V I
1227 cgttcagctacaaagatgattgagagggaaatcaactctgttaac 1271
R S A T K M I E R E I N S V N
1272 gacaacccgttgattgatgtttcgaggaacaaggccttacacgga 1316
D N P L I D V S R N K A L H G
1317 ggaaacttccagggcacgcctattggtgtagccatggacaacact 1361
G N F Q G T P I G V A M D N T
1362 cgccttgccatcgcctcaattggaaagcttttatttgctcaattt 1406
R L A I A S I G K L L F A Q F
1407 tctgaattggttaatgattactacaacaatgggttgccatcaaat 1451

S E L V N D Y Y N N G L P S N
1452 ttgacaggcagcagaaatccaagcttggattatggttttaaggga 1496
L T G S R N P S L D Y G F K G
1497 gctgaaatcgccatggcttcgtactgttcagaacttcaattcttg 1541
A E I A M A S Y C S E L Q F L
1542 gccaatccagtcaccaaccatgtccagagtgctgagcaacacaac 1586
A N P V T N H V Q S A E Q H N
1587 caagatgtcaactctttgggcttgatatcttcaagaaagacatca 1631
Q D V N S L G L I S S R K T S
1632 gaggctgtcgaaatcttgaagctcatgtcctcatctttcttagtt 1676
E A V E I L K L M S S S F L V
1677 gctctcttccaagctgttgatttgaggcatatagaggagaatgtg 1721
A L F Q A V D L R H I E E N V
1722 agactcgcagtcaagaacacagtcagccaggtcgctaagcggaca 1766
R L A V K N T V S Q V A K R T
1767 ttaaccacaggcgttaatggcgagctccacccatcaagattcagc 1811
L T T G V N G E L H P S R F S
1812 gaaaaggatttgcttctcgtggttgaccgggaatatgtctttgcc 1856
E K D L L L V V D R E Y V F A
1857 tacgcagatgacccttgcctcgccacctacccactgatgcagaag 1901
Y A D D P C L A T Y P L M Q K
1902 ctacgagatgttctcgttggacacgccttagccaacggtgaaaac 1946
L R D V L V G H A L A N G E N
1947 gagaaggatgtgaacacttcaatcttccataagattgccatttc 1991
E K D V N T S I F H K I A I F
1992 gaagacgaattaaaggccattctcccaaaagaggtggagaatgca 2036
E D E L K A I L P K E V E N A
2037 cgagcctcggtcgaaaatggcactccagcaatccttaacaggatt 2081
R A S V E N G T P A I L N R I
2082 gaggaatgcagatcatacccattgtacaagtttgtgagggaagaa 2126
E E C R S Y P L Y K F V R E E
2127 ttgggcactgaatttctgactggtgagaaagtgagatcaccaggt 2171
L G T E F L T G E K V R S P G
2172 gaggaattggacaaagtgttcactgcattgtgtgaaggaaagctt 2216
E E L D K V F T A L C E G K L
2217 gttgatccacttctagcttgtttggaggcttggaatgttgctcct 2261
V D P L L A C L E A W N V A P
2262 cttccaatctgt**taa**ATAAACAGTTTTGTGAACTATTTTATGTAC 2306
L P I C **
2307 TTAACTATTTCTTTTTGTTCTCTTCTTTTTCAATTTTCATACTAA 2351
2352 TTCTTCTGTTGATTTGTTTGGTAAGGTGT 2380

(k) ***Cinnamic acid 4-hydroxylase (AeC4H)***

1 AATACGACTCA 11
12 CTATAGGGCAAGCAGTGGTATCAACGCAGAGTACGCGGGGAACCA 56
57 CAAAACCAAACCCAAAACATCCAACCACCATTTTCCTTATCATCC 101
102 **atg**gatcttctcctcttagagaaggctctactaggcctttcttc 146
***M** D L L L L E K A L L G L F F
147 tcagtcatcattgccattgttatctccaagctcggaggcaaaaaa 191
S V I I A I V I S K L G G K K
192 ttcaagctcccacctggtccaatccctgttccaatctttggcaac 236
F K L P P G P I P V P I F G N
237 tggctccaagttggtgatgatctcaaccacagaaacctcactgag 281
W L Q V G D D L N H R N L T E
282 tatgccaaaaagttcggagaaatcttccttcttcggatgggacaa 326
Y A K K F G E I F L L R M G Q
327 aggaacttagtcgtcgtttcgtcccctgatttggctaaggaagtc 371
R N L V V V S S P D L A K E V

372 ctccacactcagggagttgaattcgggtctcgtactagaaacgtt 416
L H T Q G V E F G S R T R N V
417 gtttttgatatcttcacaggtaaaggacaagacatggtgttcacc 461
V F D I F T G K G Q D M V F T
462 gtctacggagagcactggcgaaaaatgaggaggatcatgacggta 506
V Y G E H W R K M R R I M T V
507 cccttcttcacaaacaaagtcgttcaacaatataggcaagggtgg 551
P F F T N K V V Q Q Y R Q G W
552 gagtttgaggtagagagtgcgatcgaggatgtcaagaagaacccg 596
E F E V E S A I E D V K K N P
597 gaatccgaaaccgttggaattgttttgaggaagaggttgcaactt 641
E S E T V G I V L R K R L Q L
642 atgatgtacaataacatgtttaggattatgtttgataggagattt 686
M M Y N N M F R I M F D R R F
687 gagagtgaggatgatcctctgttcaagaagcttagagctttgaat 731
E S E D D P L F K K L R A L N
732 ggtgagaggagtagattggctcagagctttgactataattatggt 776
G E R S R L A Q S F D Y N Y G
777 gatttcattccgattttgaggcctttcttgagaggatacttgaag 821
D F I P I L R P F L R G Y L K
822 atttgcaaggaggttaaggaaactagattgaagttgttcaaggac 866
I C K E V K E T R L K L F K D
867 tactttgttgatgaaagaaagaagattgctagcacgaagagtaca 911
Y F V D E R K K I A S T K S T
912 accagtaatggactgaaatgtgccattgatcatattcttgaggca 956
T S N G L K C A I D H I L E A
957 caacaaaaggggaaatcaatgaagataatgtactatacattgtg 1001
Q Q K G E I N E D N V L Y I V
1002 gagaacattaatgttgctgcaattgagacaacattgtggtccatt 1046
E N I N V A A I E T T L W S I
1047 gagtggggcattgctgaattggtgaaccaccctcgaatccagaag 1091
E W G I A E L V N H P R I Q K
1092 aagctacgtgatgaaatcgacgcgattcttggacctggcgtacaa 1136
K L R D E I D A I L G P G V Q
1137 gtaaccgagcccgatactcacaagctaccataccttcaagccgtg 1181
V T E P D T H K L P Y L Q A V
1182 gtcaaggagacccttcgtcttcgtatggccattcctcttttggtg 1226
V K E T L R L R M A I P L L V
1227 ccacacatgaacctccatgacgcgaagctcaatggctacgacatt 1271
P H M N L H D A K L N G Y D I
1272 ccagccgagagcaagattttggtaaacgcgtggtggctagcgaac 1316
P A E S K I L V N A W W L A N
1317 aaccccgagcaatggaagaaccctgaagaattcaggcccgagagg 1361
N P E Q W K N P E E F R P E R
1362 ttcttggaagaggaggctaaggttgaggccaatggcaatgacttc 1406
F L E E E A K V E A N G N D F
1407 agataccttccgtttggagtaggaaggagaagctgccctggaata 1451
R Y L P F G V G R R S C P G I
1452 attcttgccttgcctattcttggaatcacattgggacgtttggtc 1496
I L A L P I L G I T L G R L V
1497 cagaactttgagctattgcctcctcctggtcaatctaaacttgat 1541
Q N F E L L P P P G Q S K L D
1542 acttcagagaaaggtggcaattcagtctgcatatattgaagcat 1586
T S E K G G Q F S L H I L K H
1587 tctaccattgtgatgaagccaagagcc**taa**TAATTCTCTTTGTTG 1631
S T I V M K P R A **
1632 GATTTATACACATTATTGTGAACTCTCTAGCTATATAATTCAGTC 1676
1677 GTGGTTGTAATTGTTGAATATTATGATCATTTTTT**AAATAATC**CA 1721
1722 TCTCTCATTATTTCGTTACAAAAAAAAAAAAAAAAAAAAAAAAAA 1666
1767 A

(l) *4-coumaric acid:CoA ligase (Ae4-CL)*

```
       1 GAGGAAACTTGAACATATCCTCAAAGATTCCCCTTTTCT 39
  40 TTGGTTGCCTATCATTTCAATCAAATTTGAACCACCCCCTCCCAA 84
  85 atggacactcaaaccaaaacagaccaaaaagacattatcttcaga 129
     *M  D  T  Q  T  K  T  D  Q  K  D  I  I  F  R
 130 tcaaaactccctgatatatacatcccaagacatctaccattgcat 174
     S  K  L  P  D  I  Y  I  P  R  H  L  P  L  H
 175 tcctattgttttgaaaacatttctcaattcaactcaagaccgtgc 219
     S  Y  C  F  E  N  I  S  Q  F  N  S  R  P  C
 220 ttaatcaatggctcaaatgatagagtatatacatatgcagaagtt 264
     L  I  N  G  S  N  D  R  V  Y  T  Y  A  E  V
 265 gagctaacttcaagaaaagttgcagcagggcttcataaacatggg 309
     E  L  T  S  R  K  V  A  A  G  L  H  K  H  G
 310 atcaaacaaactgaaaccatcatgcttttgctcccaaattgccct 354
     I  K  Q  T  E  T  I  M  L  L  L  P  N  C  P
 355 gaatttgtttttgcatttcttggtgcttcttatataggagcagtc 399
     E  F  V  F  A  F  L  G  A  S  Y  I  G  A  V
 400 tccacaactgccaacccatttttacctcctctgaaatcatcaaa 444
     S  T  T  A  N  P  F  F  T  S  S  E  I  I  K
 445 caagccaaagcatccaaaacaaagctcatcatcacagtagcaagc 489
     Q  A  K  A  S  K  T  K  L  I  I  T  V  A  S
 490 aatgtcccaaaactcaaggaatttctcaagaaaatggtgtcaag 534
     N  V  P  K  L  K  E  F  S  Q  E  N  G  V  K
 535 atcatgtgcattgatgaacaaattgagggttgtcttcatttctca 579
     I  M  C  I  D  E  Q  I  E  G  C  L  H  F  S
 580 ttagacctggaaaacacagatgaaaccaccttgccagaagtcgag 624
     L  D  L  E  N  T  D  E  T  T  L  P  E  V  E
 625 atcctccccaacgacgtcgttgcattgccctattcatcaggaaca 669
     I  L  P  N  D  V  V  A  L  P  Y  S  S  G  T
 670 acagggctaccaaaaggggtgatgctcacacacaaaggactagta 714
     T  G  L  P  K  G  V  M  L  T  H  K  G  L  V
 715 acaagtgttgcacaacaagttgatggtgaaaatgcaaacttgtac 759
     T  S  V  A  Q  Q  V  D  G  E  N  A  N  L  Y
 760 atgcatcaagaagatgttgtgatgtgcactttgccgttgtttcat 804
     M  H  Q  E  D  V  V  M  C  T  L  P  L  F  H
 805 atatattcgatgaattcgattctgctttgtgggttgagagtaggg 849
     I  Y  S  M  N  S  I  L  L  C  G  L  R  V  G
 850 gctgcaatattgttgatgcacaagtttgatattgcgccatttctt 894
     A  A  I  L  L  M  H  K  F  D  I  A  P  F  L
 895 gaattgatacagaggtataaggtgacaattgggccatttgttcca 939
     E  L  I  Q  R  Y  K  V  T  I  G  P  F  V  P
 940 ccaattgtgttggccattgctaagagcaatgtagttgatcagttt 984
     P  I  V  L  A  I  A  K  S  N  V  V  D  Q  F
 985 gatctgtcgtcggtgaggaccgtcatgtccggggcggcaccactg 1029
     D  L  S  S  V  R  T  V  M  S  G  A  A  P  L
1030 gggaaggagcttgaagatgctgttagagctaagttccctaatgcg 1074
     G  K  E  L  E  D  A  V  R  A  K  F  P  N  A
1075 aaacttggtcagggatatgggatgacagaagcagggccggtgttg 1119
     K  L  G  Q  G  Y  G  M  T  E  A  G  P  V  L
1120 gctatgtgtctggcttttgcaaaagaaccatttgagatcaaatca 1164
     A  M  C  L  A  F  A  K  E  P  F  E  I  K  S
1165 ggagcatgtggtacagtcgttagaaatgcagagatgaaaatcata 1209
     G  A  C  G  T  V  V  R  N  A  E  M  K  I  I
1210 gataccgaaactggtgtctctttgcctcgtaaccaatccggtgaa 1254
     D  T  E  T  G  V  S  L  P  R  N  Q  S  G  E
1255 atttgcattcgtggagaccaaatcatgaaaggttatctgaatgat 1299
     I  C  I  R  G  D  Q  I  M  K  G  Y  L  N  D
1300 cccgaggcgacggagagaacaatagacaatgaagggtggttacat 1344
```

P E A T E R T I D N E G W L H
1345 actggtgatattgggtacattgacgatgatgatgagcttttcatt 1389
T G D I G Y I D D D D E L F I
1390 gtggatcgtttgaaggagcttattaaatacaaggggttccaagtg 1434
V D R L K E L I K Y K G F Q V
1435 gctcctgctgaacttgaagctcttcttgttactcaccccaaagtc 1479
A P A E L E A L L V T H P K V
1480 tctgatgctgccgttgtttcaatgaaagatgaggggcaggagag 1524
S D A A V V S M K D E G A G E
1525 gtgccagttgcatttgtcgtgaggtcaaatggttccacaattact 1569
V P V A F V V R S N G S T I T
1570 gaggatgagatcaagcaattcgtatctaaacaggtgattttctac 1614
E D E I K Q F V S K Q V I F Y
1615 aagagaataaaccgtgtgttctttgtggattcgattcccaaatct 1659
K R I N R V F F V D S I P K S
1660 ccatctggaaagattttgaggaaggacttgagagctaagttggct 1704
P S G K I L R K D L R A K L A
1705 gctggcttcctcaatggtcctactactacaactaatggggtgcca 1749
A G F L N G P T T T T N G V P
1750 aattctggtgtagctaaagatgtgccaaacggggtttccaacggt 1794
N S G V A K D V P N G V S N G
1795 gtttctaaggccaatggcggtggcatcgaagaagggattgctaat 1839
V S K A N G G G I E E G I A N
1840 ggagttgcgaaggccaatggtggcgttgccaaagaagagattgct 1884
G V A K A N G G V A K E E I A
1885 aatggagtttcgaaggccaattgtggtgttgccaaggaagagatt 1929
N G V S K A N C G V A K E E I
1930 gctaacggaataccaactagcgtcgccaatggtgtagctacaaaa 1974
A N G I P T S V A N G V A T K
1975 ggagtttataaacaaaagataaatggtgttgtttcaaatggagtg 2019
G V Y K Q K I N G V V S N G V
2020 gaaaatggtgtggtgtcaaatgggacaacaaatggtgtacacaat 2064
E N G V V S N G T T N G V H N
2065 **tag**GGTATT**ATATAAAA**AAATCAATATCAAAAAAAAAAAAAAA
**
2107 AAAAAAAAAAAAAA 2121
