# Supplementary material for: Expression of 3-hydroxy-3-methylglutaryl-CoA reductase, p-hydroxybenzoate-m-geranyltransferase and genes of phenylpropanoid pathway exhibits positive correlation with shikonins content in arnebia [Arnebia euchroma (Royle) Johnston]
Source: BMC Mol Biol. 2010 Nov 21;11:88. doi: 10.1186/1471-2199-11-88 (PMC3002352; doi:10.1186/1471-2199-11-88)
Supplement: Additional file 4 — Sequence alignment (ClustalW2; http://www.ebi.ac.uk/) of the deduced amino acid sequences of arnebia cDNAs. Sequence alignment (ClustalW2; http://www.ebi.ac.uk/) of the deduced amino acid sequences of arnebia cDNAs with the respective reported sequences from other plants: (a) AeACTH (GenBank:AAU95618.1Nicotiana tabacum, GenBank:ABC74567.1Picrorhiza kurrooa, GenBank:AAM00280.1Arabidopsis thaliana, GenBank:AAL18924.1Hevea brasiliensis); (b) AeHMGS (GenBank:ABX55778.1Solanum lycopersicum, GenBank:ACD87446.1Camptotheca acuminate, GenBank:EEF51079.1Ricinus communis, GenBank:AAS46245.1Hevea brasiliensis, GenBank:AAG32923.1Brassica juncea); (c) AeHMGR (GenBank:AAL54878.1Nicotiana tabacum, GenBank:BAA93631.1Solanum tuberosum, GenBank:ABC74565.1Picrorhiza kurrooa, GenBank:ABV25901.1Antirrhinum majus, GenBank:AAD38873.1Oryza sativa, GenBank:AAA33040.1Camptotheca acuminata); (d) AeMVK (GenBank:AAN72115.1Arabidopsis thaliana, GenBank:ABD32397.1Medicago truncatula, GenBank:ABV02026.1Nicotiana langsdorffii x Nicotiana sanderae, GenBank:ACG46416.1Zea mays, GenBank:AAL31086.1Oryza sativa); (e) AePMVK (GenBank:BAD44652.1Arabidopsis thaliana, GenBank:AAL18926.1Hevea brasiliensis, GenBank:CAO63313.1Vitis vinifera, GenBank:ABF95008.1Oryza sativa, GenBank:NP_001149345.1Zea mays); (f) AeMVDD (GenBank:BAF98285.1Hevea brasiliensis, GenBank:XP_002521172.1Ricinus communis, GenBank:NP_566995.1Arabidopsis thaliana, GenBank:ABV02028.1Nicotiana langsdorffii x Nicotiana sanderae, GenBank:ABW87316.1Solanum lycopersicum); (g) AeIPPI (GenBank:BAB40974.1Nicotiana tabacum, GenBank:ABX55779.1Solanum lycopersicum, GenBank:AAQ84167.1Pueraria montana var. lobata, GenBank:AAB94132.1Camptotheca acuminate, GenBank:AAF29973.1Adonis palaestina); (h) AeGDPS (GenBank:AAS82860.1Antirrhinum majus, GenBank:AAF08793.1Mentha x piperita, GenBank:AAW66658.1Picrorhiza kurrooa, GenBank:ACQ90682.1Humulus lupulus, GenBank:AAN01133.1Abies grandis, GenBank:ACA21458.1Picea abies); (i) AePGT (GenBank:NP_849431.1Arabidopsis tha [file 1471-2199-11-88-S4.PDF]

Additional file 4: Supplementary Figure S2.

(a) Acetoacetyl-CoA thiolase (AeACTH)

```
Nicotiana        MAPAAADSIKPRDVCIVGVARTPMGGFLGSLSSLSATKLGSIAIEGALKKANVDPSLVEE 60
Picrorhiza       MAPAAADSIKPRDVCIVGVSRTPMGGFLGSISSLPATKLGSIAIESALKRANVDPSHVQE 60
Arabidopsis      -MAHTSESVNPRDVCIVGVARTPMGGFLGSLSSLPATKLGSLAIAAALKRANVDPALVQE 59
Hevea            -MSPSSDSINPRDVCIVGVARTPMGGFLGSLSSFSATKLGSIAIQAALKRANVDPSLVQE 59
Arnebia          ---MVEDSIKPRDVCIVGVARTPMGGFLGSLSSLSATELGSIAIKSALKRANVDPSLVQE 57
                       :*::*********:**********:**:.**:***:** .***:*****: *:*

Nicotiana        VFFGNVLSANLGQAPARQAALGAGIPNTVVCTAVNKVCASGLKATMLAAQSIQLGINDVV 120
Picrorhiza       VFFGNVLSANLGQAPARQAALGAGIPNTVICTTVNKVCASGMKAAMLAAQSIQLGINDVV 120
Arabidopsis      VVFGNVLSANLGQAPARQAALGAGIPNSVICTTVNKVCASGMKAVMIAAQSIQLGINDVV 119
Hevea            VFFGNVLSANLGQAPARQAALGAGIPNSVICTTINKVCASGMKATMLAALTIQVGINDIV 119
Arnebia          VIFGNVLSANLGQAPARQAALGAGLPNTVSCTTVNKVCASGMKATMLAAQTIQLGINDVV 117
                 *.**********************:**:* **::*******:**.*:** :**:****:*

Nicotiana        VAGGMESMSNVPKYIAEARKGSRLGHDSLVDGMLKDGLTDVYKDCGMGVCAEICAENHKI 180
Picrorhiza       VAGGMESMSNVPKYLAEARKGSRLGHDSLVDGMLKDGLWDVYNDYGMGVCAELCAEHHSV 180
Arabidopsis      VAGGMESMSNTPKYLAEARKGSRFGHDSLVDGMLKDGLWDVYNDCGMGSCAELCAEKFQI 179
Hevea            VAGGMESMSNAPKYLAEARRGSRLGHDTIIDGMLKDGLWDVYNDFGMGVCAEICADQHNI 179
Arnebia          VAGGMGSMSNCPKYLAEARRGSRLGHDSLVDGMLKDGLWDVYNDVGMGVCAETCAENHKI 177
                 ***** **** ***:****:***:***:::******** ***:* *** *** **::..:

Nicotiana        TREEQDDYAVQS-------FERGIAAQEAGAFAWEIVPVEVPGGRGKPSIIVDKDDGLGK 233
Picrorhiza       TREEQDNYAVQS-------FERGIAAKDSGAFSWEIVPVEVSGGRGGPSTIVDKDEGLGK 233
Arabidopsis      TREQQDDYAVQS-------FERGIAAQEAGAFTWEIVPVEVSGGRGRPSTIVDKDEGLGK 232
Hevea            TREEKDSYAIRS-------FERGNSAQNGGVFSWEIVPVEVSGGRGKSVMVVDKDEGLIK 232
Arnebia          SREDQDNFARRPGFICNSKLERGRAAQNSGAFSWEIVPVELSGSRGKPTVIIDKDEGLSK 237
                 :**::*.:* :.       :*** :*::.*.*:*******:.*.** .  ::***:** *

Nicotiana        FDGAKLRKLRPSFKEKDGTVTAGNASSISDGAAALVLVSGEKAIKLGLNVIAKISGYADA 293
Picrorhiza       FDAAKLRKLRPSFKETGGSVTAGNASSISDGAAALVLVSGEKALKLRLEVIAKITGYADA 293
Arabidopsis      FDAAKLRKLRPSFKENGGTVTAGNASSISDGAAALVLVSGEKALQLGLLVLAKIKGYGDA 292
Hevea            FDAAKLRKLRP--ISRIGSVTAGNASIISDGAAALVLVSGEKAIELGLQVIARIRGYGDA 290
Arnebia          FDASRLRKLRPTFKPDGGSVTAGNASSISDGAAALVLVSGEKAIFFGLHVIAKIRGFADA 297
                 **.::******      *:******* ****************: : * *:*:* *:.**

Nicotiana        AQAPELFTTAPALAIPKAIKSASLEASQIDYYEINEAFAVVSLANQKLLGLNPEKVNVHG 353
Picrorhiza       AQAPELFTTAPAIAIPKAISNAGLEASQIDYYEINEAFAVVALANQKLLSLNPEKVNVHG 353
Arabidopsis      AQEPEFFTTAPALAIPKAIAHAGLESSQVDYYEINEAFAVVALANQKLLGIAPEKVNVNG 352
Hevea            AQAPELFTTAPALAIPKAISNAGLEASQIDYYEINEAFSVVALANQKILGLNPEKLNVHG 350
Arnebia          AQAPELFTTAPALAIPKAIKNSGLVASQIDYYEINEAFSVVALVNQRLLNLKSEQLNLHG 357
                 ** **:******:******  :.* :**:*********:**:*.**::*.: .*::*::*

Nicotiana        GAVSLGHPLGCSGARILVTLLGVLRQKNGKYGAAGVCNGGGGASALVLELV--------- 404
Picrorhiza       GAVSLGHPLGCSGARILVTLLGVLRQKGGKYGVGGVCNGGGGASAVVIELL--------- 404
Arabidopsis      GAVSLGHPLGCSGARILITLLGILKKRNGKYGVGGVCNGGGGASALVLELL--------- 403
Hevea            GAVSLGHPLGCSGARILVTLLGVLRHKNGKYGVASICNGGGGASALVLELMSVGRVGRSL 410
Arnebia          GAVSLGHPLGCSDARILVTLLGVLRHKNGKFGVAGVCNGGGGASAVVLEFMSTERMGVRS 417
                 ************.****:****:*:::.**:*...:*********:*:*::

Nicotiana        --
Picrorhiza       --
Arabidopsis      --
Hevea            L- 411
Arnebia          KL 419
```

(b) 3-hydroxy-3-methylglutaryl-CoA synthase (AeHMGS)

```
Solanum          MAAQPKNVGILAVEIYFPPTCLQQEALEAHDGASKGKYTIGLGQDCMGFCTEVEDVISMS 60
Camptotheca      MASEQKNVGILAMEIYFPSLFVQQEAMEVHDGASKGKYTIGLGQDCMAFCTEVEDVISMG 60
Ricinus          ---MAKNVGILALDIYFPPTCVQQEALEAHDGASKGKYTIGLGQDCMGFCTEVEDVISMS 57
Hevea            ---MAKNVGILAMDIYFPPTYVQQEALEAHDGASKGKYTIGLGQDCMAFCTEVEDVISMS 57
Brassica         ---MAENVGILAMDIYFPPTCVQQEALEAHDGASKGKYTIGLGQDCLAFCTELEDVISMS 57
Arnebia          ------------------------------------------------------------

Solanum          LTAVTSLLEKYAIDPKQIGRLEVGSETVIDKSKSIKTFLMQIFEKSGNTDIEGVDSTNAC 120
Camptotheca      LTAVTSLLTKYGIDPKQIGRLEVGSETVIDKSKSTKTFLMQIFEECGNTDIEGVDSTNAC 120
Ricinus          LTAVTSLLDNYNIDPKQIGRLEVGSETVIDKSKSIKTFLMQIFEKHGNTDIEGVDSSNAC 117
```

```
Hevea            LTAVTSLLEKYNIDPNQIGRLEVGSETVIDKSKSIKTFLMQIFEKFGNTDIEGVDSANAC 117
Brassica         FNAVTSLLDKYKIDPMQIGRLEVGSETVIDKSKSIKTFLMQLFEKCGNTDVEGVDSTNAC 117
Arnebia          ------------------------------------------------------------

Solanum          YGGTAALFNCVNWVESSSWDGRYGLVVCTDSAVYAEGAARPTGGAAAIAMLVGPDAPIVF 180
Camptotheca      YGGTAALFNCVNWVESSSWDGRYGLVVCTDSAVYAEGPARPTGGAAAVAMLVGPDAPIAF 180
Ricinus          YGGTAALFNCVNWVESSSWDGRYGLVVCTDSAVYAEGPARPTGGAAAIAMLIGPDAPIAF 177
Hevea            YGGTAALFNCVNWVESNSWDGRYGLVVCTDSAVYAEGPARPTGGAAAIAMLIGPEAPIAF 177
Brassica         YGGTAALLNCVNWVESNSWDGRYGLVICTDSAVYAEGPARPTGGAAAIAMLIGPNAPIVF 177
Arnebia          --------------------------ICTDSAVYADGPARPTGGAAAVAMLIGPDAPIAF 34
                                           :********:*.*********:***:**:***.*

Solanum          ESKIRASHMAHVYDFYKPILDSEYPVVDGKLSQTCYLMALDSCYKSLCNKYEKLEGKQFS 240
Camptotheca      ESKFRGSHMSHAYDFYKPNLASEYPVVDGKLSQTCYLMALDSCYKRYCHKYERLEGKQFS 240
Ricinus          ESKYRGSHMSHAYDFYKPNLASEYPVVDGKLSQTCYLMALDSCYKHFCAKYEKFEGKQFS 237
Hevea            ESKFRGSHMSHAYDFYKPNLASEYPVVDGKLSQTCYLMALDSCYKHFCAKYEKLEGKQFS 237
Brassica         ESKLRGSHMAHVYDFYKPNLASEYPVVDGKLSQTCYLMALDSCYKHLCNKFEKLEGKEFS 237
Arnebia          ESKLRGSHMAHVYDFYKPNLASEYPVVDGKLSQTCYLMALDSCYKTLCNKYKKLEGKQFS 94
                 *** *.***:*.****** * ************************  * *::::***:**

Solanum          IDDAAYFVFHSPYNKLVQKSTARLMFNDFTRNASSIDESTKEKLAPFSSLTGDESYQSRD 300
Camptotheca      IADADYFVFHSPYNKLVQKSFGRLLFSDFLRNVSSIDEATKEKLGPFSTLTVDESYQSRD 300
Ricinus          ISDAEYFVFHSPYNKLVQKSFARLVFNDFVRNASSIDEAAKEKLAPFSTLTGDESYQNRD 297
Hevea            ISDAEYFVFHSPYNKLVQKSFARLVFNDFVRNASSIDDAAKEKLAPFSTLSGDESYQNRD 297
Brassica         INDADYFVFHSPYNKLVQKSFARLLYNDFLRNASSIDEAAKEKFIPYSSLSLDESYQSRD 297
Arnebia          ISDADYFVFHSPYNKLVHKSFARLLFNDFVNNASSIDEAAKEKFAPFASLTGDESYASRD 154
                 * ** ************:** .**::.** .*.****:::***: *:::*: **** .**

Solanum          LEKTSWQVAKPFYDEKVKPATLIPKQVGNMYTASLYAAFASLLHNKHSSLAGQRVVLFSY 360
Camptotheca      LEKATQQLAKPLYDAKVQPATLIPKQVGNMYTASLYAAFASLIHNKHSTLVGQRVVMFSY 360
Ricinus          LEKVSQQVAKPLYDAKVQPTTLIPKQVGNMYTASLYAAFASLLHNKNSKLAGKRVILFSY 357
Hevea            LEKVSQQVAKPLYDAKVQPTTLIPKQVGNMYTASLYAAFASLLHNKHTELAGKRVILFSY 357
Brassica         LEKVSQQVAKTFYDGKVQPTTLVPKEVGNMYTASLYAAFASLIHNKHSDLAGKRVVMFSY 357
Arnebia          LEKVAQQVAKPFYDTKVQPATLIPKQVGNMYTASLYAAFASLLHNKNSSLDGKRVMMFSY 214
                 ***.: *:**.:** **:*:**:**:****************:***:: * *:**::***

Solanum          GSGLTATMFSLKLHEGQHPFNLSNIATVMNIEEKLKSRHEFTPENFIETMKIMEHRYGGK 420
Camptotheca      GSGLTATMFSFRIRDCEHPFSLKNIATVMNVADKLKSRHEFPPENFVETMKLMEHRYGAK 420
Ricinus          GSGLTATMFSLRLHEGQHPFSLSNIATVMNVTDKLKSRHEFSPEKFVETMKLMEHRYGAK 417
Hevea            GSGLTATMFSLRLHEGQHPFSLSNIATVMNVAGKLKTRHEFPPEKFAVIMKLMEHRYGAK 417
Brassica         GSGSTATMFSLRLCENKSPFSLSNIASVMDIGGKLKARHEYAPEKFVETMKLMEHRYGAK 417
Arnebia          GSDSTATMFSLRLREGQQPFSLSNIATVMNVQRSLSQGMSCLQKKFVDLMQLMEHRYGGK 274
                 **. ******::: : : **.*.***:**::  .*.   .   ::*   *::******.*

Solanum          DFVTSKD--CSLLAPGTYYLTEVDSKYRRFYAKKC--------AENG-LANGH 462
Camptotheca      DFVTSKD--CSLLSPGTYYLTEVDSMYRRFYAKKAGDNITGKTCENGSLSNGH 471
Ricinus          DFVTSQD--CSLLAPGTYYLTEVDTMYRRFYAKKLVDN----TCENGLIADGH 464
Hevea            DFVTSKD--CSILAPGTYYLTEVDTMYRRFYAQKAVGD----TVENGLLANGH 464
Brassica         DFVTSKEGILELLAPGTYYLKEVDSLYRRFYGKKG---------DDGSIANGH 461
Arnebia          DFVTSKD--CSLLAPGTYYLTEVDSKYRRFYSKKE--------SENGKLANGH 317
                 *****::    .:*:******.***: *****.:*          ::* :::**
```

(c) 3-hydroxy-3-methylglutaryl-CoA reductase (AeHMGR)

```
Nicotiana        MDVRRRSEKPAYPTKEF----AAGEKPLKPHKQQQQQDNSLLIASDALPLPLYLTNGLFF 56
Solanum          MDVRRRPVKPLYPSEHI----SSGE-PLKPHNQ-----DSSVKASDALPLPLYLTNGLFF 50
Picrorhiza       MDFRRRPPRPSQIKHQL----QSPASGASADHHNQ---SSSPKASDALPLPLYLTNGIFF 53
Antirrhinum      MDDIRR----------------RRATTKSCNSGDV---VSWAKASDALPVPLYLTNGIFF 41
Oryza            MEVRRR-------------------APLPPPPG-------RVQAGDALPLPIRHTNLIFS 34
Arnebia          MEYS-KSMN----------------KDTTSLKKQNVELDFSRKASEAFSLPLRFANAIFF 43
Camptotheca      MDVRRRSINSIHQIPSVGGTAPPMLKPKQPTKVDAVDLPDSPKASDALPLPLYITNGVFF 60
                 *:   :                                     *.:*:.:*:  :* :*

Nicotiana        TMFFSVMYYLLSRWREKIRNSTPLHVVTFSELVAIVSLIASVIYLLGFFGIGFVQSFVSR 116
Solanum          TMFFSVMYFLLHRWREKIRNGIPLHVLNFSELVAMVSLIASVIYLLGFFGIGFVQSFVSK 110
Picrorhiza       TLFFSVAYFLLHRWRDKIRNSIPLHVLTVSELAAIICLIASFIYLLGFFGIDFVQSFTSK 113
Antirrhinum      CLFFSVAYFLLHQWRDKIRNSIPLHVLTLSELAAIICLIASFIYLLGFFGIDFVQSFISK 101
Oryza            ALFAASLAYLMRRWREKIRSSTPLHVVGLAEMLAIFGLVASLIYLLSFFGIAFVQSIVSS 94
Arnebia          PLFFSVVYYLLMRWREKIRNSTPLHVVSFHEILAIVALVASSIYLLGFLSIGLVQ----L 99
Camptotheca      TLFFTVVYYLLVRWREKIRNSTPLHVVTLSEIAAIFTFVASFIYLLGFFGIGLVQPFTSR 120
                  :* :   :*: :**:***.. ****: . *: *:. ::** ****.*:.* :**
```

```
Nicotiana        DNNDDSWDVEDENDEQFLLEEDSRRGPATTLGCTAVPPPPARQVVPMVPPQPAKVAAMSE 176
Solanum          GNND-SWDVEDESPEQFIDR--------------TVTPPPVRRNIPMKS------VPVAE 149
Picrorhiza       SENEDGFILHQD---------------------RSIHCPPIA------------------ 134
Antirrhinum      SESDEDPQPIGD---------------------IHV-VPPVQ------------------ 121
Oryza            SDDEEEDFLVGPARGSS----------------AAAAVAPRAPSSPAQCS------LLGS 132
Arnebia          SGEDEEEEEEEVPLYDTKDVN-------KDIQVDRVTLMEEKCHVACIS----------T 142
Camptotheca      SSHDDVWGVDDDEDVDEIVLK-------EDTRTVPCAAAPVDCPLPPIKP--------KV 165
                 .  :

Nicotiana        KPAPLVTPAASEEDEEIIKSVVQGKMPSYSLESKLGDCKRAASIRKEALQRITGKSLEGL 236
Solanum          KTAQIITPFSSEDDEVVIKSVVEGRIPSYSLESKLGDCKRAAFIRKEALQRSSGKSLEGL 209
Picrorhiza       -PKPIVKMDLPQDDEEVVMSVVSGEVPSYSLESRLGDCFRAAKIRREALQRVTGRSVEGL 193
Antirrhinum      -KEKEMEMEMDVVDSEMVRRVVCGEIPSYSLESKLGDCLKAAKIRREAVQQVMGRSLEGL 180
Oryza            PHDDAARERMPEEDEEIVSSVVAGKVPSYVLETKLGDCRRAAGIRREAVRRITGRQIEGL 192
Arnebia          ASPKPMAKYCPEEDEEIVQSVVQGNTPSYSLESKLGDCFRAAAIRREALQRLTGKSLEGL 202
Camptotheca      VDPVPISPPSSEEDEEIIKSVVEGTTPSYALESKLGDSHRAAAIRREALQRMTKKSLAGL 225
                              *. ::  ** *  *** **::***. :** **:**:::   :.: **

Nicotiana        PLEGFDYESILGQCCEMPIGYVQIPVGIAGPLLLDGREYSVPMATTEGCLVASTNRGCKA 296
Solanum          PLDGFDYESILGQCCEMPIGYIQIPVGIAGPLLLNGKEFSVPMATTEGCLVASTNRGCKA 269
Picrorhiza       PLEGFDYESILGQCCEMPVGYAQIPVGIAGPLLLNGCEYSVPMATTEGCLVASTNRGCKA 253
Antirrhinum      PLERFDYDSILGQCCEMPVGYVQIPVGIAGPLLLNGGEYSVPMATTEGCLVASTNRGCKA 240
Oryza            PLDGFDYASILGQCCELPVGYVQLPVGIAGPLLLDGQRFYVPMATTEGCLVASTNRGCKA 252
Arnebia          PLEHFDYDSILGQCCEMPIGYVQLPVGIAGPLLLDDKEYSVPMATTEGCLVASTNRGCKA 262
Camptotheca      PLDGFDYDSILGQCCEMPVGYVQIPVGIAGPLLLDGREYSVPMATTEGCLVASTNRGCKA 285
                 **: *** ********:*:** *:**********:. .: ********************

Nicotiana        IYASGGATSVLLRDGMTRAPCVRFGTAKRAAELKFFVEDPVKFETLAAVFNQSSRFARLQ 356
Solanum          IYVSGGATSVLFRDAMTRAPVVRFGSAKRAAELKFFVEDPMNFETLSVVFNKSSRFARLQ 329
Picrorhiza       IYASGGATCVLLRDAMTRAPVVRFSSVKRATDLKFFLEDPLNFDTLSVVFNKSSRFARLQ 313
Antirrhinum      IYVSGGATCVLLRDGMTRAPVVRFSSAKRATDLKFFLENPLNFDTLSLVFNKSSRFARLQ 300
Oryza            IAESGGAVSVVLRDGMTRAPVARLPTARRAAELKAFLEDSVNFNTLSMVFNRSSRFARLQ 312
Arnebia          IYASGGATSVVLRDGMTRAPVVRFGTAKRAAELKFFLEDPLNFETLSAVFNQSSRFGRLQ 322
Camptotheca      IFACGGATSVLLRDAMTRAPVVRFGSAKRAADLKFFLENPLNFETLAAVFNSSSRFGKLQ 345
                 *  .***..*::**.***** .*: :.:**::** *:*:.::*:**: *** ****.:**

Nicotiana        RIQCAIAGKNLYMRFVCSTGDAMGMNMVSKGVQNVLDYLQNEYPDMDVIGISGNFCSDKK 416
Solanum          NIQCAIAGKNLYMRFSCSTGDAMGMNMVSKGVQNVLDYLQNEYPDMDIIGISGNYCSDKK 389
Picrorhiza       SIQCGIAGKNLYIRFSCSTGDAMGMNMVSKGVQNVLDFLQNDFPDMDVIGISGNFCSDKK 373
Antirrhinum      SIQCAIAGKNLYIGFTCSTGDAMGMNMVSKGVQNVLDFLQSDFPDMDVIGCSGNFCSDKK 360
Oryza            GVQCAMAGRNLYMRFSCCTGDAMGMNMVSKGEQNVLDYLQDDFPDMDVISISGNFCSDKK 372
Arnebia          SIKCAIAGKNLYMRFSCSTGDAMGMNMVSKGTQHVIEFLEKEFPDMDVIGISGNYCSDKK 382
Camptotheca      NIKCAIAGKNLYMRYSCSTGDAMGMNMISKGVQNVLDFLQDDFPDMDVIGISGNYCSDKK 405
                  ::*.:**:***: : *.*********:*** *:*:::*:.::****:*. ***:*****

Nicotiana        PAAVNWIEGRGKSVVCEAIITEEVVKKVLKTEVAALVEPNMLKNLTGSAMAGALGGFNAH 476
Solanum          PAAVNWIEGRGKSVVCEAIIKEDVVKKVLKTEVATLVELNMLKNLTGSAMAGALGGFNAH 449
Picrorhiza       PAAVNWIEGRGKSVVCEAVISGDIVTKVLKTNVASLVELNMLKNLAGSAIAGALGGFNAH 433
Antirrhinum      PAAVNWIEGRGKSVVCEAVITEDVVTKVLKTTVPALVELNMLKNLTGSAIAGALGGFNAH 420
Oryza            PAAVNWIEGRGKSVVCEAVIKEDVVKKVLKTNVQSLVELNVIKNLAGSAVAGALGGFNAH 432
Arnebia          PAAVNWIEGRGKSVVCEAII-EDVVKKVLKT-MCSCGALHAQELLS---QWRGLWWINAH 437
Camptotheca      PAAVNWIEGRGKSVVCEAVIKEEVVKKVLKTNVASLVELNMLKNLTGSAMAGALGGFNAH 465
                 ******************:*  ::*.***** : :    :  : *:      .*  :***

Nicotiana        ASNIVSAVFIATGQDPAQNIESSHCITMMEAVNDGKDLHVSVTMPSIEVGTVGGGTQLAS 536
Solanum          ASNIVSAVYLATGQDPAQNIESSHCITMMEAVNDGKDLHISVTMPSIEVGTVGGGTQLAS 509
Picrorhiza       AANIVSAIFIATGQDPAQNIESSHCITMMEAVNDGKDLHVSVTMPSIEVGTVGGGTQLAS 493
Antirrhinum      AANIVSAIFIATGQDPAQNIESSHCITMMEAVNDGKDLHVSVTMPSIEVGTVGGGTQLAS 480
Oryza            ASNIVTAIFIATGQDPAQNVESSHCITMLEAVNDGRDLHISVTMPSIEVGTVGGGTQLAS 492
Arnebia          ASNIVSAIFIATGQDPAQNIESSHCITMMEAVNEGKDLHVSVTMPSIEVGTVGGGTQLAS 497
Camptotheca      ASNIVSAVYLATGQDPAQNVESSHCITMMEAINDGKDLHVSVTMPSIEVGTVGGGTQLAS 525
                 *:***:*:::*********:********:**:*:*:***:********************

Nicotiana        QSACLNLLGVKGANREAPGSNARLLATIVAGSVLAGELSLMSAISAGQLVKSHMKYNRST 596
Solanum          QSACLNLLGVKGANREAPGSNARLLATIVAGSVLAGELSLMSAISAGQLVKSHMKYNRSC 569
Picrorhiza       QSACLNLLGVKGASKESPGSNSQLLATIVAGSVLAGELSLMSAISAGQLVKSHMKYNRSS 553
Antirrhinum      QAACLNLLGVKGASKESPGANSRLLATIVAGSVLAGELSLMSAISAGQLVKSHMKYNRSS 540
Oryza            QAACLDLLGVKGANRESPGSNARLLATVVAGGVLAGELSLLSALRAGQLVKSHMKYNRSS 552
Arnebia          QAACLNLLGVKGANRECPGANARQLAAIVAGSVLAGELSLMSAIAAGQLVKSHMKYNRSN 557
Camptotheca      QSACLNLLGVKGASKEAPGSNARLLATIVAGSVLAGELSLMSAIAAGQLVNSHMKYNRSN 585
                 *:***:*******.:*.**:*:: **::***.********:**: *****:********

Nicotiana        KDVTKASS 604
Solanum          KDVTK--- 574
Picrorhiza       RDITNFSS 561
Antirrhinum      RDVTKICS 548
Oryza            KDMSKVIS 560
```

```
Arnebia          KDVSK--- 562
Camptotheca      KDVTKASS 593
                 :*:::
```

(d) Mevalonate Kinase (AeMVK)

```
Arnebia          -----------------------------ATVVINSELPYGSGLGSSAALCVALTAALLA 31
Arabidopsis      QNLPKEKMWLSSGISTFLWLYTRIIGFNPATVVINSELPYGSGLGSSAALCVALTAALLA 162
Medicago         LNIPEAKIGLASGVAAFLWLYSSIQGFKPATVVINSDLPLGSGLGSSAAFCVALAAAFLA 161
Nicotiana        ---------------LFFGCFTSIHGCKPAKAVVSSELPLGSGLGSSAAFCVALSAAILA 45
Zea              QEIPEAKIWLSAGLSAFLFLYASILGCRPGKAVVSSDLPMGAGLGSSAAFCVSMSGALLT 179
Oryza            HEIPEAKIWLSAGLSAFLFLYTSILGCRPGKVTVSSDLPMGSGLGSSAAFCVSMSGVLLT 163
                                              ....:.*:** *:*******:**:::..:*:

Arnebia          S--SISEKTRGNG----WSSLDETNLELLNKWAFEGEKIIHGKPSGIDNTVSAYGGNMIK 85
Arabidopsis      S--SISEKTRGNG----WSSLDETNLELLNKWAFEGEKIIHGKPSGIDNTVSAYG-NMIK 215
Medicago         VTDSVSVDVIRQG----WHSFGEKELDLVNKWAFEGEKIIHGKPSGIDNTVSSYG-NIIS 216
Nicotiana        LSDSVTMEFSHQG----WQVFGENELELVNKWAFEGEKIIHGKPSGIDNTVSTYG-NMIK 100
Zea              AAGAVS-VGARRG-AEGWEVLEKGALELVNQWAFQGEKIIHGKPSGIDNSVSTFG-KMIK 236
Oryza            AAGVVTAVGGISGEGMGWELVGKDDLELVNRWAFQGEKIIHGKPSGIDNAVSTFG-SMIK 222
                     ::      *    *  . :  *:*:*:***:**************:**::* .:*.

Arnebia          FCSGEITRLQSNMPLRMLITNTRVGRNTKALVSGVSQRAVRHPDAVKSVFNAVDSISKEL 145
Arabidopsis      FCSGEITRLQSNMPLRMLITNTRVGRNTKALVSGVSQRAVRHPDAMKSVFNAVDSISKEL 275
Medicago         FKSGNLTRMKSNASLKMLITNTRVGRNTKALVAGVSERMLRHPDAMAFVFTAVDSISKEL 276
Nicotiana        FKSGDLTRLKTNMPLKMLITNTKVGRNTKALVASVSERTLRHPTAMASVFTAVDSISSEV 160
Zea              FKKGELTNLESWNPVKMLITDTRVGRNTKALVAGVSERASRHPDAMASVFHAVNSISEEL 296
Oryza            FKKGELTNLKSSNPVKMLITDTRVGRNTKALVAGVSERASRHSDAMASVFNAVNSISEEV 282
                 * .*::*.:::  .::****:*:*********:.**:*  **. *:  ** **:***.*:

Arnebia          AAIIQS--KDETSVT--------------------------------------------- 158
Arabidopsis      AAIIQS--KDETSVTEKEERIKELMEMNQGLLLSMGVSHSSIEAVILTTVKHKLVSKLTG 333
Medicago         TTVLQSPTPDELSVTTLEEKVEELMEMNQGLLQSMGVSHATIETVLRTTLKYKLSSKLTG 336
Nicotiana        AAIIQSPVPDDLAITEKEEKLEELMEMNQGLLQCMGVSHASIETVLRTTLKYKLSSKLTG 220
Zea              SSIVELAAEDEIAITSKEDKLAELMEMNQGLLQCMGVSHSSIETVLRTTLKYSLVSKLTG 356
Oryza            SSIVELAANDEIAITSKEEKLAELMEMNQGLLQCMGVSHSSIETVLRTTLKFNLVSKLTG 342
                 :::::    *: ::*

Arnebia          ----------------------------------------------------
Arabidopsis      AGGGGCVLTLLP---TGTVVDKVVEELESSGFQCFTALIGGNGAQICY---- 378
Medicago         AGGGGCVLTLLPTLLSPTVVDKVIAELESNGFQCFTAGIGGNGVEISFEHSS 388
Nicotiana        AGGGGCVLTLLPTLLSGTVVDKVIAELETCGFQCLLAGIGGN---------- 262
Zea              AGGGGCVLTLIPTLSANTVLEKVTTELESHGYRCFKVEVGGRGLQVFRG--- 405
Oryza            AGGGGCVLTLIPTRSS---------SLP-------------RRLLLF----- 367
```

(e) Phosphomevalonate Kinase (AePMVK)

```
Arnebia          MAVVASAPGKVLMTGGYLVLERPNAGIVLSTNARFYSVVKPIYDEVKPDSWAWAWADVKL 60
Arabidopsis      MAVVASAPGKVLMTGGYLVLEKPNAGLVLSTNARFYAIVKPINEEVKPESWAWKWTDVKL 60
Hevea            MAVVASAPGKVLMTGGYLILERPNAGIVLSTNARFYAIVKPIYDEIKPDSWAWAWTDVKL 60
Vitis            ------------MTGGYLILERPNAGIVLSTNARFYAIVKPLREDIEPDSWAWAWTDRN- 47
Oryza            ------------------------------------------------------------
Zea              MEVVASAPGKVLIAGGYLVLERPNAGLVLSTTARFYAVVRPLRDSLPADSWTWAWTDVKV 60

Arnebia          TSPQMSREMTYKLSLKYLTLQSVSLSDSRNPFVEYAVQYVVAAAYSR-LDSSGKDALTKL 119
Arabidopsis      TSPQLSRESMYKLSLNHLTLQSVSASDSRNPFVEHAIQYAIAAAHLA-TEKD-KESLHKL 118
Hevea            TSPQLARESLYKLSLKNLALQCVSSSASRNPFVEQAVQFAVAAAHAT-LDKDKKNVLNKL 119
Vitis            ----------YVQNVTKKFNAPMYLSESRNPFVEQAVQYTIAAARAT-LDKNNNDFLHTK 96
Oryza            ----------YKLSLKKSTLQLTSSRESANPFVEQAVQFSVAAAKVTVTDKEGKEALDKL 50
Zea              TSPQLSRVATYKLSLNKTTLQLTSSRESTNPFVEQAIQFSVAAAKATIIDKERKDVVDKL 120
                           *  .:.           * ***** *:*: :***     :.. :: : .

Arnebia          LLRGLDITILGCNEFYSYRNQIEARGLPLTPESLSSLPPFTSITFNKEESG----GQNSK 175
Arabidopsis      LLQGLDITILGSNDFYSYRNQIESAGLPLTPESLGTLAPFASITFNAAESN----GANSK 174
Hevea            LLQGLDITILGTNDFYSYRNEIEACGLPLTPESLAALPSFSSITFNVEEAN----GQNCK 175
Vitis            LLQGLDITILGCNDFYSYRNYIEARGLPLTPDVLAALPPFTPITFNAEESN----GENCK 152
Oryza            LLQGLHITILGCNDFYSYRKQIEARGLPLTPEVLLSLPPFCSITFNSEVANGTMTGENCK 110
Zea              LLQGLNITIIGHNDFYSYRKQIEARGLPLTPEVLLSLPPFSSITFNSEVANGTMTGEKCK 180
```

```
                          **:**.***:* *:*****: **: ******: * :*..* .****   :.    * :.*

Arnebia            PEVAKTGLGSSAAMTTAVVASLLHYLGVVNLSS-VKDN-----SEDLDTVHMIAQTAHCI 229
Arabidopsis        PEVAKTGLGSSAAMTTAVVAALLHYLGVVDLSDPCKEGKF--GCSDLDVIHMIAQTSHCL 232
Hevea              PEVAKTGLGSSAAMTTAVVAALLHHLGLVDLSSSCKEKK----FSDLDLVHIIAQTAHCI 231
Vitis              PEVAKTGLGSSAAMTTSVVAALLHYFGVVNLSSLNKDQHQEKDCVDLDLVHVIAQSAHCI 212
Oryza              PEVAKTGLGSSAAMTTSVVAALLHYLGTVNLSCLGQSSGGNAAGRDLDLLHAIAQSAHCI 170
Zea                PEVAKTGLGSSAAMTTSVVAALLHYLGAVNLSCPGQSSGDNASGRELDLVHTIAQSAHCL 240
                   ****************:***:***::* *:**   :.         :** :* ***::**:

Arnebia            AQGEVGSGFDVSSAVYGSQRYVRFSPGVISSAQDAVKAAPLEEVINDVLKAEWDHEK-DM 288
Arabidopsis        AQGKVGSGFDVSCAVYGSQRYVRFSPEVLSFAQVAVTGLPLNEVIGTILKGKWDNKRTEF 292
Hevea              AQGKVGSGFDVSSAVYGSHRYVRFSPEVLSSAQDAGKGIPLQEVISNILKGKWDHERTMF 291
Vitis              AQGKVGSGFDVSSAVYGSQRYVRFSPEVLSSAQVAVKGLPIQEVIHDILKGNWDHERTKF 272
Oryza              AQGKIGSGFDVSAAVYGSQRYTRFSPEILSSAQVTG-GHCMADVVADIVTQSWDHEKVKF 229
Zea                AQGKIGSGFDVSAAVYGSQRYVRFSPEILSSAQAIG-GTVLPDVVSDVLTQRWDHENKQF 299
                   ***::*******.*****:**.**** ::* **     .  : :*:  ::.  **::.  :

Arnebia            SHAPLMTSIR-EPGTGGSSTPSMVGAVKKWQKADPQTSVETWRKLSEGNAALEMQLNTLS 347
Arabidopsis        SLPPLMNLFLGEPGSGGSSTPSMVGAVKKWQMSDPEKARENWQNLSDANLELETKLNDLS 352
Hevea              SLPPLMSLLLGEPGTGGSSTPSMVGALKKWQKSDTQKSQETWRKLSEANSALETQFNILS 351
Vitis              SLPPLMTLLLGEPGAGGSSTPSMVGAVKKWQKSDPEKSLETWRKLSAANSALETQFKILS 332
Oryza              SLPPLMSLLLGEPGTGGSSTPSMVGSVKQWQKSDPQKSKETWSKLGIANSVLENQLRNLN 289
Zea                SLPPLMTLLLGEPGTGGSSTPSMVGSVKRWLKSDPEKSRDTWSKLAIANSTLENQLRILN 359
                   * .***. :  ***:**********::*:*  :*.:.: :.* :*. .*  ** ::. *.

Arnebia            NLARMSFDVYKDVINNCSTLPSEKWLEVATEPSRTDIVKALLGAKDVMLEIRYQMRKMGE 407
Arabidopsis        KLAKDHWDVYLRVIKSCSVLTSEKWVLHATEPINEAIIKELLEAREAMLRIRILMRQMGE 412
Hevea              KLAEEHWDAYKCVIDSCSTKNSEKWIEQATEPSREAVVKALLGSRNAMLQIRNYMRQMGE 411
Vitis              KLAEENWDAYKCVINSCSIHRSEKWMERATGPDQEAVVNALLAARDAMLRIRSNMRQMGE 392
Oryza              KLAEDHWEAYESVLRSCSRLTCSKWTEVATNQHQELIVRSLLAARDAFLEIRLHMREMGI 349
Zea                GLSENHHEAYESMVRSCSHLTYGKWAEVATNQHQELIIRSLLAARDACLEIRLHMREMGI 419
                    *:.   :.*  :: .**      **    **    .  ::. ** :::. *.**  **:**

Arnebia            AAGIPIEPESQTLLLDSTMNMEGVLLAGVPGAGGFDAVFAVTLGDASDKVIKSWSRQNVL 467
Arabidopsis        AASVPIEPESQTQLLDSTMSAEGVLLAGVPGAGGFDAIFAITLGDSGTKLTQAWSSHNVL 472
Hevea              AAGVPIEPESQTRLLDTTMNMDGVLLAGVPGAGGFDAVFAVTLGDSGTNVAKAWSSLNVL 471
Vitis              AAGIPIEPNSQTRLLDATMDMEGVLLAGVPGAGGFDAIFAVTLGDSSSNITKAWTSLNVL 452
Oryza              AAGVPIEPESQTQLLDATMNMEGVLLAGVPGAGGFDAVFSVILGEASDAVAKAWSSAGVL 409
Zea                AAGVPIEPDSQTRLLDATMNMEGVLLAGVPGAGGFDAVFSVVLGDASNAVAHAWSSVGVL 479
                   **.:****:*** ***:**. :**************:*:: **::.  : ::*:  .**

Arnebia            ALLVREDPNGVLLENNDSRAKEVTSGVSAIQIQ 500
Arabidopsis        ALLVREDPHGVCLESGDPRTTCITSGVSSIHLE 505
Hevea              ALLVREDPNGVLLESGDPRTKEITTAVFAVHI- 503
Vitis              AMLVREDPHGVSLETGDPRAQQITSAISSVHIE 485
Oryza              PLLVREDPRGVSLEAGDPRTREVSTAVSSIQIN 442
Zea                PLPVREDCRGVSLEDADPRTREVSAAVWSIQIN 512
                   .: **** .** **  *.*:  :::.: ::::
```

(f) Mevalonate diphosphate decarboxylase (AeMVDD)

```
Hevea              ----MAESWVIMVTAQTPTNIAVIKYWGKRDEKLILPVNDSISVTLDPVHLCTTTTVAVS 56
Ricinus            ----MAERWVRMVTAQTPTNIAVIKYWGKRDETLILPVNDSISVTLDPAHLCTTTTVAVS 56
Arabidopsis        ---MATEKWVFMVTAQTPTNIAVIKYWGKRDEVRILPVNDSISVTLDPDHLCTVTTVAVS 57
Nicotiana          ----------------TPTNIAVIKYWGKRDEDLILAINDSIGVTLDPAHLCTTTTVAVS 44
Solanum            MAAEQSKKWILMVTAQTPTNIAVIKYWGKRDENLILAINDSISVTLDPAHLCTTTTVAVS 60
Arnebia            -MGEQGENWILMVTAQTPTNIAVIKYWGKRDESLILPINSSISVTLDPSHLCTTTTVSVS 59
                                   ****************  **.:*.**.***** ****.***:**

Hevea              PSFAQDRMWLNGKEISLSGGRYQNCLREIRARACDVEDKERGIKISKKDWEKLHVHIASY 116
Ricinus            PAFDQDRMWLNGKEISLSGGRYQNCLREIRARACDVEDKEKGIKIAKKDWEKLHVHIASF 116
Arabidopsis        PAFDRDRMWLNGKEISLSGSRYQNCLREIRGRAGDVEDMEKGIKIRKKDWEKLNLHIASH 117
Nicotiana          PAFNQDRMWLNGKEISLSGGRYQNCLREIRARASDVEDENKGIKIAKKDWQNLHVHIASY 104
Solanum            PSFQQDRMWLNKKEISLDGARYQNCLREIRARANDYEDEKKGIKISKNDWQNLHVHIDSY 120
Arnebia            PSFKQDCMWLNGKEISLSGGRFQRCLREIRSRACDVEDEKKGFKIAKKDWEKLHVHIASY 119
                   *:*  :* **** *****.*.*:*.******.** * **  ::*:** *:**::*::** *.

Hevea              NNFPTAAGLASSAAGFACLVFALAKLMNAKEDHSELSAIARQGSGSACRSLFGGFVKWKM 176
Ricinus            NNFPTAAGLASSAAGFACLVFALAKLMNAREDNSELSAIARQGSGSACRSLFGGFVKWIM 176
Arabidopsis        NNFPTAAGLASSAAGFACLVFSLAKLMNVDEDPSHLSAIARQGSGSACRSLFGGFVKWTM 177
Nicotiana          NNFPTAAGLASSAAGFACLVFSLAKLMNVQEDNGKLSAIARQGSGSACRSLYGGFVKWIM 164
Solanum            NNFPTAAGLASSAAGFACLVFSLAKLMNVQEDNGRLSAIARQGSGSACRSLFGGFVKWVM 180
```

```
Arnebia           NNFPTAAGLASSAAGFACLVFSLAKLMNLKEDHGQLSAIARQGSGSACRSLFGGFVKWDM 179
                  *********************:******  ** ..****************:****** *

Hevea             GKVEDGSDSLAVQVVDEKHWDDLVIIIAVVSSRQKETSSTTGMRETVETSLLLQHRAKEI 236
Ricinus           GKVDDGSDSLAVQLVDEKHWDDLVIIIAVVSSRQKETSSTSGMRESVETSLLLQHRAKEV 236
Arabidopsis       GSKEDGSDSVAVQLADEKHWDDLVIIIAVVSSRQKETSSTSGMRESVETSLLLQHRAKEV 237
Nicotiana         GKEENGSDSIAVQLVDEKHWDELVIIIAVVSSRQKETSSTSGMRDTVETSALIEHRAKEV 224
Solanum           GKEEDGSDSIAVPLADEKHWDELVIIIAVVSSRQKETSSTSGMRETVVTSALIDHRAKEV 240
Arnebia           GKESDGSDSIAIPLVDEKHWDELVIVIAVVSAHQKETSSTSGMRDTVETSPLIQHRAKEV 239
                  *. .:****:*: :.******:***:*****::*******:***::* ** *::*****:

Hevea             VPKRIVQMEESIKNRNFASFAHLTCADSNQFHAVCMDTCPPIFYMNDTSHRIISCVEKWN 296
Ricinus           VPKRIIQMEEAINKRDFASFAQITCADSNQFHAVCLDTCPPIFYMNDTSHRIISCVEKWN 296
Arabidopsis       VPKRILQMEEAIKNRDFASFTQLTCTDSNQFHAVCLDTSPPIFYMNDTSHRIISLVEKWN 297
Nicotiana         VPKRIVQMEEAIQNRDFPTFAQLTCADSNQFHAVSMDTSLPIFYMNDTSHRVIGCVEKWN 284
Solanum           VPKRIVQMEEAIQNRDFPTFAQLTCSDSNQFHAVCMDTSPPIFYMNDTSHRVISCVEKWN 300
Arnebia           VPKRIVQMEEAISNRDFSTFAHLSCSDSNQFHAVCLDTSPPIFYMNDTSHRIISLVEKWN 299
                  *****:****:*.:*:*.:*::::*:********.:**. ***********:*. *****

Hevea             RSVGTPQVAYTFDAGPNAVLIAHNRKAAAQLLQKLLFYFPPNSDTELNSYVLGDKSILKD 356
Ricinus           RSEETPQVAYTFDAGPNAVLIAQNRKTAVQLLQKLLYYFPPNSDTDLNSYVLGDKSILKD 356
Arabidopsis       RSEGTPQVAYTFDAGPNAVLIARNRKVAVQLLQGLLYYFPPKSDTDMKSYVVGDNSILKE 357
Nicotiana         RSEGTPQVAYTFDAGPNAVLIARNRKAATLMLQRLLFHFPPNSDTDLNSYVIGDKSILKD 344
Solanum           RAEGTPQVAYTFDAGPNAVLIARNRKAATLMLQRLLFHFPPNSDTDLDSYVIGDKSILKD 360
Arnebia           RSEGTPQVAYTFDAGPNAAMIARNRKVATLLLQKLLYCFPPQADADLDSYVIGDKSLLKE 359
                  *:  **************.:**:***.*. :** **: ***::*:::.***:**:*:**:

Hevea             AGIEDLKDVEALPPPPEIKD---APRYKGDVSYFICTRPGRGPVLLSDESQALLSPETGL 413
Ricinus           AGIEEMKDVESLPAPPEIKD---APRFKGDVSYFICTRPGRGPVLLTDESQALLNPQTGL 413
Arabidopsis       AGLDGASGVENLQPPPEIKDNIGSQDQKGEVSYFICTRPGKGPIVLHDQTQALLDPETGL 417
Nicotiana         AGIQDIKDVEALPPPPEIKDKVPAQKYKGEISYFICTRPGRGPVLLTDDSHALLNPETGL 404
Solanum           AGIKDLNDIEALPPPPEIKDKVPAQKCKGEISYFICTRPGRGPVLLPDESQALLCLETGL 420
Arnebia           AGVGTMNDVDALAPPPELTTSVPAQRTKGDVSYFICTRPGKGPVLLTDENQALLDSKTGL 419
                  **:   ..:: * .***:.    :   **::*********:**::* *:.:***  :***

Hevea             PK 415
Ricinus           PK 415
Arabidopsis       PK 419
Nicotiana         PK 406
Solanum           PK 422
Arnebia           PK 421
                  **
```

(g) Isopentenyl pyrophosphate isomerase (AeIPPI)

```
Nicotiana         ------------------------------------------------------------
Solanum           ------------------------------------------------------------
Pueraria          MAQSSIVVNLSQHLFKRNLTSLSVIAHSSYFGSRSLSSLSFSQRRRGPHTPLTLTASFSS 60
Camptotheca       ------------------------------------------------------------
Arnebia           ------------------------------------------------------------
Adonis            --MSSIRINPLYSIFSTTTKTLSASCSSPAVHLQQRCRTLSISSSITNSPRRGLNRLFAS 58

Nicotiana         ---MGDV---EADANMDAVQRRLMFEDECILVDENDQVVGHDTKYNCHLMERIESLNLLH 54
Solanum           ---MVDV---IADANMDAVQRRLMFDDECILVDVNDKVVGHESKYNCHLMEKIESENLLH 54
Pueraria          HTAMGETSAPAADAGMDAVQRRLMFEDECILVDENDRVVGHDSKYNCHLMEKIEAENLLH 120
Camptotheca       ---MGDA---ATDAGMDAVQKRLMFEDKCILVDENDNVVGHDTKYNCHLMEKIESENLLH 54
Arnebia           ---------MAADAGMDAVQRRLMFEDECILVDENDNVVGHDTKYNCHLMEKIESENLLH 51
Adonis            TSTMGEV----ADAGMDAVQKRLMFDDECILVDENDKVVGHDSKYNCHLMEKIEAENLLH 114
                             :**.*****:****:*:***** **.****::********:**: ****

Nicotiana         RAFSVFLFNSKYELLLQQRSATKVTFPLVWTNTCCSHPLYRESELIEENALGVRNAAQRK 114
Solanum           RAFSVFLFNSKYELLLQQRSATKVTFPLVWTNTCCSHPLYRESELIEENALGVRNAAQRK 114
Pueraria          RAFSVFLFNSKYELLLQQRSATKVTFPLVWTNTCCSHPLYRESELIEENALGVRNAAQRK 180
Camptotheca       RAFSVFLFNSKYELLLQQRSATKVTFPLVWTNTCCSHPLYRESELIKENALGARNAAQRK 114
Arnebia           RAFSVFLFNSKHELLLQQRSATKVTFPLVWTNTCCSHPLYRESELIKENFLGVRNAAQRK 111
Adonis            RAFSVFLFNSKYELLLQQRSATKVTFPLVWTNTCCSHPLFRDSELIEENFLGVRNAAQRK 174
                  ***********:***************************:*:****:** **.*******

Nicotiana         LLDELGIPAEDVPVDQFTPLGRMLYKAPSDGKWGEHELDYLLFIVRDVNVNPNPDEVADI 174
Solanum           LLDELGIPAEDVPVDQFTPLGRMLYKAPSDGKWGEHELDYLLFIVRDVNVHPNPDEVADI 174
Pueraria          LLDELGIVAEDVPVDQFTPLGRILYKAPSDGKWGEHELDYLLFIVRDVNVNPNPDEVADI 240
Camptotheca       LLDELGIPAEDVPVDQFIPLGRILYKAPSDGKWGEHELDYLLFIVRDVNVNPNPDEVADI 174
```

```
Arnebia          LLDELGIPTEDVPVDQFTPLGRILYKAPSDGKWGEHELDYLLFIVRDVKVNPNPDEVADI 171
Adonis           LLDELGIPAEDVPVDEFTPLGRILYKAPSDGKWGEHELDYLLFIVRDVKYDPNPDEVADA 234
                 ******* :******:* ****:*************************: .********

Nicotiana        KYVNQEQLKELLRKADAGEDGLKLSPWFRLVVDNFLFKWWDHVEKGTIQEAADMKTIHKL 234
Solanum          KYVNQEQLKELLRKADAGEEGLKLSPWFRLVVDNFLFKWWDHVEKGTIQEAADMKTIHKL 234
Pueraria         KYVNRDQLKELLRKADAGEEGLKLSPWFRLVVDNFLFKWWDHLEQGTLGEVTDMKTIHKL 300
Camptotheca      KYVTRDQLKELLRKADAGEEGLKLSPWFRLVVDNFLFKWWDHVEKGTMHEAADMKSIHKL 234
Arnebia          KYVNPAQLKELLTKADAGEDGLKLSPWFRLIVDNFLFKWWDHVEKGNYHQVADMKTIHKL 231
Adonis           KYVNREELKEILRKADAGEEGIKLSPWFRLVVDNFLFKWWDHVEEGKIKDVADMKTIHKL 294
                 ***.  :***:* ******:*:********:***********:*:*.  :.:***:****

Nicotiana        T 235
Solanum          T 235
Pueraria         T 301
Camptotheca      I 235
Arnebia          T 232
Adonis           T 295
```

(h) Geranyl diphosphate synthase (AeGDPS)

```
Antirrhinum      ------MSLVNPITTWSTTTTSKSPKNVQTTTRSRSIILPHKIS-------LFPSNPKSK 47
Mentha           -----MSALVNPVAKWPQTIGVKDVHGGRRRRSRSTLFQSHPLRTEM----PFSLYFSSP 51
Picrorhiza       ------MSLVNSIT-WSQTSSILNIQSNISKKLTPFSILPHPLTNNL----PISLFPNPK 49
Humulus          ------MSSVN--LTWVQTCSMFN-QAGRSRSSTFNLLHYHPLK-KV----PFSFQTPKQ 46
Arnebia          -----MHNLRAHVKKWIQLSSIFS-------CSSKSIYMSSRVN---------------- 32
Abies            MAYSCMAASC-HGLHFMNIASQECNLKRGIIPSKRLHGISSSLWASN----GFQGHLERD 55
Picea            MGYNGMVVSSNLGLYYLNIASRECNLKRISIPSP-FHGVSTSLGSSTSXHLGLRGHLKKE 59
                                :                          :

Antirrhinum      S-KTHLRFSISSILTKNPQESSQK-TSKDPTFTLDFKTYMLEKASSVNKALEQAVLLKEP 105
Mentha           L-KAPATFSVSAVYTKEGSEIRDKDPAPSTSPAFDFDGYMLRKAKSVNKALEAAVQMKEP 110
Picrorhiza       SNISNSNTPLSAILTKD-----QKPQNPPTTPTFDFKSYMLQKADSVNKALDDSIPLTEP 104
Humulus          RRPTSSFSSISAVLTEQ----EAVTEDEEQKSTFNFKSYMVQKANSVNQALDAAVLLREP 102
Arnebia          ------------VFEIE----------EEPRSRFDFKSYMINKITSINEALDSAVPLIEP 70
Abies            LSAYRHLVSSSRCLNTIAMLSNLSEQAKEKATEFDFKEYLHSKAISVNEALERAVPLRYP 115
Picea            LLSHRLLLSSTRSSKALVQLADLSEQVKN-VVEFDFDKYMHSKAIAVNEALDKVIPPRYP 118
                                                  ::*. *:  *  ::*:**:  :    *

Antirrhinum      LKIHESMRYSLLAGGKRVRPMLCIAACELVGGLESTAMPSACAVEMIHTMSLIHDDLPCM 165
Mentha           LKIHESMRYSLLAGGKRVRPMLCIAACELVGGDESTAMPAACAVEMIHTMSLMHDDLPCM 170
Picrorhiza       LKIQESMRYSLLAGGKRIRPMLCIAACELVGGDESTAMPAACAVEMVHTMSLMHDDLPCM 164
Humulus          QMIHEAMRYSLLAGGKRVRPVLCLSACELVGGDESVAMPAACAVEMIHTMSLIHDDLPCM 162
Arnebia          IKLHEAMRYTLLSGGKRVRPIVCIAACELVGGHESTVMPTACAQEMIHSMSVMLDDLPCM 130
Abies            EKIHEAMRYSLLAGGKRIRPILTIAACELVGGSEELAMPTACAMEMIHTMSLIHDDLPSM 175
Picea            QKIYESMRYSLLAGGKRVRPILCIAACELMGGTEELAMPTACAIEMIHTMSLIHDDLPYI 178
                   : *:***:**:****:**:: ::****:** *. .**:*** **:*:**:: **** :

Antirrhinum      DNDDLRRGKPTNHKIYGEDVAVLAGDALLAFSFEHVAKST-KGVSSDRIVRVIGELAKCI 224
Mentha           DNDDLRRGKPTNHMAFGESVAVLAGDALLSFAFEHVAAAT-KGAPPERIVRVLGELAVSI 229
Picrorhiza       DNDDLRRGKPTNHKVFTEDVAVLAGDAMLAFSFEHVASLT-KGVCSERIVRVIYELAKCV 223
Humulus          DNDDLRRGKPTNHKVFGEDVAVLAGDALLAYAFEHVAVST-VGVPAARIIRAIGELAKSI 221
Arnebia          DNDDFRRGKLSNHRVYGEKITLLAVRSLQALAVDHVVTAT-RGVPPERLVRALTEMAILT 189
Abies            DNDDLRRGKLTNHKVFGEGTAVLAGDALLSFAFEHIAVSTRKTVASHRVLRVVSELGKAI 235
Picea            DNDDLRRGKPTNHKVFGEDTAIIAGDALLSLAFEHVAVSTSRTLGTDIILRLLSEIGRAT 238
                 ****:**** :**  : *  :::*  :: : :.:*:.  *     .  ::* : *:.

Antirrhinum      GSEGLVAGQVVDISSEG-MTEVGLEHLEFIHVHKTAALLEASVVLGAIVGGADDEDVEKL 283
Mentha           GSEGLVAGQVVDVCSEG-MAEVGLDHLEFIHHHKTAALLQGSVVLGAILGGGKEEEVAKL 288
Picrorhiza       GCEGLVAGQVVDICSEG-MDEVGLEHLEFIHLNKTAALLEGSVVLGAILGGGSDEEVEKL 282
Humulus          GSEGLVAGQVVDIDSEG-LANVGLEQLEFIHLHKTAALLEASVVLGAILGGGTDEQVEEL 280
Arnebia          GSKGAAAGQIADLCSSGEDFNVSIEQLEHIHMQKTGTLCEGSVVSGAIIGGASEEEIEKL 249
Abies            GSQGVAGGQVADITSEG-DPSVGLETLEWIHIHKTAVLLECAVVSGAIIGGASENEIERT 294
Picea            GSEGVMGGQVVDIESEG-DPSIDLETLEWVHIHKTAVLLECSVVCGAIMGGASEDDIERA 297
                 *.:*  .**:.*: *.*   .:.:: ** :* :**..* : :** ***:**. :::: .

Antirrhinum      RKFARCIGLLFQVVDDILDVTKSSQELGKTAGKDLVADKTTYPKLLGIEKSREFAEKLNR 343
Mentha           RKFANCIGLLFQVVDDILDVTKSSKELGKTAGKDLVADKTTYPKLIGVEKSKEFADRLNR 348
Picrorhiza       RNFARCIGLLFQVVDDILDVTKSSKELGKTAGKDLVADKTTYPKLIGIEKSKEFAERLNR 342
Humulus          RSFARCIGLLFQAVDDILDVTKSSQELGKTAGKDLVADKATYPRLMGIEKSREFAEKLNR 340
Arnebia          RKFSKCIGLMFQIVDDVLDVTKSSMELGKPAGKDVVAAKATYPRLIGIEKSRELALKLNS 309
```

```
Abies           GRYARCVGLLFQVVDDILDVTRSSEELGKTAGKDLVSDKATYPKLMGLEKAKEFADELLD 354
Picea           RRYARCVGLLFQVVDDILDVSQSSEELGKTAGKDLISDKATYPKLMGLEKAKEFADELLN 357
                  ::.*:**:** ***:***::** ****.****::: *:***:*:*:**::*:* .*

Antirrhinum     EAQEQLEGFDSVKAAPLIALANYIAYRDN 372
Mentha          EAQEQLLHFHPHRAAPLIALANYIAYRDN 377
Picrorhiza      EAKEHLAGFDQNKAAPLIALADYIAYRDN 371
Humulus         EAQEHLGGFDPQKAAPLIALANYIAYRQN 369
Arnebia         EAKEQLSGFDQEKAAPLIALADYIVARQN 338
Abies           RAKEELSCFNPAKAAPLLGLADYIALRQN 383
Picea           RGKQELSCFDPTKAAPLFALADYIASRQN 386
                ..::.*  *.  :****:.**:**. *:*
```

(i) *p*-Hydroxybenzoic acid geranyltransferase (AePGT)

```
Arabidopsis     ------------------------------------------------------------
Capsella        MAFFGLFRVSRRLLRSSVSATPSSSFAVLQSQHKSLSNPVNTHCTNPFTKCYPSWNYNYQ 60
Oryza           ------------------------------------------------------------
Arnebia         ------------------------------------------------------------
Lithospermum    ------------------------------------------------------------


Arabidopsis     ----------EKFFGVGWNYRLICGMSSSSSVLEGKPKKDDKEKSDGVVVKEASWIDLYL 50
Capsella        VWSKGRELQQEKFLGVGWNYRLVSGMSSSSSVMEGKPKKDDKEKSDVGGVKEASWIDLYL 120
Oryza           ------------------------------------------------------------
Arnebia         --------------------------------MTSKQAQQKKGK-------QPSWIELYL 21
Lithospermum    -------------------------------MVSSKQTQLKKGK-------QPSWIEMYL 22


Arabidopsis     PEEVRGYAKLARLDKPIGTWLLAWPCMWSIALAADPGSLPSFKYMALFGCGALLLRGAGC 110
Capsella        PQDARGYAKLARLDKPIGTWLLAWPCMWSIALAADPGSLPSFKYMALFGCGALLLRGAGC 180
Oryza           -------------------------------MAAMPGELPDMRMLALFACGSVLIRGAGC 29
Arnebia         PKEVRPYAHLARLDKPIGSWLLAWPAFWSVALVADFGSLP--KMLAIFGWWAVWIRGAGC 79
Lithospermum    PKEVRPYAHLARLDKPIGSWLLAWPAFWSVALAADLESLP--KMLAIFGWWAVWIRGAGC 80
                                               :.*   .**  : :*:*.  :: :*****

Arabidopsis     TINDLLDQDIDTKVDRTKLRPIASGLLTPFQGIGFLGLQLLLGLGILLQLNNYSRVLGAS 170
Capsella        TINDLLDQDIDTKVDRTRLRPIASGLLTPFQGIQFLGIQLLLGLGILLQLNNYSRVLGAS 240
Oryza           TINDLLDRDIDRKVERTKSRPLASGILTPTQGVGFLGFQLLLGLPILLQLNNLSQILGVF 89
Arnebia         TINDYFDRDFDKKVERTKSRPLASGAVSPSQGLWWLAFQLFIGLGVLYQFNILTLALAIL 139
Lithospermum    TINDYFDRNFDKKVERTKSRPLASGAVSPAQGLWWLAFQLFIGLGVLYQFNVLTLALAIV 140
                **** :*:::* **:**: **:*** ::* **: :*.:**::** :* *:*  :  *.

Arabidopsis     SLLLVFSYPLMKRFTFWPQAFLGLTINWGALLGWTAVKGSIAPSIVLPLYLSGVCWTLVY 230
Capsella        SLLLVFSYPLMKRFTFWPQAFLGLTINWGALLGWTAVKGSIEPAVVLPLYLSGVCWTLVY 300
Oryza           SLLLVFSYPLMKRFTYWPQAYLGLVFNWGALIGWAAMKGTIDPAIILPLYTAGICWTLVY 149
Arnebia         HVPLVFAYPLMKRITYWPQAFLGVMISWGALLGSSALKGSVVPSIAYPLYISSFFWTLVY 199
Lithospermum    HVPFVFAYPLMKRITYWPQAFLGVMISWGALLGSSALKGSVVPSIAYPLYISSFFWTLVY 200
                 : :**:******:*:****:**: :.****:* :*:**:: *::  *** :.. *****

Arabidopsis     DTIYAHQDKEDDVKVGVKSTALRFGDNTKLWLTGFGTASIGFLALSGFSADLGWQYYASL 290
Capsella        DTIYAHQDKEDDIKVGVKSTALRFGDNTKLWLTGFGTASMGFLALSGLSADLGWQYYASL 360
Oryza           DTIYAHQDKEDDVKVGVKSTALRFGDSTKPWISGFGAACIANLALSGYNADLAWPYYPFL 209
Arnebia         DTIYAHQDKVDDAKAGIKSTALRFGDATKIWISWFGVGCIAALVIGGLIVNIGFPYYVFV 259
Lithospermum    DTIYAHQDKVDDAKAGIKSTALRFGDATKMWISWFGVGCIAALVIGGLILNIGLPYYVFV 260
                ********* ** *.*:********* ** *:: **...:. *.:.*   ::.  **  :

Arabidopsis     AAASGQLGWQIGTADLSSGADCSRKFVSNKWFGAIIFSGVVLGRSFQ 337
Capsella        AAASGQLGWQIGTADLSSGADCSRKFVSNKWFGAIVFSGVVLGRSFQ 407
Oryza           AAASAHLAWQVSTVDLSDRLDCNRKFVSNKWFGALIFGGILCGRLVS 256
Arnebia         AIATGQLAWQIVTVDLSSPMDCGRKFVSNQWFGAIIFTGILLGRLFT 306
Lithospermum    AIATGQLVWQIFTVDLLSPLDCGKKFVSNQWFGAIIFTGILLGRLFP 307
                * *:.:* **: *.** .  **.:*****:****::* *:: ** .
```

(j) Phenylalanine ammonia lyase (AePAL)

```
Arnebia         -METIVEKG----NGKSMEFCMQDPLNWEMAAESMKGSHLDEVKRMVAEFRKPVVQLAGK 55
Lithospermum    -----MENG----NGK-MEFCMKDPLNWGMAAESMKGSHLDEVKKMVAEFRKPVVQLAGK 50
Nicotiana       MASNGHVNG-GEDFELCKKSSATDPLNWEMASESLRGSHLDEVKKMVSEFRKPMVKLGGE 59
Capsicum        MASTIAQNGDIVAIDLCNKS-IHDPLNWEMAAESLKGSHLDEVKKMVDEFRKPIVKLGGE 59
Catharanthus    MKNMGSTENGHINNGEVLDFCLKDPLNWGMAADSLKGSHLDEVKRMVAEFRKPVVKLGGE 60
```

```
Camellia            -MDSTTAIGNGVGSGGSPGFCLKDPLNWGVAAEAMKGSHLKEVKGMVEEFRKPVVRLGGE 59
                            .              ***** :*:::::****.*** ** *****:*:*.*:

Arnebia             TLTIGQVASIAAHDD---GVKVELAEAAREGVKASSDWVMDSMNKGTDSYGVTTGFGATS 112
Lithospermum        TLTIAQVAAIAARDD---GVTVELAEAAREGVKASSDWVMESMNKGTDSYGVTTGFGATS 107
Nicotiana           TLTVAQVAAIAVRDKSANGVKVELSEDARAGVKASSDWVMDSMNKGTDSYGVTTGFGATS 119
Capsicum            TLTVAQVASIANADNKTCGVKVELSESARAGVKASSDWVMDSMCKGTDSYGVTTGFGATS 119
Catharanthus        TLTISQVAAIAARDY--NAVKVELSEDARAGVKASSDWVMESMNKGTDSYGVTTGFGATS 118
Camellia            TLTISQVAAIAVRGS---EVAVELSESAREGVKASSDWVMESMNKGTDSYGVTTGFGATS 116
                    ***:.***:**  .     * ***:* ** **********:** ****************

Arnebia             HRRTKQGGALQKELIRFLDAGIFGNGTETSHTLPHSATRAAMLVRINTLLQGYSGIRFEI 172
Lithospermum        HRRTKQGGALQKELIRFLNAGIFGNGTETSHTLPHSATRAAMLVRINTLLQGYSGIRFEI 167
Nicotiana           HRRTKNGGALQKELIRFLNAGVFGNGTETNHTLPHSATRAAMLVRINTLLQGYSGIRFEI 179
Capsicum            HRRTKNGGALQKELIRFLNAGVFGNGTGSCHTLPHSATRAAMLVRINTLLQGYSGIRFEI 179
Catharanthus        HRRTKQGGALQKELIRFLNAGIFGNGTESSHTLPHSATRAAMLVRINTLLQGYSGIRFEI 178
Camellia            HRRTKEGGALQKELIRFLNAGIFGNGTESCHTLPQSATRAAMLVRINTLLQGYSGIRFQI 176
                    *****:************:**:***** : ****:***********************:*

Arnebia             LEAITKFLNSNITPFLPLRGTISSSGDLVPLSYIAGLLTGRPNS-RVGPKGEKLNAEEAF 231
Lithospermum        LEAITKFLNTNITPCLPLRGTITASGDLVPLSYIAGLLTGRPNSKAVGPTGEKLNAEEAF 227
Nicotiana           LEAITKLINSNITPCLPLRGTITASGDLVPLSYIAGLLTGRPNSKAVGPNGETLNAEEAF 239
Capsicum            LEAITKLINSNITPCLPLRGTITASGDLVPLSYIAGLLTGRPNAKAVGPNGEKLNAEEAF 239
Catharanthus        LEAITKFLNHNITPCLPLRGTITASGDLVPLSYIAGLLTGRPNSKAVGPNGEIVNPEQAF 238
Camellia            LETISKFLNNNITPCLPLPGTITASGDLIPLSYIAGLLTGPPHSKAVGPTGKTFPPKEAF 236
                    **:*:*::* **** *** ***::****:*********** *::  ***.*: . .::**

Arnebia             RLAGISNGFFELQPKEGLALVNGTAVGSGMASMVLYEATILAVLSEVISAIFAEVMNGKP 291
Lithospermum        RLAGISSGFFELQPKEGLALVNGTAVGSGMASMVLYEANILGVMSEVLSAVFAEVMNGKP 287
Nicotiana           RVAGVNGGFFELQPKEGLALVNGTAVGSGMASMVLFDSNILAVMSEVLSAIFAEVMNGKP 299
Capsicum            RVAGVSGGFFELQPKEGLALVNGTAVGSGMASMVLFESNILAVMSEVLSAIFAEVMNGKP 299
Catharanthus        KMAGVNDGLFELQPKEGLALVNGTAVGSGMASMVLFEANILAVLSEVLSAIFAEVMNGKP 298
Camellia            PLTWNEGGFFDLQPKEGLALVNGTAVGSGLASMVLFEANILAVLSEVLSAIFAEVMQGKP 296
                     ::  ..*:*:******************:*****:::.**.*:***:**:*****:***

Arnebia             EFTDHLTHKLKHHPGQIEAAAIMEHILDGSGYVKAAQKLHEMDPLQKPKQDRYALRTSPQ 351
Lithospermum        EFTDHLTHKLKHHPGQIEAAAIMEHILDGSGYVKAAELLHEMDPLQKPKQDRYALRTSPQ 347
Nicotiana           EFTDHLTHKLKHHPGQIEAAAIMEHILDGSSYVKAAQKLHEMDPLQKPKQDRYALRTSPQ 359
Capsicum            EFTDHLTHKLKHHPGQIEAAAIMEHILDGSSYVKAAQKLHEMDPLQKPKQDRYALRTSPQ 359
Catharanthus        EFTDHLTHKLKHHPGQIEAAAIMEHILDGSGYVKAAHKLHEMDPLQKPKQDRYALRTSPQ 358
Camellia            EFTDHLTHKLKHHPGQIEAAAIMEHILDGSSYVKAAQKLHEMDPLQKPKQDRYALRTSPQ 356
                    ******************************.*****. **********************

Arnebia             WLGPLIEVIRSATKMIEREINSVNDNPLIDVSRNKALHGGNFQGTPIGVAMDNTRLAIAS 411
Lithospermum        WLGPQIEVIRSATKMIEREINSVNDNPLIDVSRNKALHGGNFQGTPIGVAMDNTRLAIAA 407
Nicotiana           WLGPQIEVIRSATKMIEREINSVNDNPLIDVSRNKALHGGNFQGTPIGVSMDNARLALAS 419
Capsicum            WLGPQIEVIRAATKMIEREINSVNDNPLIDVSRNKALHGGNFQGTPIGVSMDNTRLALAS 419
Catharanthus        WLGPQIEVIRSATKMIEREINSVNDNPLIDVSRNKALHGGNFQGTPIGVSMDNARLAIAS 418
Camellia            WLGPLIEVIRSSTKSIEREINSVNDNPLINVSRNKALHGGNFQGTPIGVSMDNTRLAVAS 416
                    **** *****::** **************:*******************:***:***:*:

Arnebia             IGKLLFAQFSELVNDYYNNGLPSNLTGSRNPSLDYGFKGAEIAMASYCSELQFLANPVTN 471
Lithospermum        IGKLLFAQFSELVNDYYNNGLPSNLTGSRDPSLDYGFKGAEIAMASYCSELQFLANPVTN 467
Nicotiana           IGKLMFAQFSELVNDYYNNGLPSNLTASRNPSLDYGFKGAEIAMASYCSELQFLANPVTN 479
Capsicum            IGKLMFAQFSELVNDYYNNGLPSNLTAGRNPSLDYGFKGAEIAMASYCSELQFLANPVTN 479
Catharanthus        IGKLMFAQISELVNDFYNNGLPSNLSGGRNPSLVYGFKGAEIAMASYCSELQYLANPVTN 478
Camellia            IGKLMFAQFSELVNDFYNNGLPSNLSGGRNPSLDYGFKGAEIAMAAYCSELQFLANPVTN 476
                    ****:***:******:*********:..*:*** ***********:******:*******

Arnebia             HVQSAEQHNQDVNSLGLISSRKTSEAVEILKLMSSSFLVALFQAVDLRHIEENVRLAVKN 531
Lithospermum        HVQSAEQHNQDVNSLGLISSRKTSEAVEILKLMSSSFLVALCQAVDLRHIEENVRLAVKK 527
Nicotiana           HVQSAEQHNQDVNSLGLISARKTAEAVDILKLMSSTYLVALCQAIDLRHLEENLKNAVKN 539
Capsicum            HVQSAEQHNQDVNSLGLISSRKTAEAVDILKLMSSTYLVALCQAIDLRHLEENLKNAVKN 539
Catharanthus        HVQSAEQHNQDVNSLGLISSRKTAEAVEILKLMSSTYLVALCQAIDLRHLEENLKNAVKN 538
Camellia            HVQSAEQHNQDVNSLGLISSRKTAEAVDILKLMSSTYLVALCQAVDLRHFEENLRNTVKS 536
                    *******************:***:***:*******::**** **:****:***:: :**.

Arnebia             TVSQVAKRTLTTGVNGELHPSRFSEKDLLLVVDREYVFAYADDPCLATYPLMQKLRDVLV 591
Lithospermum        TVSQVAKKTLNIGVDGVLHPSRFSEKELLRVVDREYVFAYADDPCSATYPLMQKLREVLV 587
Nicotiana           TVSQVAKRTLTMGANGELHPARFCEKELLRVVDREYLFAYADDPCSCNYPLMQKLRQVLV 599
Capsicum            TVSQVAKRTLTMGANGELHPARFCEKELLRVVDREYLFAYADDPCSSTYPLMQKLRQVLV 599
Catharanthus        TVSQVAKRTLTVGVNGELHPSRFCEKDLIRVVDREYVFAYVDDPCSGTYPLMEKLRQVIV 598
Camellia            TVSQVAKRVLTMGVNGELHPSRFCEKDLLRVVDREYIFAYIDDPCSATYPLMQKLRQVLV 596
                    *******:.*. *.:* ***:**.**:*: ******:*** ****  .****:***:*:*

Arnebia             GHALANGENEKDVNTSIFHKIAIFEDELKAILPKEVENARASVENGTPAILNRIEECRSY 651
Lithospermum        SHALANSGNEKDASTSIFHKIGVFEEELKGILPKEVENARASVENGTPAIPNKIEECRSY 647
```

```
Nicotiana       DHAMKNGESEKNVNSSIFQKIGAFEDELKAVLPKEVENARAALESGNPAIANRITECRSY 659
Capsicum        DHALNNGESEKNVNSSIFQKIAAFEDELKAVLPKEVESARITLESGNPSIPNRITECRSY 659
Catharanthus    DHALQNGESEKNVNTSIFQKIAAFEDELKTVLPKEVESARTALENGNPAIPNRIKECRSY 658
Camellia        EHALKNGESEKNLSTSIFQKIRAFEEEIKTLLPKEVESTRAAIENGNSAIPNRIKECRSY 656
                 **: *. .**: .:***:**  **:*:* :******.:* ::*.*..:* *:* *****

Arnebia         PLYKFVREELGTEFLTGEKVRSPGEELDKVFTALCEGKLVDPLLACLEAWNVAPLPIC 709
Lithospermum    PLYKFVRGELGTELLTGEKVRSPGEELDQVFNALCEGKLVDPLLACLEAWNGAPLPIC 705
Nicotiana       PLYRFVRKELGTELLTGERVRSPGEECDKVFTAMCNGQIIDPMLECLKSWNGAPLPIC 717
Capsicum        PLYRLVRKELGTELLTGERVRSPGEEIDKVFTAMCNGQIIDPLLECLKSWNGAPLPIC 717
Catharanthus    PLYKFVREDVGAEFLTGEKDRSPGEEFDKVFTAMCNEKIIDPLLECLKEWNGAPLPIC 716
Camellia        PLYKFVREELGTELLTGEKVRSPGEEFDKVFTALCKGEMIDPLMDCLKEWNGAPLPIC 714
                ***::** ::*:*:****: ****** *:**.*:*: :::**:: **: ** ******
```

(k) Cinnamic acid 4-hydroxylase (AeC4H)

```
Arnebia         MDLLLLEKALLGLFFSVIIAIVISKLGGKKFKLPPGPIPVPIFGNWLQVGDDLNHRNLTE 60
Lithospermum    MDLLLLEKALIGLFFSFIIAIVISKLRGKKFKLPPGPIPVPIFGNWLQVGDDLNHRNLTE 60
Solanum         MDLLLLEKTLIGLFFAILIAIIVSKLRSKRFKLPPGPIPVPVFGNWLQVGDDLNHRNLTE 60
Capsicum        MDLLLLEKTLIGLFFAILVAIIVSKLRSKRFKLPPGPIPVPVFGNWLQVGDDLNHRNLTD 60
Camellia        ------------------------------------------------------------
Brassica        MDLLLLEKSLIAVFAAVVLATVISKLRGKKLKLPPGPMPIPIFGNWLQVGDDLNHRNLVD 60
Arabidopsis     MDLLLLEKSLIAVFVAVILATVISKLRGKKLKLPPGPIPIPIFGNWLQVGDDLNHRNLVD 60


Arnebia         YAKKFGEIFLLRMGQRNLVVVSSPDLAKEVLHTQGVEFGSRTRNVVFDIFTGKGQDMVFT 120
Lithospermum    YAKKFGEIFLLRMGQRNLVVVSSPDLAKEVLHTQGVEFGSRTRNVVFDIFTGKGQDMVFT 120
Solanum         YAKKFGDVFLLRMGQRNLVVVSSPELAKEVLHTQGVEFGSRTRNVVFDIFTGKGQDMVFT 120
Capsicum        YAKKFGDIFLLRMGQRNLVVVSSPESAKEVLHTQGVEFGSRTRNVVFDIFTGKGQDMVFT 120
Camellia        --------------------------------------------------------MVFT 4
Brassica        YAKKFGDLFLLRMGQRNLVVVSSPNLTKEVLHTQGVEFGSRTRNVVFDIFTGKGQDMVFT 120
Arabidopsis     YAKKFGDLFLLRMGQRNLVVVSSPDLTKEVLHTQGVEFGSRTRNVVFDIFTGKGQDMVFT 120
                                                                        ****

Arnebia         VYGEHWRKMRRIMTVPFFTNKVVQQYRQGWEFEVESAIEDVKKNPESETVGIVLRKRLQL 180
Lithospermum    VYGEHWRKMRRIMTVPFFTNKVVQQYRKGWESEVESVIEDVKKIPESETVGIVLRKRLQL 180
Solanum         VYGEHWRKMRRIMTVPFFTNKVVQQYRGGWESEAASVVEDVKKNPESATNGIVLRKRLQL 180
Capsicum        VYGEHWRKMRRIMTVPFFTNKVVQQYRGGWESEVASVVEDVKKNPESATNGIVLRKRLQL 180
Camellia        VYGEHWRKMRRIMTVPFFTNKVVQQYRFGWEDEAGRVVEDVKKNPEAKTNGIVLRRRLQL 64
Brassica        VYGEHWRKMRRIMTVPFFTNKVVQQNREGWEFEAASVVEDVKKNPDSATKGIVLRKRLQL 180
Arabidopsis     VYGEHWRKMRRIMTVPFFTNKVVQQNREGWEFEAASVVEDVKKNPDSATKGIVLRKRLQL 180
                ************************ * *** *.  .:***** *:: * *****:****

Arnebia         MMYNNMFRIMFDRRFESEDDPLFKKLRALNGERSRLAQSFDYNYGDFIPILRPFLRGYLK 240
Lithospermum    MMYNNMFRIMFDRRFESENDPLFMKLRALNGERSRLAQSFDYNYGDFIPILRPFLRGYLK 240
Solanum         MMYNNMFRIMFDRRFESEDDPLFVKLRALNGERSRLAQSFEYNYGDFIPILRPFLRGYLK 240
Capsicum        MMYNNMFRIMFDRRFESEDDPLFVKLRALNGERSRLAQSFEYNYGDFIPILRPFLRGYLK 240
Camellia        MMYNNMYRIMFDSRFESEEDPLFVKLKALNGERSRLAQSFEYNYGDFIPILRPFLRGYLK 124
Brassica        MMYNNMFRIMFDRRFDSEDDPLFIRLKALNGERSRLAQSFEYNYGDFIPILRPFLRGYLK 240
Arabidopsis     MMYNNMFRIMFDRRFESEDDPLFLRLKALNGERSRLAQSFEYNYGDFIPILRPFLRGYLK 240
                ******:***** **:**:**** :*:*************:******************

Arnebia         ICKEVKETRLKLFKDYFVDERKKIAS-TKSTTSNGLKCAIDHILEAQQKGEINEDNVLYI 299
Lithospermum    ICKEVKETRLKLFKDYFVEERKKIAS-TKSTTTNGLKCAIDHILEAQQKGEINEDNVLYI 299
Solanum         ICKEVKEKRLKLFKDYFVDERKKLAN-TKSMDSNALKCAIDHILEAQQKGEINEDNVLYI 299
Capsicum        ICKEVKEKRLQLFKDYFVDERKKLAN-TKSMDSNALKCAIDHILDAQQKGEINEDNVLYI 299
Camellia        ICKEVKERRLQLFKDYFVDERKKLAKPRRAMDTVTLKCAIDHILDAQQKGEINEDNVLYI 184
Brassica        ICQDVKDRRLALFKKYFVDERKQIAS-WKPTGSEGLKCAIDHILEAQQKGEINEDNVLYI 299
Arabidopsis     ICQDVKDRRIALFKKYFVDERKQIAS-SKPTGSEGLKCAIDHILEAEQKGEINEDNVLYI 299
                **::**: *: ***.***:***::*.  :.  :  *********:*:************

Arnebia         VENINVAAIETTLWSIEWGIAELVNHPRIQKKLRDEIDAILGPGVQVTEPDTHKLPYLQA 359
Lithospermum    VENINVAAIETTLWSIEWGIAELVNHPEIQKKLRDEIDTILGPGVQVTEPDTHKLPYLQA 359
Solanum         VENINVAAIETTLWSIEWGIAELVNHPHIQKKLRDEIDTVLGPGMQVTEPDMPKLPYLQA 359
Capsicum        VENINVAAIETTLWSIEWGIAELVNHPHIQQKLREEIDAVLGPGVQVTEPDTQKLPYLQA 359
Camellia        VENINVAAIETTLWSIEWGIAELVNHPQIQKKLRHELDTMLGLGVQITEPDTYKLPYLQA 244
Brassica        VENINVAAIETTLWSIEWGIAELVNHPEIQTKLRNEIDTVLGPGVQVTEPELHKLPYLQA 359
Arabidopsis     VENINVAAIETTLWSIEWGIAELVNHPEIQSKLRNELDTVLGPGVQVTEPDLHKLPYLQA 359
                **************************.** ***.*:*::** *:*:***:  *******

Arnebia         VVKETLRLRMAIPLLVPHMNLHDAKLNGYDIPAESKILVNAWWLANNPEQWKNPEEFRPE 419
Lithospermum    VIKETLRLRMAIPLLVPHMNLHDAKLNGYDIPAESKILVNAWWLANNPAQWKNPEEFRPE 419
Solanum         VIKETLRLRMAIPLLVPHMNLHDAKLAGYDIPAESKILVNAWWLANNPAHWKKPEEFRPE 419
Capsicum        VIKETLRLRMAIPLLVPHMNLHDAKLAGYDIPAESKILVNAWWLANNPAHWKKPEEFRPE 419
Camellia        VVKETLRLRMAIPLLVPHMNLHDAKLSGYDIPAESKILVNAWWLANNPDNWKNPEEFRPE 304
```

```
Brassica            VIKETLRLRMAIPLLVPHMNLNDAKLAGYDIPAESKILVNAWWLANNPESWKKPEEFRPE 419
Arabidopsis         VVKETLRLRMAIPLLVPHMNLHDAKLAGYDIPAESKILVNAWWLANNPNSWKKPEEFRPE 419
                    *:*******************:**** *********************  **:*******

Arnebia             RFLEEEAKVEANGNDFRYLPFGVGRRSCPGIILALPILGITLGRLVQNFELLPPPGQSKL 479
Lithospermum        RFLEEEAKVEANGNDFRYLPFGVGRRSCPGIILALPILGITLGRLVQNFELLPPPGQSKL 479
Solanum             RFFEEEKHVEANGNDFRFLPFGVGRRSCPGIILALPILGITLGRLVQNFEMLPPPGQSKL 479
Capsicum            RFLEEEKHVDANGNDFRFLPFGVGRRSCPGIILALPILGITLGRLVQNFELLPPPGQSKL 479
Camellia            RFLEEEAKVEANGNDFRYLPFGVGRRSCPGIILALPILGITLGRLVQNFELLPPPGQAKI 364
Brassica            RFFEEEAHVEANGNDFRYVPFGVGRRSCPGIILALPILGITIGRMVQNFELLPPPGQSKL 479
Arabidopsis         RFFEEESHVEANGNDFRYVPFGVGRRSCPGIILALPILGITIGRMVQNFELLPPPGQSKV 479
                    **:*** :*:*******::**********************:**:*****:******:*:

Arnebia             DTSEKGGQFSLHILKHSTIVMKPRA- 504
Lithospermum        DTSEKGGQFSLHILKHSTIVMKPRSL 505
Solanum             DTSEKGGQFSLHILKHSTIVMKPRSF 505
Capsicum            DTTEKGGQFSLHILKHSTIVMKPRSF 505
Camellia            DTAEKGGQFSLHILKHSTIVLKPRSF 390
Brassica            DTSEKGGQFSLHILHHSTIVMKPRSF 505
Arabidopsis         DTSEKGGQFSLHILNHSIIVMKPRNC 505
                    **:***********:** **:***
```

(l) 4-coumaric acid-CoA ligase (Ae4-CL)

```
Arnebia             ------------------MDTQTKTDQKDIIFRSKLPDIYIPRHLPLHSYCFENISQFNS 42
Lithospermum        ------------------MDTQTKTDQKDIIFRSKLPDIYIPKHLPLHSYCGENISQFSS 42
Capsicum            ----------------MPMENETR----DLIFRSKLPDIYIPKHLPLHSYCFENISEFNS 40
Solanum             ----------------MPMDTETKQSG-DLIFRSKLPDIYIPKHLPLHSYCFENLSEFNS 43
Nicotiana           ------------------MEKDTKHG--DIIFRSKLPDIYIPNHLPLHSYCFENISEFSS 40
Paulownia           ------------------METTITKQE-DIIFRSKLPDIYIPKHLPLHSYCFENISKFST 41
Arabidopsis         -------MAPQEQAVSQVMEKQSNNNNSDVIFRSKLPDIYIPNHLSLHDYIFQNISEFAT 53
Camellia            MLSGASAETPKPEFSPPPPQNPQSSSSQTHVFRSKLPHIPISNHLPLHTYRFQNLSQFPN 60
                                       :.         :******.* *..**.** *  :*:*:* .

Arnebia             RPCLINGSNDRVYTYAEVELTSRKVAAGLHKHGIKQTETIMLLLPNCPEFVFAFLGASYI 102
Lithospermum        RPCLINGSNDRVYTYAEVEITSRKVAAGLHKHGIKQTETIMLLLPNCPEFVFAFLGASYI 102
Capsicum            RPCLIDGANDQIYSYAEVELTSRKVAVGLNKLGVQQKDTIMILLPNSPEFVFAFMGASYL 100
Solanum             RPCLIDGANDRIYTYAEVELTSRKVAVGLNKLGIQQKDTIMILLPNCPEFVFAFIGASYL 103
Nicotiana           RPCLINGANKQIYTYADVELSSRKVAAGLHKQGIQQKDTIMILLPNSPEFVFAFIGASYL 100
Paulownia           RPCLINGATNEVYTYEEVELIARKVATGLSKLGIQQGDTILLLLPNSPEYVFAFLGASYI 101
Arabidopsis         KPCLINGPTGHVYTYSDVHVISRQIAANFHKLGVNQNDVVMLLLPNCPEFVLSFLAASFR 113
Camellia            RPCLLIGSTGKSYSFSXTHLVSRKVASGLSLLGIKKGDVIMLLLQNCAEFVFAFMGASMI 120
                    :***: *.. . *::  ..: :*::* .:   *::: :.:::** *..*:*::*:.**

Arnebia             GAVSTTANPFFTSSEIIKQAKASKTKLIITVASNVPKLKEFSQ-----ENGVKIMCIDEQ 157
Lithospermum        GAVSTTANPFFTSSEIIKQAKASKTKLIITVSTTVPKLKDFSQ-----ENHVKIMCIDDK 157
Capsicum            GAISTMANPLFTPAEVLKQAKASSAKIIITLACYIGKVKDYAT-----ENDVKLICIDSA 155
Solanum             GAISTMANPLFTPAEVVKQAKASSAKIIITQACFAGKVKDYAI-----ENDLKVICVDSA 158
Nicotiana           GAISTMANPLFTAAEVVKQVKASGAKIIVTQACHVNKVKDYAL-----ENNVKIICIDSA 155
Paulownia           GAISTMANPFFTPAEVIKQAKASNAKLIITQACYVEKVRDYAL-----EKGVKVMCIDAP 156
Arabidopsis         GATATAANPFFTPAEIAKQAKASNTKLIITEARYVDKIKPLQN-----DDGVVIVCIDDN 168
Camellia            GAVTTTANPFYTSAEVFKQFNASKSKLIITHSQYVDKLRDAGDNFPNWAKEFSVITIDDP 180
                    ** :* ***::*.:*: ** :** :*:*:* :    *::          . . :: :*

Arnebia             -----IEGCLHFSLDLENTDETT---LPEVEILPNDVVALPYSSGTTGLPKGVMLTHKGL 209
Lithospermum        -----IDGCLHFSSDLENSDETT---LPDVEIRPDDVVALPYSSGTTGLPKGVMLTHKGL 209
Capsicum            -----PEGCIHFS-ELTQSDEHE---IPDVKIQPDDVVALPYSSGTTGLPKGVMLTHKGL 206
Solanum             -----PEGCVHFS-ELIQSDEHE---IPDVKIQPDDVVALPYSSGTTGLPKGVMLTHKGL 209
Nicotiana           -----PEGCLHFS-VLTQADEHD---IPEVEIQPDDVVALPYSSGTTGLPKGVMLTHKGL 206
Paulownia           -----SADCLQFS-ELTSADERD---MPAVKIHPDDVVALPYSSGTTGLPKGVMLTHKGL 207
Arabidopsis         ESVPIPEGCLRFT-ELTQSTTEASEVIDSVEISPDDVVALPYSSGTTGLPKGVMLTHKGL 227
Camellia            -----PENCLHFS-VLSEANESE---IPTVSIDPDDPVALPFSSGTTGLPKGVILTHKSL 231
                           .*::*:  * .:       :  *.* *:* ****:***********:****.*

Arnebia             VTSVAQQVDGENANLYMHQEDVVMCTLPLFHIYSMNSILLCGLRVGAAILLMHKFDIAPF 269
Lithospermum        VTSVAQQVDGDNANLYMHHEDVVMCTLPLFHIYSMNSILLCGLRVGAAILLMHKFEIVTF 269
Capsicum            VTSVAQQVDGENANLYMHSEDVLMCCLPLFHIYSLNSVLLCGLRIGAAILIMQKFDIVHF 266
Solanum             VTSVAQQVDGENANLYMHSDDVLMCVLPLFHIYSLNSVLLCALRVGAAILIMQKFDIAQF 269
Nicotiana           VTSVAQQVDGENRNLYIHSEDVLLCVLPLFHIYSLNSVLLCGLRVGAAILIMQKFDIVPF 266
Paulownia           VTSVAQQVDGENPNLYIHSEDVMLCVLPLFHIYSLNSVLLCGLRVGAAILIMQKFDIVPF 267
Arabidopsis         VTSVAQQVDGENPNLYFHSDDVILCVLPMFHIYALNSIMLCGLRVGAAILIMPKFEINLL 287
Camellia            ISSVAQQVDGENPNLRLKGEDMVLCVLPLFHIYSLNSVLLCSLRAGTGVLLMHKFEIGAL 291
                    ::********:* ** :: :*:::* **:****::**::**.** *:.:*:* **:*  :
```

```
Arnebia          LELIQRYKVTIGPFVPPIVLAIAKSNVVDQFDLSSVRTVMSGAAPLGKELEDAVRAKFPN 329
Lithospermum     LELIQRYKVTIGPFVPPIVLAIAKSNVVDQYDLSTVRTVMSGAAPLGSELEDAVRAKFPN 329
Capsicum         LELIQKYKVTIGPFVPPIVLAIAKSPLVDHYDLSSVRTVMSGAAPLGKELEDTVRTKFPN 326
Solanum          LELIPKHKVTIGPFVPPIVLAIAKSPLVHNYDLSSVRTVMSGAAPLGKELEDAVRAKFPN 329
Nicotiana        LELIQNYKVTIGPFVPPIVLAIAKSPMVDDYDLSSVRTVMSGAAPLGKELEDTVRAKFPN 326
Paulownia        LELIQKYKVTIGPFVPPIVLAIVKSPVVDKYDLSSVRTVMSGAAPLGKELEDAVRIKFPN 327
Arabidopsis      LELIQRCKVTVAPMVPPIVLAIAKSSETEKYDLSSIRVVKSGAAPLGKELEDAVNAKFPN 347
Camellia         LELIQRHRVSVAAVVPPLVLALAKNPMVVTFDLSSIRMVLSGAAPLGKELEEALRARVPQ 351
                 **** . :*::...***:***:.*.  .  :***::* * *******.***:::. :.*:

Arnebia          AKLGQGYGMTEAGPVLAMCLAFAKEPFEIKSGACGTVVRNAEMKIIDTETGVSLPRNQSG 389
Lithospermum     AKLGQGYGMTEAGPVLAMCLAFAKEPFEIKSGACGTVVRNSEMKIIDTETGASLPRNQSG 389
Capsicum         AKLGQGYGMTEAGPVLAMCLAFAKEPFEIKSGACGTVVRNAEMKIVDPDTGCSLPRNQPG 386
Solanum          AKLGQGYGMTEAGTVLTMCLAFAKEPFDIKSGACGTVVRNAEMKIVDPDTGCSLPRNQPG 389
Nicotiana        AKLGQGYGMTEAGPVLAMCLAFAKEPFEIKSGACGTVVRNAEMKIVDPETGNSLPRNQSG 386
Paulownia        AKLGQGYGMTEAGPVLAMCLAFAKEPFEIKSGACGTVVRNAEMKIVDIETGASLGRNQPG 387
Arabidopsis      AKLGQGYGMTEAGPVLAMSLGFAKEPFPVKSGACGTVVRNAEMKIVDPDTGDSLSRNQPG 407
Camellia         AIFGQGYGMTEAGPVLSMCLAFAKQPFPTKSGSCGTVVRNAELKVIDPETGCSLGPNHSG 411
                 * :**********.**:*.*.***:**  ***:*******:*:*::* :** **  *:.*

Arnebia          EICIRGDQIMKGYLNDPEATERTIDNEGWLHTGDIGYIDDDDELFIVDRLKELIKYKGFQ 449
Lithospermum     EICIRGDQIMKGYLNDPEATERTIDKEGWLHTGDIGYIDDDDELFIVDRLKELIKYKGFQ 449
Capsicum         EICIRGDQIMKGYLNDLESTTRTIDKEGWLHTGDMGFIDNDDELFIVDRLKELIKYKGFQ 446
Solanum          EICIRGDQIMKGYLNDPEATARTIEEEGWLHTGDIGFIDDDDELFIVDRLKELIKYKGFQ 449
Nicotiana        EICIRGDQIMKGYLNDPEATARTIDKEGWLYTGDIGYIDDDDELFIVDRLKELIKYKGFQ 446
Paulownia        EICIRGDQIMKGYLNDLESTEGTIDKDGWLHTGDIGFIDTDDELFIVDRLKEIIKYKGFQ 447
Arabidopsis      EICIRGHQIMKGYLNNPAATAETIDKDGWLHTGDIGLIDDDDELFIVDRLKELIKYKGFQ 467
Camellia         EICIRGPQIMKGYLNHAEATATTIDVDGWLHTGDIGYVDDDDE-VIVDRVKELIKFKGFQ 470
                 ****** ********.  :*  **: :***:***:* :* *** .****:**:**:****

Arnebia          VAPAELEALLVTHPKVSDAAVVSMKDEGAGEVPVAFVVRSNGSTITEDEIKQFVSKQVIF 509
Lithospermum     VAPPELEALLVPHPNVSDAAVVSMKDEGAGEVPVAFVVRSNGSTTTEDEIKQFVSKQVIF 509
Capsicum         VAPAELEALLLNHPNISDAAVVPMKDEQAGEVPVAFVVRSNGSTITEDEVKDFVSKQVVF 506
Solanum          VAPAELEALLINHPDISDAAVVPMIDEQAGEVPVAFVVRSNGSTITEDEVKDFISKQVIF 509
Nicotiana        VAPAELEALLLNHPTFSDAAVVPMKDEQAEEVPVAFVVRSSGSTITEDEVKDFISKQVIF 506
Paulownia        VAPAELEALLLNHPYISDAAVVPMKDEQAGEVPVAFVVRSNGSTITEDEIKQFISKQVVF 507
Arabidopsis      VAPAELEALLIGHPDITDVAVVAMKEEEAGEVPVAFVVKSKDSELSEDDVKQFVSKQVVF 527
Camellia         VPPAELEALLVSHPSIADAAVVPQKDDVAGEVPVAFVVRSNGLELTEDAVKEFIAKQVVF 530
                 *.*.******: ** .:*.***.  :: * ********:*..   :** :*:*::***:*

Arnebia          YKRINRVFFVDSIPKSP-SGKILRKDLRAKLAAGFLNGPTTTTNGVPNSG-VAKD-VPNG 566
Lithospermum     YKRINRVFGVDSIPKSP-SGKIVRKDLRAKLAARFLNGP--TTNVVPNGGNVAKDNVPNG 566
Capsicum         YKRIKRVFFVETVPKSP-SGKILRKDLRARLAAGVTN----------------------- 542
Solanum          YKRIKRVFFVETVPKSP-SGKILRKDLRARLAAGISN----------------------- 545
Nicotiana        YKRIKRVFFVDAVPKSP-SGKILRKDLRAKLAAGLPN----------------------- 542
Paulownia        YKRINRVFFIDAIPKSP-SGKILRKDLRARLAAGVPN----------------------- 543
Arabidopsis      YKRINKVFFTESIPKAP-SGKILRKDLRAK------------------------------ 556
Camellia         YKKLHKVYFVPCHSQSLLSGKILRKDLRAKLGHVRSLSNSLETIYMYLFDFHLRVSIN-- 588
                 **::::*:     .::  ****:******:

Arnebia          VSNGVSKANGGGIEEGIANGVAKANGGVAKEEIANGVSKANCGVAKEEIANGIPTSVANG 626
Lithospermum     VSNGVSKANGGVAKEGVANGVPTDG---------------DYGVATKGVANGISN----- 606
Capsicum         ------------------------------------------------------------
Solanum          ------------------------------------------------------------
Nicotiana        ------------------------------------------------------------
Paulownia        ------------------------------------------------------------
Arabidopsis      ------------------------------------------------------------
Camellia         ------------------------------------------------------------


Arnebia          VATKGVYKQKINGVVSNGVENGVVSNGTTNGVHN 660
Lithospermum     ----GVYKQVSNGVVSNGVANGIVSNGIANGVHN 636
Capsicum         ----------------------------------
Solanum          ----------------------------------
Nicotiana        ----------------------------------
Paulownia        ----------------------------------
Arabidopsis      ----------------------------------
Camellia         ----------------------------------
```
