# Supplementary figures and images for: Expression of 3-hydroxy-3-methylglutaryl-CoA reductase, p-hydroxybenzoate-m-geranyltransferase and genes of phenylpropanoid pathway exhibits positive correlation with shikonins content in arnebia [Arnebia euchroma (Royle) Johnston]
Source: BMC Mol Biol. 2010 Nov 21;11:88. doi: 10.1186/1471-2199-11-88 (PMC3002352; doi:10.1186/1471-2199-11-88)

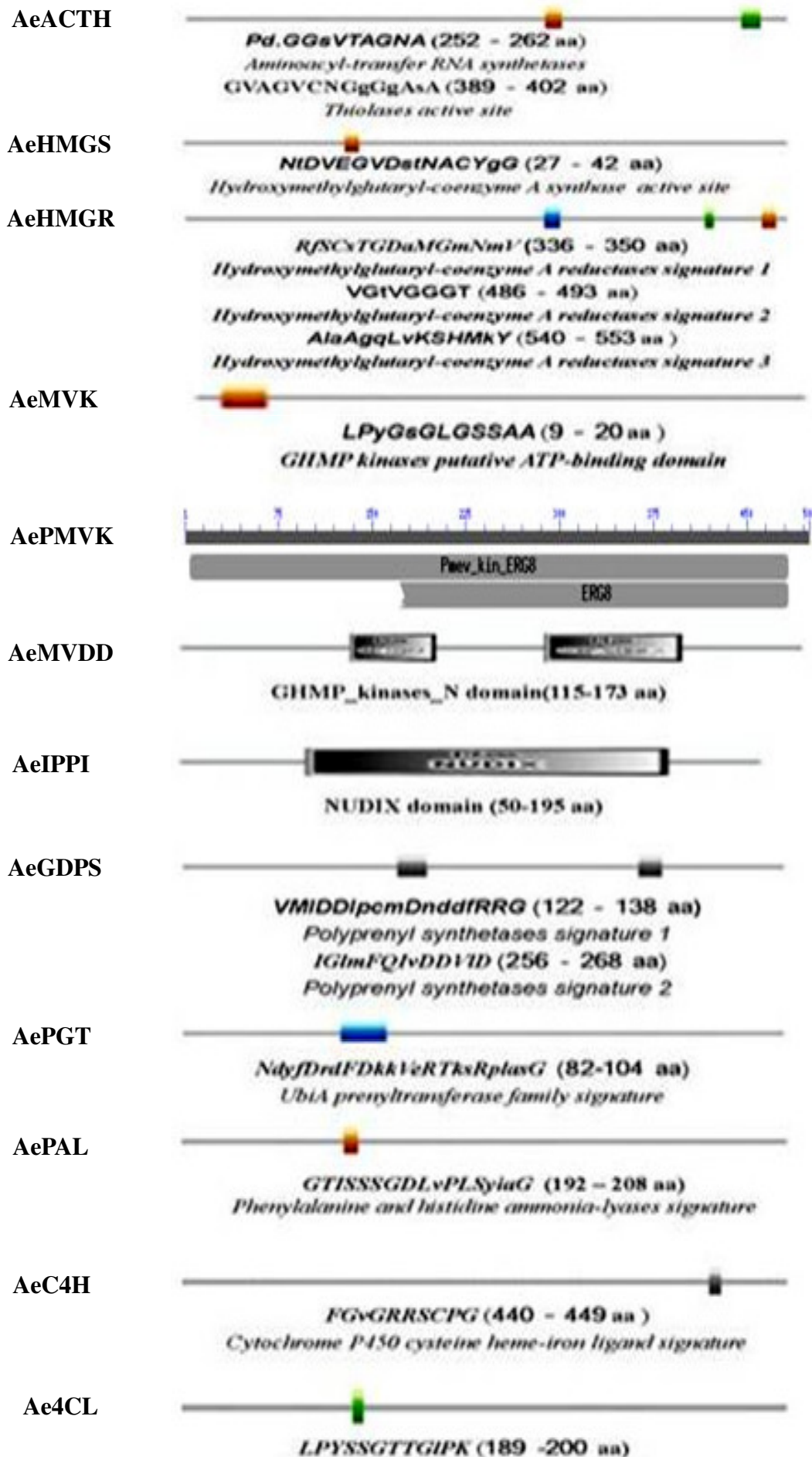

Supplement: Additional file 5 — Domain and protein families in the deduced amino acid sequences of arnebia cDNAs. Domain and protein families in the deduced amino acid sequences of AeACTH, AeHMGS, AeHMGR, AeMVK, AePMVK, AeMVDD, AeGDPS, AeIPPI, AePGT, AePAL, AeC4H, and Ae4-CL. [file 1471-2199-11-88-S5.PDF]

Additional file 6: Supplementary Figure S4.

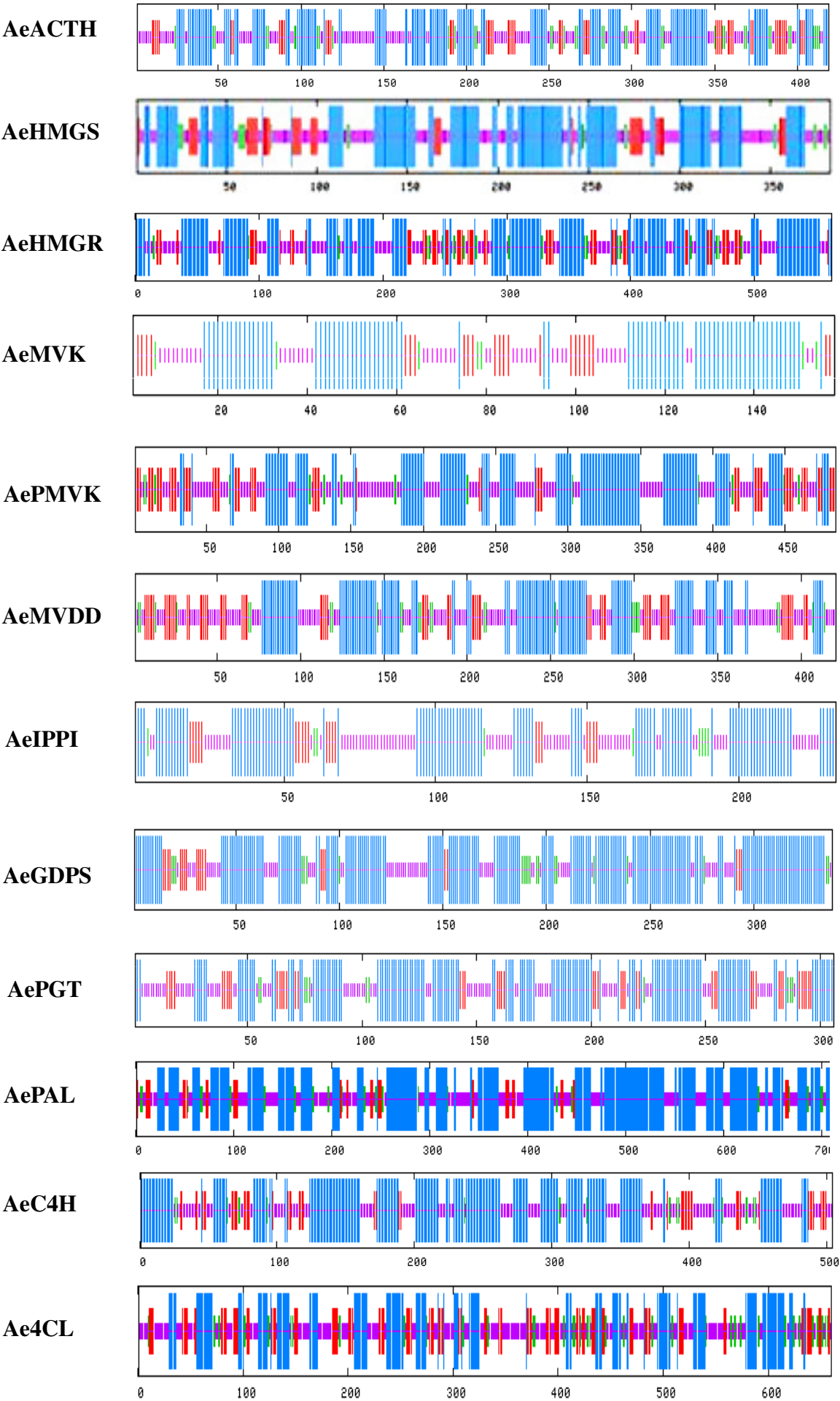

Supplement: Additional file 6 — Prediction of secondary structure of deduced amino acid sequences. Prediction of secondary structure of deduced amino acid sequences of AeACTH, AeHMGS, AeHMGR, AeMVK, AePMVK, AeMVDD, AeGDPS, AeIPPI, AePGT, AePAL, AeC4H and Ae4-CL by SOPMA. Helices, sheets, turns and coils are indicated by the longest, the second longest, the second shortest and the shortest vertical lines, respectively. [file 1471-2199-11-88-S6.PDF]

**Additional file 7:** Supplementary Figure S5.

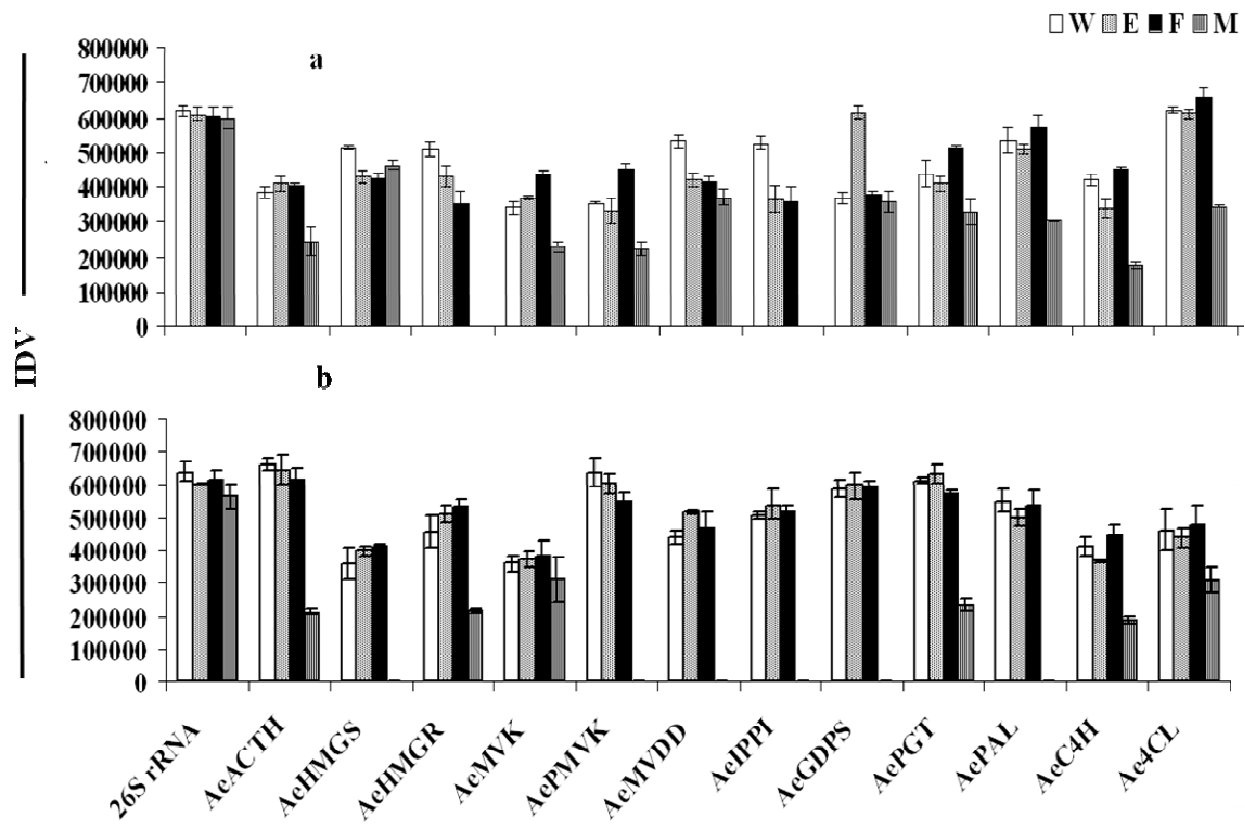

Supplement: Additional file 7 — Bar diagram indicating intensities (integrated density value; IDV) of the amplicons of Figure 2b at day 4 and day 8 (panel b) as measured using Alpha DigiDoc 1000 software. Bar diagram indicating intensities (integrated density value; IDV) of the amplicons of Figure 2b at day 4 and day 8 (panel b) as measured using Alpha DigiDoc 1000 software. Error bar shows standard deviation of three separate values. Since the IDV of separate gels were very different, these were normalized based upon the amplicons for 26 S rRNA. [file 1471-2199-11-88-S7.PDF]

Additional file 8: Supplementary Figure S6.

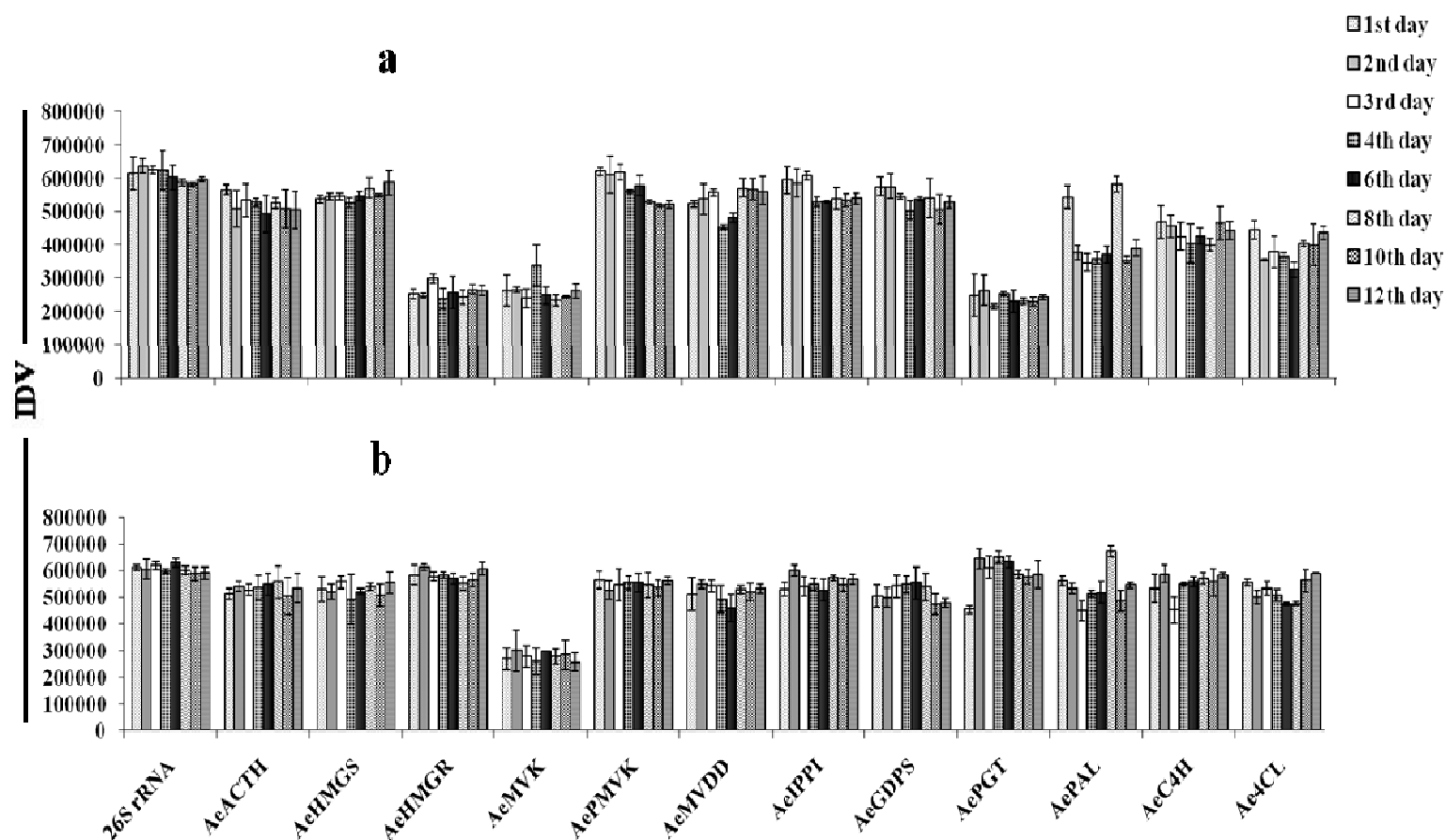

Supplement: Additional file 8 — Bar diagram indicating intensities (integrated density value; IDV) of the amplicons of Figure 3a and 3b as measured using Alpha DigiDoc 1000 software. Bar diagram indicating intensities (integrated density value; IDV) of the amplicons of Figure 3a and 3b as measured using Alpha DigiDoc 1000 software. Error bar shows standard deviation of three separate values. Since the IDV of separate gels were very different, these were normalized based upon the amplicons for 26 S rRNA. [file 1471-2199-11-88-S8.PDF]
