# Supplementary material for: Expression of 3-hydroxy-3-methylglutaryl-CoA reductase, p-hydroxybenzoate-m-geranyltransferase and genes of phenylpropanoid pathway exhibits positive correlation with shikonins content in arnebia [Arnebia euchroma (Royle) Johnston]
Source: BMC Mol Biol. 2010 Nov 21;11:88. doi: 10.1186/1471-2199-11-88 (PMC3002352; doi:10.1186/1471-2199-11-88)
Supplement: Additional file 9 — Primer sequences and PCR conditions used in RACE reactions. Primer sequences and PCR conditions used in RACE reactions for cloning of full-length cDNAs of AeACTH, AeHMGS, AeHMGR, AePMVK, AeMVDD, AeGDPS, AeIPPI, AePGT, AePAL, AeC4H and Ae4-CL. [file 1471-2199-11-88-S9.PDF]

Additional file 9: Supplementary Table S3. Primer sequences and PCR conditions used in RACE reactions.

GSP1, gene specific primer for 3′-RACE; NGSP1, nested gene specific primer for 3′-RACE.

GSP2, gene specific primer for 5′-RACE; NGSP2, nested gene specific primer for 3′-RACE.

Name of the gene	Primers Sequences	PCR conditions
AeACTH	GSP1: 5′-TCAAGAATGGGATCACGGCTAGGACA-3′<br>NGSP1: 5′-CCTGGGGAAATAGTTCCGGTTGAGCTT-3′<br>GSP2: 5′-GGGTATTCCAGCACCTAATGCAGCTTG-3′<br>NGSP2: 5′-GCCTCCGGCAACTACGACATTATTGCT-3′	Primary PCR<br>5 cycles: 94ºC, 30 sec; 72 ºC, 3 min; followed by 5 cycles: 94ºC, 30 sec; 70ºC, 30 sec; 72 ºC, 3 min; and 30 cycles: 94ºC, 30 sec; 68ºC, 30 sec; 72 ºC, 3min; Final extension at 72ºC, 7 min<br>Nested PCR<br>30 cycles: 94ºC, 30 sec; 68ºC, 30 sec; 72 ºC, 3 min; Final extension at 72ºC, 7 min
AeHMGS	GSP1: 5'-GTAGGAAGTGAGACTGTTATTGACAA-3′<br>NGSP1: 5'-TGTCAATTGGGTTGAGAGTAAC-3′<br>GSP2: 5'-CACCACTTTTGATCTTCCTTACAAT-3′<br>NGSP2: 5'-CCTACATATCAAATGAAGCAACTCA-3′	Primary PCR<br>5 cycles: 94ºC, 30 sec; 72 ºC, 3 min; followed by 5 cycles: 94ºC, 30 sec; 70ºC, 30 sec; 72 ºC, 3 min; and 30 cycles: 94ºC, 30 sec; 68ºC, 30 sec; 72 ºC, 3min; Final extension at 72ºC, 7 min<br>Nested PCR<br>30 cycles: 94ºC, 30 sec; 68ºC, 30 sec; 72 ºC, 3 min; Final extension at 72ºC, 7 min
AeHMGR	GSP1: 5′- TGGAGGACCCTCTCAATTTCGAGACA -3′<br>NGSP1: 5′- ACTTCAGAGCATTAAGTGCGCCATC-3′<br>GSP2: 5′- GCACACGACTGATTTTCCACGTCCTT-3′<br>NGSP2: 5′-AGTTGCCAGAAATGCCAATGACATC-3′	Primary PCR<br>5 cycles: 94ºC, 30 sec; 72 ºC, 3 min; followed by 5 cycles: 94ºC, 30 sec; 70ºC, 30 sec; 72 ºC, 3 min; and 30 cycles: 94ºC, 30 sec; 68ºC, 30 sec; 72 ºC, 3min; Final extension at 72ºC, 7 min<br>Nested PCR<br>30 cycles: 94ºC, 30 sec; 68ºC, 30 sec; 72 ºC, 3 min; Final extension at 72ºC, 7 min
AePMVK	GSP1: 5'-ATCTTGTTTTGGAGAGGCCAAATGCTG−3′<br>NGSP1: 5'-CCAAATGCTGGAATTGTTTTGAGTAC−3′<br>GSP2: 5'-ACATCTGCCCATGCCCAAGCCCAACTG−3′<br>NGSP2: 5'-CAGCATTTGGCCTCTCCAAAACAAGA−3′	Primary PCR<br>5 cycles: 94ºC, 30 sec; 72 ºC, 3 min; followed by 5 cycles: 94ºC, 30 sec; 70ºC, 30 sec; 72 ºC, 3 min; and 30 cycles: 94ºC, 30 sec; 68ºC, 30 sec; 72 ºC, 3min; Final extension at 72ºC, 7 min<br>Nested PCR<br>30 cycles: 94ºC, 30 sec; 68ºC, 30 sec; 72 ºC, 3 min; Final extension at 72ºC, 7 min
AeMVDD	GSP1: 5′-CTTCCTCAGCTGCTGGTTTTGCCTGT-3′<br>NGSP1: 5′-TTGTCGGCTATCGCTAGGCAAGGTTC-3′<br>GSP2: 5′-ATCACGATTGCTTATGGCCTCCTCCA-3′<br>NGSP2: 5′-GATGCGTTTGGGCACTACTTCCTTCG-3′	Primary PCR<br>5 cycles: 94ºC, 30 sec; 72 ºC, 3 min; followed by 5 cycles: 94ºC, 30 sec; 70ºC, 30 sec; 72 ºC, 3 min; and 30 cycles: 94ºC, 30 sec; 68ºC, 30 sec; 72 ºC, 3min; Final extension at 72ºC, 7 min<br>Nested PCR<br>30 cycles: 94ºC, 30 sec; 68ºC, 30 sec; 72 ºC, 3 min; Final extension at 72ºC, 7 min
AeIPPI	GSP1: 5′-AAATGATCGTGTGGTGGGACATGACA-3′<br>NGSP1: 5′-ATGAGTTGCTTCTCCAGCAACGGTCA-3′<br>GSP2: 5′-GAGAAGCTTCCTTTGTGCGGCATTTC-3′<br>NGSP2: 5′-TTTGTGCGGCATTTCTTACACCAAGC-3′	Primary PCR<br>5 cycles: 94ºC, 30 sec; 72 ºC, 3 min; followed by 5 cycles: 94ºC, 30 sec; 70ºC, 30 sec; 72 ºC, 3 min; and 30 cycles: 94ºC, 30 sec; 68ºC, 30 sec; 72 ºC, 3min; Final extension at 72ºC, 7 min<br>Nested PCR<br>30 cycles: 94ºC, 30 sec; 68ºC, 30 sec; 72 ºC, 3 min; Final extension at 72ºC, 7 min
AeGDPS	GSP1: 5′-AACAGTGATGCCAACAGCCTGTGCT-3′<br>NGSP1: 5′-GGGGTGCGTCTGAAGAGGAAATTGA-3′<br>GSP2: 5′-AGCACAGGCTGTTGGCATCACTGTT-3′<br>NGSP2: 5′-TCATGGCCTCCAACAAGCTCACAAG-3′	Primary PCR<br>5 cycles: 94ºC, 30 sec; 72 ºC, 3 min; followed by 5 cycles: 94ºC, 30 sec; 70ºC, 30 sec; 72 ºC, 3 min; and 30 cycles: 94ºC, 30 sec; 68ºC, 30 sec; 72 ºC, 3min; Final extension at 72ºC, 7 min<br>Nested PCR<br>30 cycles: 94ºC, 30 sec; 68ºC, 30 sec; 72 ºC, 3 min; Final extension at 72ºC, 7 min
AePGT	GSP1: 5′- GGTGGCTTGCATTTCAGCTGTTCATT -3′<br>NGSP1: 5′-GGAGCTCTCTTAGGCTCCTCTGCT-3′<br>GSP2: 5′- GAGCAGCAATGCATCCTACTCCGAAC-3′<br>NGSP2: 5′-GAGCCTAAGAGAGCTCCCCAACTGA-3′	Primary PCR<br>5 cycles: 94ºC, 30 sec; 72 ºC, 3 min; followed by 5 cycles: 94ºC, 30 sec; 70ºC, 30 sec; 72 ºC, 3 min; and 30 cycles: 94ºC, 30 sec; 68ºC, 30 sec; 72 ºC, 3min; Final extension at 72ºC, 7 min<br>Nested PCR<br>30 cycles: 94ºC, 30 sec; 68ºC, 30 sec; 72 ºC, 3 min; Final extension at

		72ºC, 7 min
AePAL	GSP1: 5′-ATGAAGTGAAACGCATGGTGGCTGA-3′<br>NGSP1: 5′-CACATGACGACGGAGTCAAGGTGGA-3′<br>GSP2: 5′-TTGGTGATGGCTTCCAGGATCTCAA-3′<br>NGSP2: 5′-GCCTTAACACCTTCCCTGGCTGCTT-3′	Primary PCR<br>5 cycles: 94ºC, 30 sec; 72 ºC, 3 min; followed by 5 cycles: 94ºC, 30 sec; 70ºC, 30 sec; 72 ºC, 3 min; and 30 cycles: 94ºC, 30 sec; 68ºC, 30 sec; 72 ºC, 3min; Final extension at 72ºC, 7 min<br>Nested PCR<br>30 cycles: 94ºC, 30 sec; 68ºC, 30 sec; 72 ºC, 3 min; Final extension at 72ºC, 7 min
AeC4H	GSP1: 5′-TTCTTGGGATCACATTGGGACGTTT-3′<br>NGSP1: 5′-TCTTGGGATCACATTGGGACGTTTG-3′<br>GSP2: 5′-ATGCAGACTGAATTGCCCACCTTTC-3′<br>NGSP2: 5′-TGCAGACTGAATTGCCCACCTTTCT-3′	Primary PCR<br>5 cycles: 94ºC, 30 sec; 72 ºC, 3 min; followed by 5 cycles: 94ºC, 30 sec; 70ºC, 30 sec; 72 ºC, 3 min; and 30 cycles: 94ºC, 30 sec; 68ºC, 30 sec; 72 ºC, 3min; Final extension at 72ºC, 7 min<br>Nested PCR<br>30 cycles: 94ºC, 30 sec; 68ºC, 30 sec; 72 ºC, 3 min; Final extension at 72ºC, 7 min
Ae4-CL	GSP1: 5′-TCAAGACCGTGCTTAATCAATGGCTCA-3′<br>NGSP1: 5′-GCTCCCAAATTGCCCTGAATTTGTTTT-3′<br>GSP2: 5′-GGTGGTTTTCGTCTGTGTTTTCCAGGTC-3′<br>NGSP2: 5′-CTTGAGTTTTGGGGCATTGCTTGCTAC-3′	Primary PCR<br>5 cycles: 94ºC, 30 sec; 72 ºC, 3 min; followed by 5 cycles: 94ºC, 30 sec; 70ºC, 30 sec; 72 ºC, 3 min; and 30 cycles: 94ºC, 30 sec; 68ºC, 30 sec; 72 ºC, 3min; Final extension at 72ºC, 7 min<br>Nested PCR<br>30 cycles: 94ºC, 30 sec; 68ºC, 30 sec; 72 ºC, 3 min; Final extension at 72ºC, 7 min
Universal primer mix (UPM): supplied by the manufacturer<br>5′-CTAATACGACTCACTATAGGGCAAGCAGTGGTATCAACGCAGAGT-3′<br>5′-CTAATACGACTCACTATAGGGC-3′		
Nested universal primer (NUP): supplied by the manufacturer<br>5′-AAGCAGTGGTATCAACGCAGAGT-3′		
