# Supplementary material for: Expression of 3-hydroxy-3-methylglutaryl-CoA reductase, p-hydroxybenzoate-m-geranyltransferase and genes of phenylpropanoid pathway exhibits positive correlation with shikonins content in arnebia [Arnebia euchroma (Royle) Johnston]
Source: BMC Mol Biol. 2010 Nov 21;11:88. doi: 10.1186/1471-2199-11-88 (PMC3002352; doi:10.1186/1471-2199-11-88)
Supplement: Additional file 10 — Primer sequences and PCR conditions used in semi-quantitative RT-PCR-based expression analysis. Primer sequences and PCR conditions used in semi-quantitative RT-PCR-based expression analysis of AeACTH, AeHMGS, AeHMGR, AeMVK, AePMVK, AeMVDD, AeGDPS, AeIPPI, AePGT, AePAL, AeC4H, and Ae4-CL in arnebia. [file 1471-2199-11-88-S10.PDF]

Additional file 10: Supplementary Table S4. Primer sequences and PCR conditions used in semi-quantitative RT-PCR–based expression analysis.

Name of the gene	Sequence (Forward primer, F and Reverse primer, R)	PCR conditions
26S rRNA	F: 5′-CACAATGATAGGAAGAGCCGAC-3′<br>R: 5′-CAAGGGAACGGGCTTGGCAGAATC-3′	25 cycles: 94ºC, 30 sec; 52ºC, 40 sec; 72 ºC, 1 min; Final extension at 72ºC, 7 min
AeACTH	F: 5′CAGGGTTGCCTAATACAGTTAGT-3′<br>R: 5′-CACTTCAACAGGTACAATCTCC-3′	25 cycles: 94ºC, 30 sec; 52ºC, 40 sec; 72 ºC, 1 min; Final extension at 72ºC, 7 min
AeHMGS	F: 5′-GTGTTTCACTCTCCATACAAC-3′<br>R: 5′-TAAGATCGACAAATTTCTTCT-3′	29 cycles: 94ºC, 30 sec; 52ºC, 40 sec; 72 ºC, 1 min; Final extension at 72ºC, 7 min
AeHMGR	F: 5′- TCTAGGAACTCAATTACATGTTG-3′<br>R: 5′- AGCACTTTGATTACGACTCTATT-3′	30 cycles: 94ºC, 30 sec; 52ºC, 40 sec; 72 ºC, 1 min; Final extension at 72ºC, 7 min
AePMVK	F: 5′-TTTATTCGGTTGTTAAGCCAATT-3′<br>R: 5′-TTGAGTACGAATGCTCGATTTTA-3′	34 cycles: 94ºC, 30 sec; 52ºC, 40 sec; 72 ºC, 1 min; Final extension at 72ºC, 7 min
AeMVDD	F: 5′-CAAGTTAATGAATTTGAAAGAAG-3′<br>R: 5′-GACTAGTATCTAGGCAAACAGC-3′	26 cycles: 94ºC, 30 sec; 52ºC, 40 sec; 72 ºC, 1 min; Final extension at 72ºC, 7 min
AeIPPI	F: 5′-TACAATTGTCACTTGATGGA-3′<br>R: 5′-ACATACTTTATATCAGCCACCT-3′	25 cycles: 94ºC, 30 sec; 52ºC, 40 sec; 72 ºC, 1 min; Final extension at 72ºC, 7 min
AeGDPS	F: 5′-CTGTTAGATCACTTCAGGCTTT-3′<br>R: 5′-GCTCCATTGACGATTTAGTAA-3′	31 cycles: 94ºC, 30 sec; 52ºC, 40 sec; 72 ºC, 1 min; Final extension at 72ºC, 7 min
AePGT	F: 5′-ATACATAATAAGGAAACCCAATG-3′<br>R: 5′-AACGTACAAAATCTAGACCACTT-3′	31 cycles: 94ºC, 30 sec; 52ºC, 40 sec; 72 ºC, 1 min; Final extension at 72ºC, 7 min
AePAL	F: 5′-TATAGAGGAGAATGTGAGACTCG-3′<br>R: 5′-GTATGATCTGCATTCCTCAATC-3′	25 cycles: 94ºC, 30 sec; 52ºC, 40 sec; 72 ºC, 1 min; Final extension at 72ºC, 7 min
AeC4H	F: 5′-TCTTGGGATCACATTGGGAC-3′<br>R: 5′-AGGAAATAATGAGAGAGATGG-3′	25 cycles: 94ºC, 30 sec; 52ºC, 40 sec; 72 ºC, 1 min;Final extension at 72ºC, 7 min
Ae4-CL	F: 5′-GACACTCAAACCAAAACAGAC-3′<br>R: 5′-GTCTGGCAAGGTGGTTTCGTCT-3′	34 cycles: 94ºC, 30 sec; 52ºC, 40 sec; 72 ºC, 1 min; Final extension at 72ºC, 7 min
